# Inorganic benzenes with inverted singlet-triplet gaps

**DOI:** 10.1038/s42004-026-02141-0

**Published:** 2026-07-18

**Authors:** Katsuyuki Shizu, Kuraudo Ishihara, Hiroki Uratani, Hironori Kaji

**Affiliations:** 1 Institute for Chemical Research, Kyoto University, Uji, Kyoto, Japan; 2 Department of Molecular Engineering, Graduate School of Engineering, Kyoto University, Kyoto, Japan

**Keywords:** Materials for devices, Materials chemistry

## Abstract

Inorganic-type benzenes have attracted significant attention due to their unique physical and chemical properties that are different from organic benzene. In this study, we theoretically investigate the possibility of inorganic benzenes as wide-gap materials with inverted singlet and triplet (iST) excited states. Since iST materials are expected to allow extremely efficient triplet to singlet conversion, they are promising as fluorescent and assist dopant materials for light-emitting diodes. From theoretical calculations, borazine (B_3_N_3_H_6_), one of the most well-known inorganic benzenes, and its derivatives are expected not only to show iST but also to exhibit fast radiative decays surpassing those of typical iST materials: azaphenalene derivatives. Borthiin (B_3_S_3_H_3_), boroselenol (B_3_Se_3_H_3_), and the gallium-nitride analogue of benzene (Ga_3_N_3_H_6_) are also expected to show iST with wide optical gaps.

## Abstract (web summary)

Inorganic benzenes are theoretically shown to possess inverted singlet–triplet (iST) gaps, enabling efficient triplet-to-singlet conversion with large optical gaps suitable for ultraviolet electroluminescence. Among iST compounds in this study, several borazinebased frameworks exhibit fluorescence rate constants up to 10^7^–10^8 ^s^-1^, highlighting their potential as highly efficient iST emitters and as wide-gap host materials for future organic light-emitting diodes.

## Introduction

**Fig. 1:**
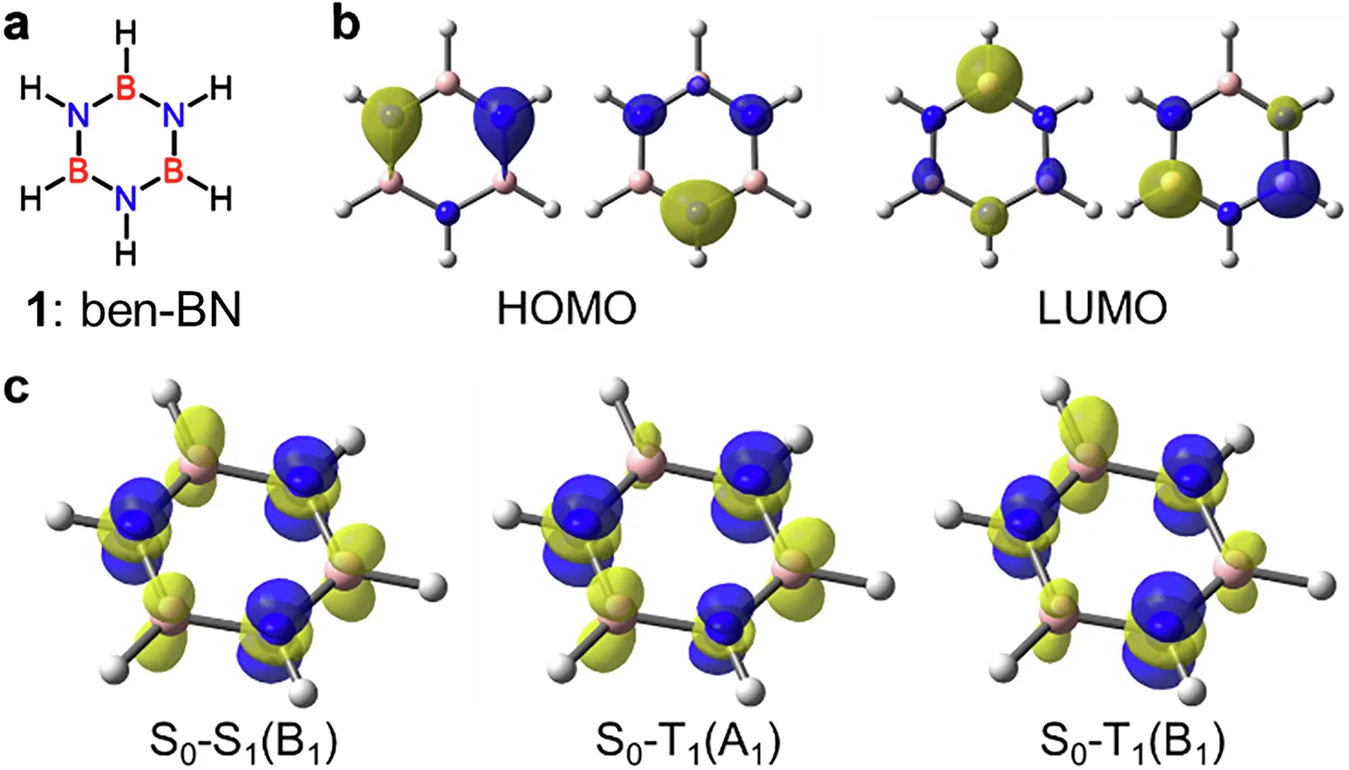
Short-range charge-transfer electronic excitations in borazine. **a** Chemical structure of borazine (**1:** benzene-type boron nitride, **ben-BN**). **b** Frontier orbitals of **1: ben-BN** calculated at the HF/cc-pVTZ//PBE0/6-31G(d) level of the theory. Two MOs are degenerate in both HOMO and LUMO. **c** Electron-density difference (Δ*ρ*) maps arising from the S_0_→S_1_(B_1_), S_0_→T_1_(A_1_), and S_0_→T_1_(B_1_) excitations in **1: ben-BN**. Here, T_1_(A_1_) and T_1_(B_1_) represent doubly degenerate T_1_ of different symmetries, A_1_ and B_1_, respectively. Blue regions show negative Δ*ρ*, where the electron density decreases. Yellow regions show positive Δ*ρ*, where the electron density increases.

Inorganic-type benzenes are six-membered six-π-electron systems having electronic structures analogous to organic benzene (hereafter referred to simply as benzene as usual)^1^. Borazine (B_3_N_3_H_6_) is one of the most representative inorganic benzenes, consisting of alternating boron (B) and nitrogen (N) atoms arranged in a hexagonal ring (Fig. 1a)^2^. While numerous materials exhibiting visible to near-infrared emission have been widely explored for organic light-emitting diodes (OLEDs), those with ultraviolet (UV) to deep-UV (DUV) emission for OLEDs remain underdeveloped, despite their broad potential across wide variety of applications^3^. Hexagonal boron nitride (hBN), a structural analog of graphene, is known to be a semiconductor that shows a wide optical band gap and is promising as a UV to DUV emitter in optoelectronic applications^4,5^. Boron nitride nanotubes (BNNTs)^6^, structural analogs of carbon nanotubes, also show wide band gaps suitable for such applications^7^. Recent advances in the synthesis makes it possible to develop diverse inorganic benzenes, including boron-nitride-based compounds consisting of four-B and two-N atoms^8^ (B_4_N_2_R_2_R’_2_R”_2_), boroxine^9^ (B_3_O_3_H_3_), borthiin^10^ (B_3_S_3_R_3_), boroselenol^11^ (B_3_Se_3_R_3_), phosphaborazine^12^ (B_3_P_3_R_3_R’_3_), phosphazene^13^ (N_3_P_3_R_6_) and its gallium analog^14^ (Ga_3_P_3_R_3_R’_3_), aluminum analog (alumazene^15^; Al_3_N_3_R_3_R’_3_) and its phosphor and arsenic analogs^16^ (Al_3_P_3_R_3_R’_3_ and Al_3_As_3_R_3_R’_3_, respectively), as well as triphospha- and triarsa-trisilabenzenes^17^ (Si_3_P_3_R_3_ and Si_3_As_3_R_3_, respectively), and the germanium analog (germanazene^18^; Ge_3_N_3_R_3_), where R, R’, and R” denote substituents. The structural diversity of these inorganic benzenes allows us to develop novel materials with unique photophysical properties that are unattainable in conventional carbon-based aromatic systems.

The doubly degenerate highest occupied molecular orbital (HOMO) and lowest unoccupied molecular orbital (LUMO) of borazine are predominantly localized on the N and B atoms, respectively (Fig. 1b). Thus, the HOMO→LUMO excitation of borazine can be viewed as the short-range charge-transfer^19^ (SRCT) electronic excitation between the N and B atoms. For the inorganic-type benzenes listed above, S_1_ can be energetically lower than T_1_ as confirmed below, leading to a unique electronic property called the inverted singlet-triplet (iST or INVEST): the S_1_-T_1_ energy difference Δ*E*_ST_ is negative. Here, Δ*E*_ST_ = *E*(S_1_) − *E*(T_1_); *E*(S_1_) and *E*(T_1_) are the S_1_ and T_1_ energies, respectively.

The presence of iST was originally suggested theoretically by Borden^20^, Kollmar, and Staemmler^21^ for cyclobutadiene in the 1970s. Experimental iST study was performed by Leupin and Wirz for cycl[3.3.3]azine^22^ in 1980. After a long silence, interest in iST was reignited by a pivotal theoretical study by de Silva in 2019, which clearly demonstrated the possibility of negative singlet-triplet gap by considering double excitations^23^. Experimental observations have also emerged for azaphenalene derivatives, following the work by Domcke’s group in 2019^24–28^. These works have further driven theoretical efforts toward accurate estimation of singlet-triplet energy gaps, where high-order excitations play an important role^29–32^. Because iST allows a thermodynamically favorable T_1_ → S_1_ transition, iST materials can show efficient reverse intersystem crossing (RISC) from T_1_ to S_1_ and are promising as fluorescent materials for organic light-emitting diodes (OLEDs). However, to date, the rate constants for S_1_→S_0_ fluorescence (*k*_F_) are not large enough, on the order of 10^6^ s^−1^. In this study, we theoretically investigate the potential iST character of inorganic benzenes. Our quantum chemical calculations indicate that borazine derivatives are promising candidates for electroluminescent UV emitters exhibiting both negative Δ*E*_ST_ and *k*_F_ of 10^7^–10^8^ s^−1^ (see Table 1 below). These *k*_F_ values, one to two orders of magnitude higher than those of previously reported iST molecules, represent a significant advancement in emitter performance. Borthiins and boroselenol are also found to show negative Δ*E*_ST_ and are candidates for core units to develop wide gap iST materials for OLEDs. The gallium-nitride (GaN) analog of benzene (Ga_3_N_3_H_6_) is also predicted to show iST character with a wide optical gap.

**Table 1:** Point groups and Δ*E*_ST_, *E*(S_1_), *E*(T_1_), *μ*_F_, *f*, *k*_F_, *k*_ISC_, and *k*_RISC_ of inorganic benzenes calculated with the SCS-CC2/def2-TZVP//PBE0/6-31G(d) method

	Molecular formula	Point group	Δ*E*_ST_ (meV)	*E*(S_1_) (eV)	*E*(T_1_) (eV)	*μ*_F_ (au)	*f*	*k*_F_ (s^−1^)	*k*_ISC_ (s^−1^)	*k*_RISC_ (s^−1^)
**1**: ben-BN	B_3_N_3_H_6_	*D*_*3h*_	**−295**	6.83	7.13	0.0000	0.0	0.0	4.5 × 10^2^	**1.5 × 10**^**7**^
**2a**: naph-BN	B_5_N_5_H_8_	*C*_*2v*_	**−95**	6.64	6.73	0.1502	3.7 × 10^−3^	7.0 × 10^6^	2.4 × 10^6^	**3.4 × 10**^**7**^
**2b**: anth-BN	B_7_N_7_H_10_	*C*_*2v*_	**−21**	6.63	6.65	0.4159	2.8 × 10^−2^	**5.4 × 10**^**7**^	0.0	0.0
**2c**: phenan-BN	B_7_N_7_H_10_	*C*_*s*_	**−127**	6.57	6.70	0.2990	1.4 × 10^−2^	**2.7 × 10**^**7**^	2.8 × 10^5^	**1.9 × 10**^**7**^
**2d**: tetra-BN	B_9_N_9_H_12_	*C*_*2v*_	**−51**	6.62	6.67	0.7023	8.0 × 10^−2^	**1.5 × 10**^**8**^	0.0	0.0
**2e**: cryc-BN	B_9_N_9_H_12_	*C*_*s*_	**−59**	6.56	6.62	0.1300	2.7 × 10^−3^	5.1 × 10^6^	4.7 × 10^6^	**2.3 × 10**^**7**^
**2f**: pyr-BN	B_8_N_8_H_10_	*C*_*2v*_	**−221**	6.54	6.76	0.0562	5.1 × 10^−4^	9.4 × 10^5^	2.5 × 10^3^	**8.0 × 10**^**6**^
**2g**: phenal-NB	B_7_N_6_H_9_	*D*_*3h*_	**−260**	6.59	6.85	0.0000	0.0	0.0	5.8 × 10^2^	**5.4 × 10**^**6**^
**2h**: phenal-BN	B_6_N_7_H_9_	*D*_*3h*_	**−128**	6.54	6.67	0.0000	0.0	0.0	0.0	0.0
**2i**: olymp-NB	B_10_N_9_H_11_	*C*_*2v*_	**−243**	6.54	6.78	0.0284	1.3 × 10^−4^	2.4 × 10^5^	0.0	0.0
**2j**: olymp-BN	B_9_N_10_H_11_	*C*_*2v*_	**−97**	6.53	6.63	0.2545	1.0 × 10^−2^	**1.9 × 10**^**7**^	0.0	0.0
**2k**: bis-BN	B_14_N_14_H_14_	*C*_*2v*_	**−57**	6.54	6.59	0.2110	7.1 × 10^−3^	**1.3 × 10**^**7**^	0.0	0.0
**3**: (6, 6)NB-BN	B_24_N_24_H_24_	*S*_*6*_	**−141**	6.26	6.40	0.0000	0.0	0.0	6.1 × 10^3^	**1.4 × 10**^**6**^
**4e**: [6]CPP-BN	B_18_N_18_H_24_	*S*_*6*_	**−145**	6.26	6.40	0.0000	0.0	0.0	6.7 × 10^2^	1.2 × 10^5^
**5**: [6]CMP-BN	B_18_N_18_H_24_	*C*_*3v*_	**−186**	6.45	6.64	0.0000	0.0	0.0	1.4 × 10^2^	7.6 × 10^4^
**6e**: [6]PP-BN	B_18_N_18_H_26_	*C*_*2*_	**−77**	6.58	6.66	0.0458	3.4 × 10^−4^	6.4 × 10^5^	3.1 × 10^2^	1.5 × 10^3^
**7f**: [6]MP-BN	B_18_N_18_H_26_	*C*_*1*_	**−214**	6.49	6.70	0.0772	9.5 × 10^−4^	1.7 × 10^6^	1.0 × 10^3^	**1.4 × 10**^**6**^
**8a**: B_12_N_12_-fullerene	B_12_N_12_	*T*_*h*_	**−134**	6.47	6.60	0.0000	0.0	0.0	6.6 × 10^1^	3.8 × 10^3^
**9**: ben-BS	B_3_S_3_H_3_	*D*_*3h*_	**−109**	5.02	5.13	0.0000	0.0	0.0	3.7 × 10^6^	**1.8 × 10**^**8**^
**10**: ben-BSe	B_3_Se_3_H_3_	*D*_*3h*_	**−160**	4.42	4.58	0.0000	0.0	0.0	1.2 × 10^7^	**2.1 × 10**^**9**^
**11**: ben-BO	B_3_O_3_H_3_	*D*_*3h*_	326	8.11	7.79	0.0000	0.0	0.0	6.3 × 10^10^	1.6 × 10^5^
**12**: ben-GaN	Ga_3_N_3_H_6_	*D*_*3h*_	**−230**	5.52	5.75	0.0000	0.0	0.0	0.0	0.0
**13**: prism-GaN	Ga_3_N_3_H_6_	*C*_*s*_	507	4.11	3.61	0.7097	5.1 × 10^−2^	**3.7 × 10**^**7**^	2.0 × 10^8^	2.5 × 10^−1^

## Results

### Inorganic benzenes

**Fig. 2:**
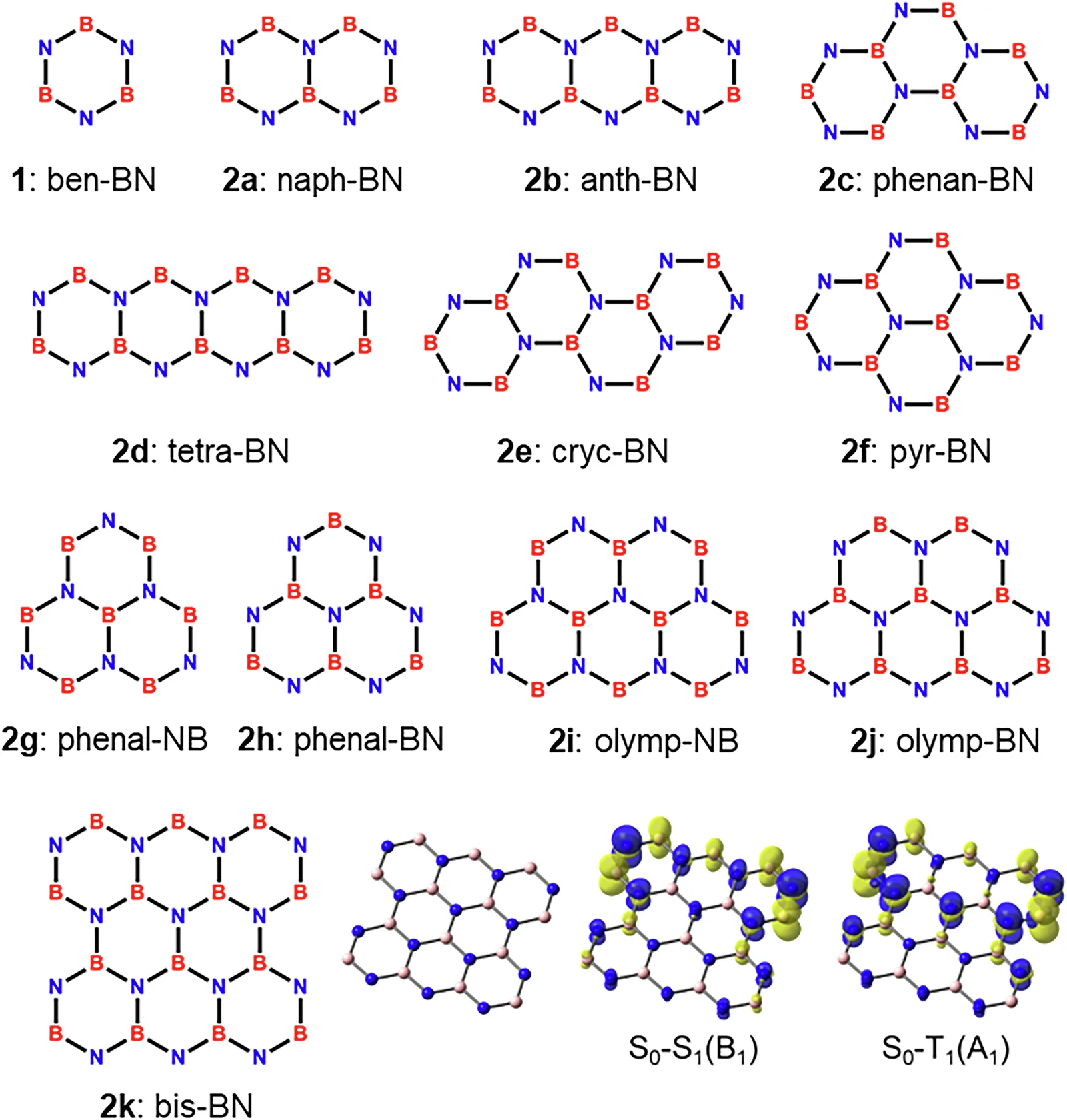
Schematic structures of borazine and one- and two-dimensional fused borazines. Hydrogen atoms are omitted for clarity. **1:** borazine (**ben-BN**), **2a:** naphthalene-type BN (**naph-BN**), **2b:** anthracene-type BN (**anth-BN**), **2c:** phenanthrene-type BN (**phenan-BN**), **2d: tetracene**-type BN (**tetra-BN**), **2e:** crycene-type BN (**cryc-BN**), **2f:** pyrene-type BN (**pyr-BN**), **2g:** phenalene-type nitride boron (**phenal-NB**), **2h:** phenalene-type BN (**phenal-BN**), **2i:** olympicene-type NB (**olymp-NB**), **2j:** olympicene-type BN (**olymp-BN**), and **2k:** bisanthrene-type BN (**bis-BN**). Electron-density difference (Δ*ρ*) maps arising from the S_0_→S_1_(B_1_) and S_0_→T_1_(A_1_) excitations of **2k: bis-BN** are also shown. Blue regions show negative Δ*ρ* (where the electron density decreases). Yellow regions show positive Δ*ρ* (where the electron density increases).

Figures 1 and 2 show the structures and abbreviated names of the borazine family investigated in this study: borazine (**1: ben-BN**) and one- and two-dimensional (1D and 2D) fused borazines (**2a: naph-BN** to **2k: bis-BN**). In Fig. 2, the H atoms are omitted for clarity; the optimized structures with H atoms are shown in Supplementary Information (Supplementary Fig. 1). **2k: bis-BN** can be viewed as a model of hBN. Δ*E*_ST_, *E*(S_1_), *E*(T_1_), the S_0_-S_1_ transition dipole moment (*μ*_F_), the S_0_-S_1_ oscillator strength (*f*), and the rate constants *k*_F_, the intersystem crossing rate constants (*k*_ISC_), and the reverse intersystem crossing rate constants (*k*_RISC_) of these compounds calculated with spin-component scaled second-order approximate coupled-cluster (SCS-CC2) are listed in Table 1. The detailed formulas used to evaluate the rate constants are presented in Supplementary Methods. These values were calculated at the S_0_-optimized geometry at the PBE0/6-31G(d) level of theory. The corresponding data calculated with the second-order algebraic diagrammatic construction (ADC(2)) are listed in Supplementary Table 1, and the SCS-CC2 results at the geometries optimized at the MP2/6-31 G(d) level are listed in Supplementary Table 4. See the “Methods” section for the calculation details. Importantly, all of **1**: **ben-BN** to **2k: bis-BN** show negative Δ*E*_ST_ (<−21 meV) and large *E*(S_1_) (>6.53 eV), suggesting that these compounds are promising as wide-gap core materials with iST character. It would be possible to develop materials with preferred emission wavelengths through appropriate structural modifications. It is instructive to compare the present result for **1: ben-BN** with the previous report by Sobolewski and Domcke^33^, in which calculated values of Δ*E*_ST_ for **1: ben-BN** were compared among several wavefunction-based methods. Their ADC(2)/cc-pVTZ result (Δ*E*_ST_ = −195 meV) is well reproduced by our ADC(2)/def2-TZVP calculation (Δ*E*_ST_ = −193 meV), where the slight deviation is attributed to the different optimization methods used for the reference geometry. They also reported that CC2/cc-pVTZ results in a less negative value (Δ*E*_ST_ = −90 meV), and EOM-CCSD/cc-pVTZ predicts a positive value (Δ*E*_ST_ = +19 meV). Compared to their results and our ADC(2)/def2-TZVP result, our SCS-CC2/def2-TZVP result (Δ*E*_ST_ = −295 meV) is considerably more negative. At this stage, it is difficult to judge which value is closest to the “exact” result due to the lack of experimental benchmarks. For the calculations, more reliable reference values obtained with higher-order correlation treatments may be required. Sobolewski and Domcke also reported that Δ*E*_ST_ shifts positively (but still remains negative for the ADC(2) and CC2 methods) when cc-pVDZ basis set is used instead of cc-pVTZ. In view of this report, we also performed calculations with cc-pVDZ basis set for all the molecules considered in Table 1 (Supplementary Tables 6–8), indicating that the difference in the Δ*E*_ST_ values from the corresponding cc-pVTZ results does not substantially affect our discussion; the only one exception is **6e: [6]PP-BN**, for which the sign of Δ*E*_ST_ changes.

To confirm the SRCT character of **1**: **ben-BN**, we calculated electron-density difference (Δ*ρ*) maps arising from the S_0_→S_1_ and S_0_→T_1_ excitations (Fig. 1c). The Δ*ρ* maps were calculated by subtracting the total electron density of S_0_ from that of S_1_ or T_1_, using the S_0_ structure. Figure 1c shows the Δ*ρ* maps for the S_0_→S_1_(B_1_), S_0_→T_1_(A_1_), and S_0_→T_1_(B_1_) excitations of **1: ben-BN**, where T_1_(A_1_) and T_1_(B_1_) represent doubly degenerate T_1_ of different symmetries, A_1_ and B_1_, respectively. Blue regions show negative Δ*ρ*, where the electron density decreases, and yellow regions show positive Δ*ρ*, where the electron density increases. The negative Δ*ρ* is strongly localized on the N atoms, whereas the positive Δ*ρ* is strongly localized on the B atoms, suggesting that a short-range electron transfer from the N to B atoms occurs upon the excitations. In addition, Δ*ρ* shows spatially alternating negative and positive regions, which are characteristic for B- and N-containing molecules with small positive Δ*E*_ST_^34–36^ and heptazine derivatives with negative Δ*E*_ST_^37^. Fig. 2 shows calculated Δ*ρ* maps for the S_0_→S_1_(B_1_) and S_0_→T_1_(A_1_) excitations of **2k: bis-BN**. Like **1**: **ben-BN,** **2k: bis-BN** has spatially alternating negative and positive regions, indicating SRCT character.

From Eqs. (S1) and (S2) in the Supplementary Information, a large *μ*_F_ is desirable to increase *f* and *k*_F_, which enhances the luminescence efficiency. From Table 1, it is found that *f* is large for 1D structures, especially when the fused ring becomes longer (**1: ben-BN**→**2a: naph-BN**→**2b: anth-BN**→**2d: tetra-BN**), whereas it vanishes when the molecular structure is 3-fold triangular symmetric (**1**: **ben-BN,** **2g**: **phenal-NB**, and **2h**: **phenal-BN**). **2d**: **tetra-BN** shows the largest *k*_F_ of 1.5 × 10^8^ s^−1^ among the molecules in Fig. 2. These results suggest that breaking the *C*_3_ symmetry and extending the molecular framework in one direction are key factors for increasing *μ*_F_, *f*, and the resulting *k*_F_. Table 1 also shows *k*_ISC_ and *k*_RISC_ between S_1_ and T_1_. It has been reported that activation barriers can exist in RISC processes even when Δ*E*_ST_ is negative^38,39^. Further, quantitative estimation of rate constants requires nuclear quantum effects to be taken into account. We therefore employed analytical expressions for *k*_ISC_ and *k*_RISC_ derived via Fermi’s golden rule with explicit consideration of nuclear quantum effects^40,41^, as summarized in the Supplementary Method. For molecules with large *k*_F_ (>10^7^ s^−1^), *k*_ISC_ was smaller by orders of magnitude, except for **13: prism-GaN**. This result suggests that ISC is unlikely to compete with fluorescence. The calculated *k*_RISC_ values indicate that **1: ben-BN,** **2a: naph-BN,** **2c: phenan-BN,** **2e: cryc-BN,** **2f: pyr-BN,** **2**g: phenal-BN, **3: (6, 6)NB-BN,** **7f: [6]MP-BN,** **9: ben-BS**, and **10: ben-BSe** have RISC rate constants over 10^6^ s^−1^, which are comparable to typical values reported for known TADF emitters. In comparison with the SCS-CC2 results (Table 1), the ADC(2) results (Supplementary Table 1) tend to yield less negative $\Delta {E}_{{{\rm{ST}}}}$ values, while the sign remains unchanged for majority of the molecules.

To evaluate the performance as emitters in more detail, we also performed calculations at S_1_-optimized geometries. In addition to *k*_F_, *k*_ISC_, and *k*_RISC_, the nonradiative decay rate constants (*k*_NR_) were also calculated. The results (Supplementary Table 5) indicate that *k*_NR_ and *k*_ISC_ are smaller than *k*_F_ for molecules with large *k*_F_ (>10^8^ s^−1^), suggesting that fluorescence is the dominant relaxation pathway from S_1_ in these molecules.

### Ring- and fullerene-type borazines

**Fig. 3:**
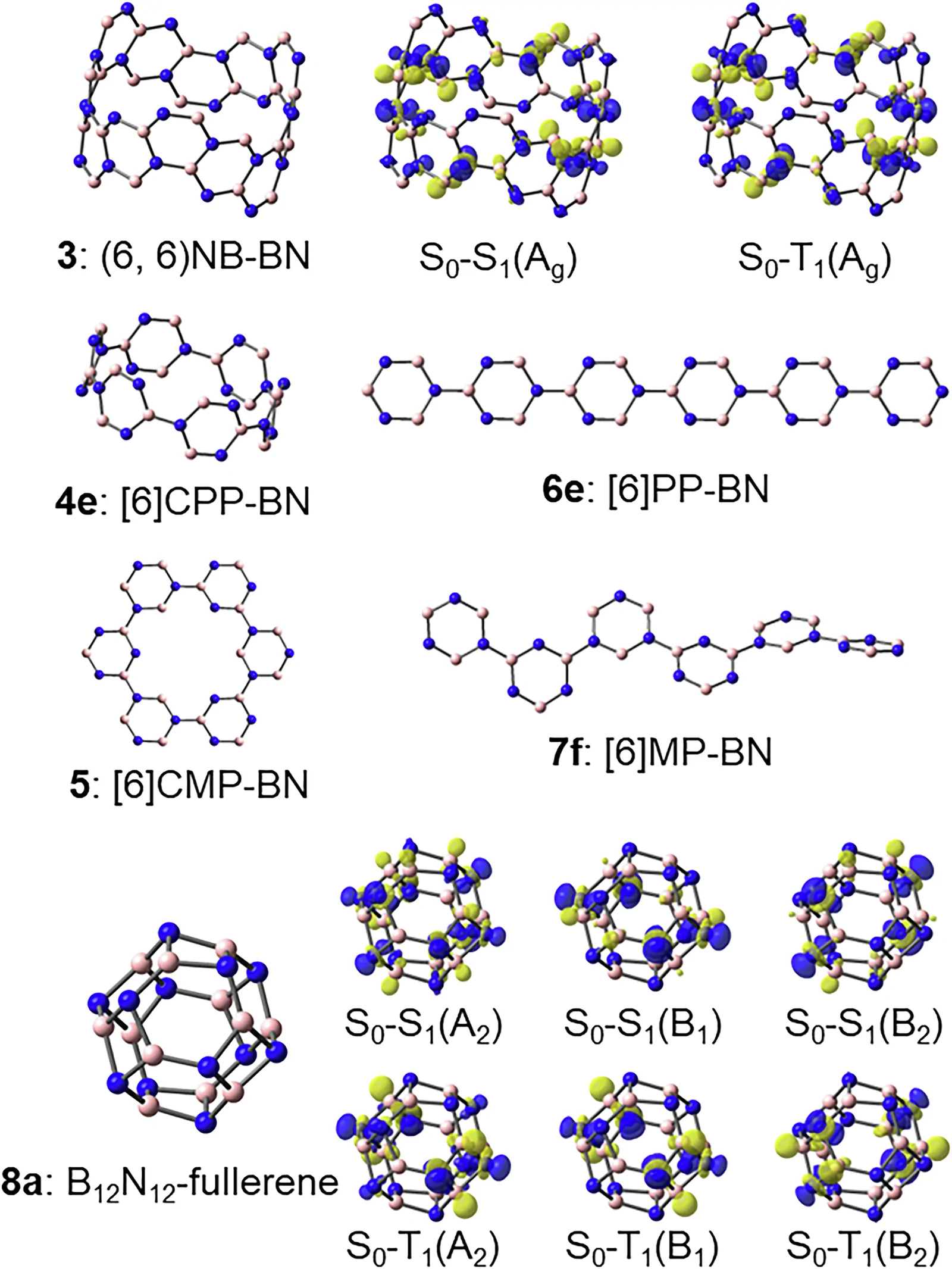
DFT-optimized structures of ring-type borazines (3, 4e, and 5), their one-dimensional counterparts (6e and 7f), and a fullerene-type BN compound (8a) at the PBE0/6-31G(d) level of theory. **3:** (6, 6)carbon nanobelt-type BN (**(6, 6)NB-BN**), **4e:** [6]cycloparaphenylene-type BN (**[6]CPP-BN**), **6e:** [6]paraphenylene-type BN (**[6]PP-BN**), **5:** [6]cyclometaphenylene-type BN (**[6]CMP-BN**), **7f:** [6]metaphenylene-type BN (**[6]MP-BN**), **8a:** C_24_-fullerene-type BN (**B**_**12**_**N**_**12**_**-fullerene**). Hydrogen atoms are omitted for clarity except for **8a: B**_**12**_**N**_**12**_**-fullerene**. Electron-density difference (Δ*ρ*) maps arising from the S_0_→S_1_(A_g_) and S_0_→T_1_(A_g_) excitations of **3: (6, 6)NB-BN** and the S_0_→S_1_(A_2_), S_0_→S_1_(B_1_), S_0_→S_1_(B_2_), S_0_→T_1_(A_2_), S_0_→T_1_(B_1_), and S_0_→T_1_(B_2_) excitations of **8a: B**_**12**_**N**_**12**_**-fullerene** are also shown. Blue regions show negative Δ*ρ* (where the electron density decreases). Yellow regions show positive Δ*ρ* (where the electron density increases). The twisted structure is most stable for **7f: [6]MP-BN**, due to the steric hindrance among H atoms.

Figure 3 shows cyclic borazines, which are parts of BNNTs. The Δ*E*_ST_, *E*(S_1_), *μ*_F_, *f*, and *k*_F_ values are also shown in Table 1. **3: (6, 6)NB-BN** is a subunit of a BNNT, consisting of 12 fused borazine rings. The structure of **3: (6, 6)NB-BN** was designed with reference to a carbon nanobelt structure in ref. ^42^. (a subunit of a (6, 6)-carbon nanotube) and then, optimized at the PBE0/6-31 G(d) level of theory. **3: (6, 6)NB-BN** shows a negative Δ*E*_ST_ of −141 meV and a large *E*(S_1_) of 6.26 eV (Table 1). Figure 3 also shows the calculated Δ*ρ* maps for the S_0_→S_1_(A_g_) and S_0_→T_1_(A_g_) excitations of **3: (6, 6)NB-BN**. Similarly to the above cases, the negative Δ*ρ* (blue regions) is strongly localized on the N atoms, whereas the positive Δ*ρ* (yellow regions) is strongly localized on the B atoms, suggesting that S_1_ and T_1_ of **3: (6, 6)NB-BN** also have pure SRCT character. The *f* value of **3: (6, 6)NB-BN** vanishes because of the symmetric ring structure, which would make **3: (6, 6)NB-BN** non-emissive. **4e: [6]CPP-BN** is another subunit of the BNNT; the diameter of **4e: [6]CPP-BN** (8.36 Å) is slightly larger than that of **3: (6, 6)NB-BN** (8.32 Å). **4e: [6]CPP-BN** consists of six borazine rings and is the borazine analog of [6]cycloparaphenylene ([6]CPP)^43^, a subunit of a (6,6)-carbon nanotube. The Δ*E*_ST_, *E*(S_1_), *μ*_F_, *f*, and *k*_F_ of **4e: [6]CPP-BN** are similar to those of **3: (6, 6)NB-BN**, despite the difference in conjugated state. **5**: **[6]CMP-BN** also consists of six borazine rings but forms a different ring configuration; the borazine analog of [6]cyclometaphenylene^44^. The Δ*E*_ST_ of **5: [6]CMP-BN** (−186 meV) is smaller than those of **3: (6, 6)NB-BN** and **4e: [6]CPP-BN**. Similarly to **3: (6, 6)NB-BN** and **4e: [6]CPP-BN**, the symmetric ring structure results in *f* = 0. To understand the influence of the ring structures on the *f* values, we compared the *f* values of **4e: [6]CPP-BN** and **5: [6]CMP-BN** with those of their 1D counterparts, **6e: [6]PP-BN** and **7f: [6]MP-BN**. Unlike **4e: [6]CPP-BN** and **5: [6]CMP-BN,** **6e: [6]PP-BN** and **7**f: [6]MP-BN have non-zero *f* values, leading to *k*_F_ of the order of 10^5^–10^6^ s^−1^, suggesting that the symmetric ring formation largely decreases *f* value. The calculated Δ*E*_ST_, *E*(S_1_), *μ*_F_, *f*, and *k*_F_ values for 5–12-membered borazine rings and their 1D counterparts are summarized in Supplementary Information (molecules **4d: [5]CPP-BN** - **4k**: **[12]CPP-BN** in Supplementary Fig. 2, molecules **6a: [2]PP-BN** - **6k: [12]PP-BN** in Supplementary Fig. 3, and Supplementary Table 2). The ring molecules (**4d: [5]CPP-BN** - **4k**: **[12]CPP-BN**) have zero or nearly zero *f* values, whereas the 1D counterparts (**6d: [5]PP-BN** - **6h: [9]PP-BN**) have non-zero *f* values with *k*_F_ of 10^4^–10^6^ s^−1^. Linear metaphenylene-type molecules (**7a: [3]MP-BN** - **7f: [6]MP-BN**) also have non-zero *f* values with *k*_F_ of 10^5^–10^7^ s^−1^ (Supplementary Fig. 4 and Supplementary Table 2).

Borazine analogs of fullerenes have been experimentally observed^45–47^. B_12_N_12_-fullerene is a borazine analog of C_24_ fullerene and consists of the four- and six-membered rings (note that **8a: B**_**12**_**N**_**12**_**-fullerene** and all fullerene-type compounds have no H atoms). **8a: B**_**12**_**N**_**12**_**-fullerene** shows a negative Δ*E*_ST_ of −134 meV, which is comparable to those of **3: (6, 6)NB-BN** and **4e: [6]CPP-BN** (−141 and −145 meV, respectively). S_1_ and T_1_ of **8a: B**_**12**_**N**_**12**_**-fullerene** are triply degenerate (denoted as A_2_, B_1_, and B_2_) owing to its highly symmetric structure. Figure 3 shows Δ*ρ* maps for the S_0_→S_1_ and S_0_→T_1_ excitations of **8a: B**_**12**_**N**_**12**_**-fullerene**. The negative and positive Δ*ρ* distributions suggest that S_1_ and T_1_ of **8a: B**_**12**_**N**_**12**_**-fullerene** show pure SRCT character. Like the ring borazines of **3: (6, 6)NB-BN,** **4e: [6]CPP-BN**, and **5: [6]CMP-BN,** **8a: B**_**12**_**N**_**12**_**-fullerene** has zero *f* value and is expected to be non-emissive. The calculated results for borazine analogs of C_48_ and C_72_ fullerenes are shown in Supplementary Information (molecules **8b: B**_**24**_**N**_**24**_**-fullerene** and **8c: B**_**36**_**N**_**36**_**-fullerene** in Supplementary Fig. 5 and Supplementary Table 2). Although **8b: B**_**24**_**N**_**24**_**-fullerene** and **8c: B**_**36**_**N**_**36**_**-fullerene** show positive Δ*E*_ST_, the Δ*E*_ST_ values (~*k*_B_*T* at room temperature) are sufficiently small to induce rapid T_1_→S_1_ transition.

### Inorganic-type benzenes other than borazine

**Fig. 4:**
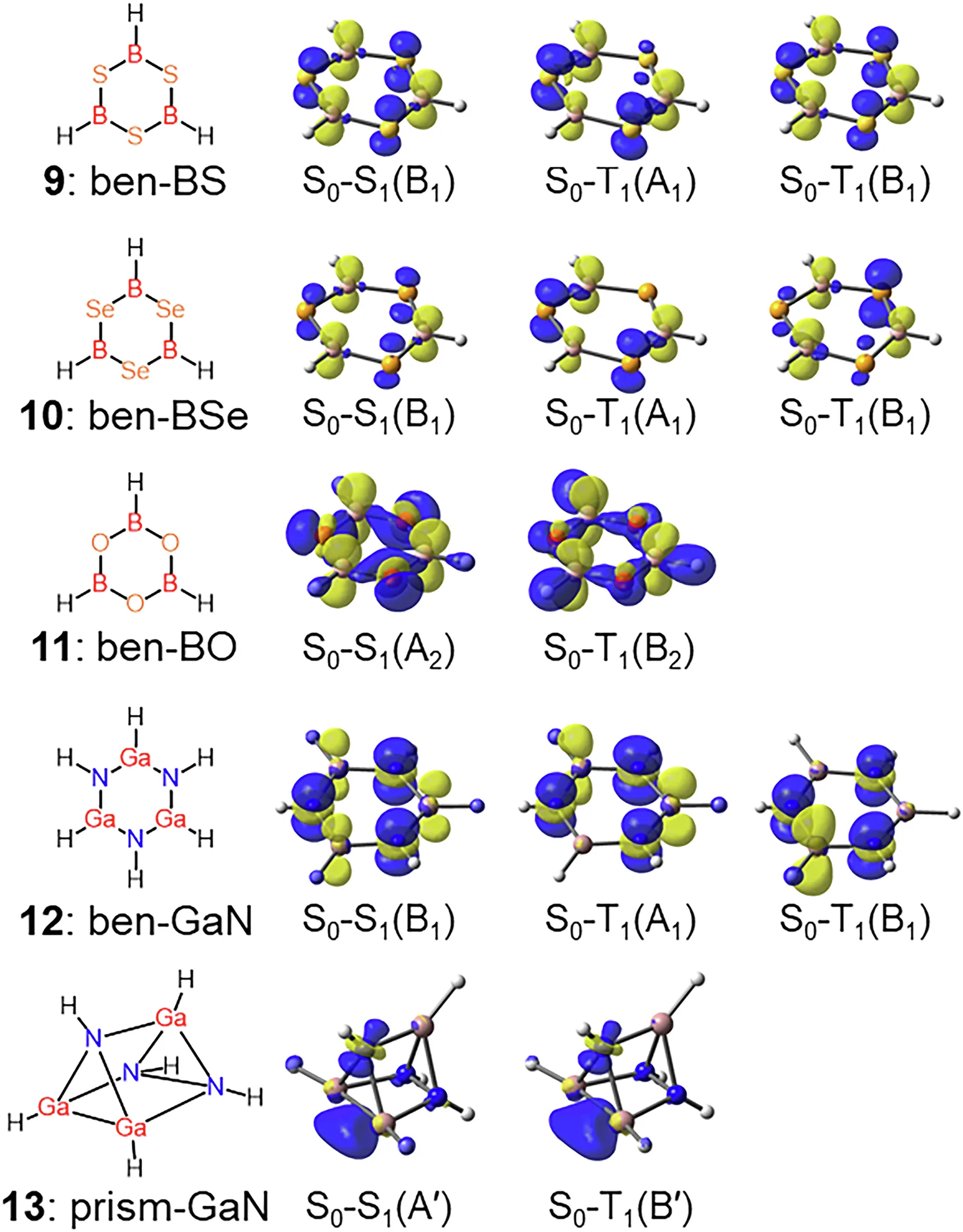
Inorganic-type benzenes other than borazine. **9:** borthiin (benzene-type boron sulfide; **ben-BS**), **10:** boroselenol (benzene-type boron selenide; **ben-BSe**), **11:** boroxine (benzene-type BO; **ben-BO**), **12:** benzene-type gallium nitride (**ben-GaN**), **13:** prismane-type gallium nitride (**prism-GaN**). Electron-**density** difference (Δ*ρ*) maps arising from the S_0_→S_1_ and S_0_→T_1_ excitations of **9: ben-BS** - **13**: **prism-GaN** are also shown. Blue regions show negative Δ*ρ*, where the electron density decreases. Yellow regions show positive Δ*ρ*, where the electron density increases.

Finally, we investigate the potential of inorganic-type benzenes other than borazine-based compounds as wide-gap iST materials (**9: ben-BS** - **13: prism-GaN** in Fig. 4 and **14: ben-B**_**4**_**N**_**2**_ - **23: ring-Ge**_**3**_**N**_**3**_ in Supplementary Fig. 6). Derivatives of **9: ben-BS** - **11: ben-BO** and **14: ben-B**_**4**_**N**_**2**_ - **23: ring-Ge**_**3**_**N**_**3**_ have been experimentally synthesized^8–18^, whereas **12**: **ben-GaN** and **13: prism-GaN** have been only theoretically predicted^48,49^. Substituents required for organic synthesis are replaced with H atoms. Borthiin (**9: ben-BS**) and boroselenol (**10: ben-BSe**) show negative Δ*E*_ST_ (<−100 meV), whereas the oxygen (O) analog (boroxine, **11: ben-BO**) shows a large positive Δ*E*_ST_ of 326 meV (Table 1). To understand the difference in the Δ*E*_ST_ values of **9: ben-BS** - **11: ben-BO**, we compared the Δ*ρ* maps for the S_0_→S_1_ and S_0_→T_1_ excitations of **9: ben-BS** - **11: ben-BO** (Fig. 4). For **9: ben-BS** and **10: ben-BSe**, the negative Δ*ρ* is strongly localized on the S and Se atoms, whereas the positive Δ*ρ* is strongly localized on the B atoms. The Δ*ρ* patterns are similar to those observed for borazine (**1: ben-BN**, Fig. 1). Meanwhile, for **11: ben-BO**, the negative Δ*ρ* is largely distributed on the B-O and B-H bonds, whereas the positive Δ*ρ* is strongly localized on B atoms, which breaks down SRCT character. This pattern of Δ*ρ* suggests the significant overlap of the MOs, which may yield large exchange interaction that dominates over the spin polarization (double excitation) effect, leading to the large positive Δ*E*_ST_ of 326 meV. GaN is a wide-bandgap semiconductor and is widely used for blue and white light-emitting diodes^50–53^. Hence, although the GaN analog of benzene (Ga_3_N_3_H_6_) has not been synthesized yet, it is worth investigating its potential iST property. Two structures have been theoretically proposed for Ga_3_N_3_H_6_: a planar benzene-type structure^48^ (**12: ben-GaN**), where Ga and N atoms are arranged alternatively, and a prismane-type structure^49^ (**13: prism-GaN**), where two Ga and N atoms are adjacent to each other. Calculated Δ*ρ* maps for the S_0_→S_1_ and S_0_→T_1_ excitations of **12: ben-GaN** show SRCT with spatially alternating negative and positive regions, whereas those of **13: prism-GaN** have large distributions on the Ga-Ga bonds. Consequently, **12: ben-GaN** shows negative Δ*E*_ST_ of −230 meV, whereas **13: prism-GaN** shows a large positive Δ*E*_ST_ of 507 meV (Table 1). Thus, like borazine, the GaN analog of benzene (**12: ben-GaN**) is predicted to show iST owing to its SRCT character in the S_0_→T_1_ and S_0_→S_1_ excitations. For other inorganic-type molecules, **14: ben-B**_**4**_**N**_**2**_, **15: ben-BP,** **16: ben-NP,** **17: ring-Ga**_**3**_**P**_**3**_, **18: ben-AlN,** **19: ring-Al**_**3**_**P**_**3**_, **20: ring-Al**_**3**_**As**_**3**_, **21: ring-(Si(N**_**2**_**CH**_**3**_**))**_**3**_**P**_**3**_, **22: ring-(Si(N**_**2**_**CH**_**3**_**))**_**3**_**As**_**3**_, and **23: ring-Ge**_**3**_**N**_**3**_ (Supplementary Fig. 6), Δ*E*_ST_ were calculated to be positive (>100 meV, Supplementary Table 3). Among the 15 inorganic benzenes (**9: ben-BS** – **23: ring-Ge**_**3**_**N**_**3**_) examined in Fig. 4 and Supplementary Fig. 6, only **9: ben-BS,** **10: ben-BSe**, and **12: ben-GaN** were expected to show iST.

## Discussion

We theoretically investigated the potential iST character of inorganic-type benzenes, mainly focusing on borazines. 2D-sheet, ring, and fullerene-type borazines showed the SRCT from N to B atoms in S_0_→S_1_ and S_0_→T_1_ excitations, which leads to the large inverted S_1_ and T_1_ energies. Other than borazines, ben-BS (B_3_S_3_H_3_, borthiin), ben-BSe (B_3_Se_3_H_3_, boroselenol), and the ben-GaN (Ga_3_N_3_H_6_) were found to show iST. These molecules have large S_1_ energies, leading to the development of promising electroluminescent UV emitters and wide-gap hosts with iST properties. Totally, we found 43 compounds with negative Δ*E*_ST_s. Among them, several compounds exhibit *k*_F_ of 10^7^–10^8 ^s^−1^, which will be expected as efficient emitters. According to the present results, a limited number of molecules simultaneously satisfy sufficiently large *k*_F_ and *k*_RISC_. Nevertheless, taking into account vibrational motion, exemplified by the Herzberg-Teller effect, might extend this compatibility to a larger number of molecules. In addition, by introducing various functional groups to these compounds, it is expected that excellent iST emitting materials with tunable emission wavelengths can be developed.

## Methods

### Geometry optimization

The ground-state (S_0_) geometries of the inorganic-type benzene materials were optimized at the PBE0/6-31G(d) level of theory, and their singlet and triplet excited states were calculated using the SCS-CC2 method with the def2-TZVP basis for the S_0_ geometries (the optimized geometries are listed in Supplementary Tables 9–65). The method is denoted as SCS-CC2/def2-TZVP//PBE0/6-31 G(d). For comparison, calculations were also performed for the MP2-optimized geometries, SCS-CC2/cc-pVTZ//MP2/6-31G(d) (Supplementary Table 4). Furthermore, calculations using the SCS-CC2/cc-pVTZ//TD-PBE0/6-31 G(d) method at the S_1_ geometries were carried out (Supplementary Table 5). The SCS-CC2 method considers double-electron excitations and has been shown to accurately predict Δ*E*_ST_ for molecules consisting of B and N backbones^34–36^. The S_0_- and S_1_-geometry optimizations were performed using the Gaussian 16 program package^54^.

### Calculation of electronic properties

Δ*E*_ST_, *E*(S_1_), *E*(T_1_), *μ*_F_, and *f* were calculated using the Turbomole program package^55^. *k*_F_, *k*_ISC_, and *k*_RISC_ were calculated using Eqs. (S1)–(S5) in the Supplementary Information. The ADC(2) method also considers double-electron excitations and has been used to compute Δ*E*_ST_ of iST molecules^56^. The results for ADC(2) calculations are summarized in the Supplementary Information (Supplementary Tables 1–3). Note that triple excitations were not considered in this study due to the high computational cost (it has been reported that inclusion of triple excitations tends to make Δ*E*_ST_ less negative^29,57^). The S_1_-T_1_ spin-orbit coupling (SOC) values were estimated using PySOC package^58^, based on the excited-state wavefunctions obtained at the TD-PBE0/6-31G(d) level of theory.

## Supplementary information

Transparent Peer Review file

Supplementary Information

## Data Availability

All relevant data are available from the corresponding author (kaji@scl.kyoto-u.ac.jp) upon request.
