# Supplementary material for: Inorganic benzenes with inverted singlet-triplet gaps
Source: Commun Chem. 2026 Jul 18;9:354. doi: 10.1038/s42004-026-02141-0 (PMC13748633; doi:10.1038/s42004-026-02141-0)
Supplement: Supplementary file 1 [file 42004_2026_2141_MOESM1_ESM.pdf]

Supplementary Information for

Inorganic benzenes with inverted singlet-triplet gaps

Katsuyuki Shizu[1], Kuraudo Ishihara[1], Hiroki Uratani[2], Hironori Kaji[1]*

[1]*Institute for Chemical Research, Kyoto University, Uji, Kyoto 611-0011, Japan*

[2]*Department of Molecular Engineering, Graduate School of Engineering, Kyoto University, Kyoto, 615-8510, Japan*

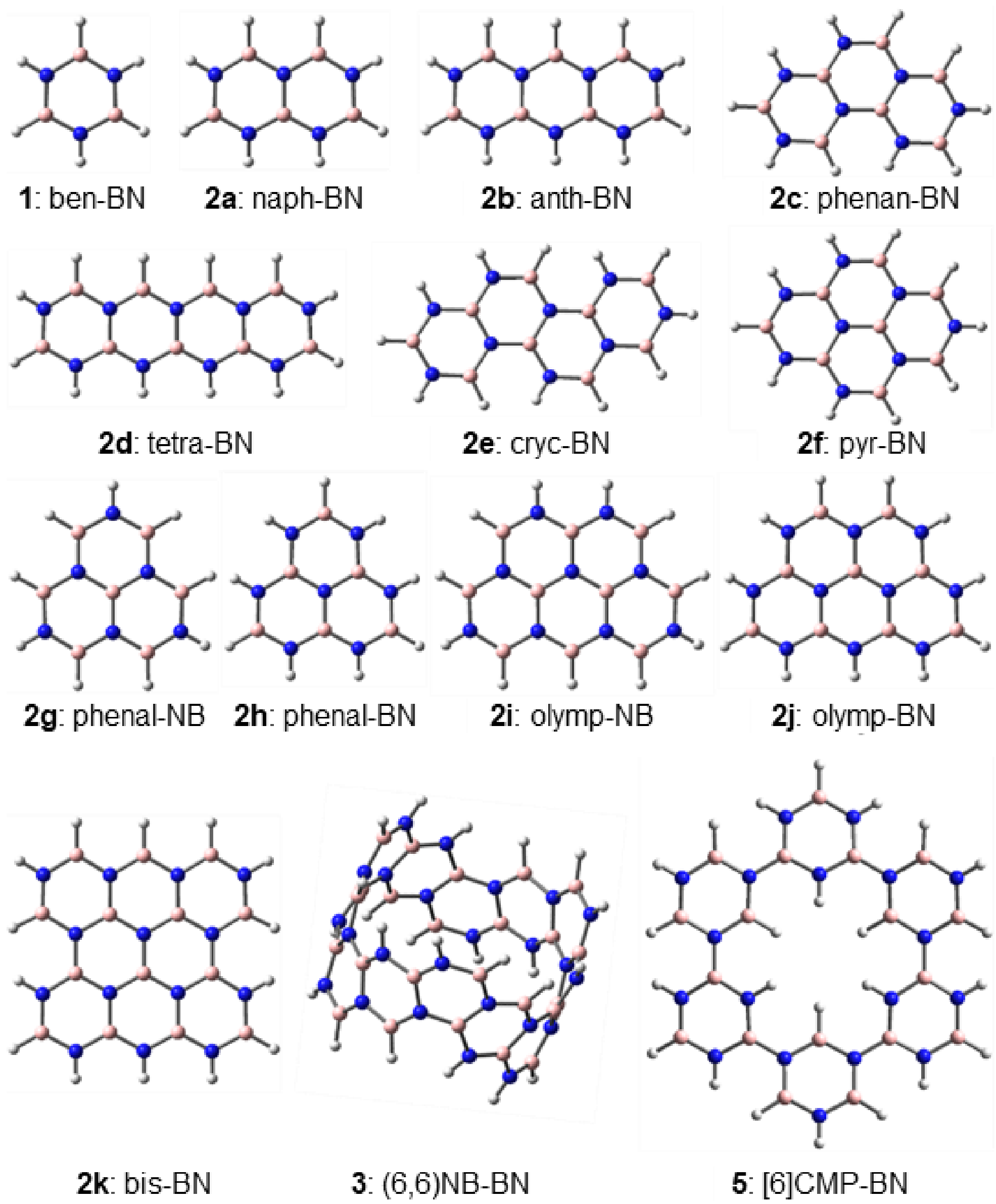

Supplementary Fig. 1 | Molecular structures of borazines **1: ben-BN - 2k: bis-BN**, **3: (6, 6)NB-BN**, and **5: [6]CMP-BN** optimized at the PBE0/6-31G(d) level of the theory.

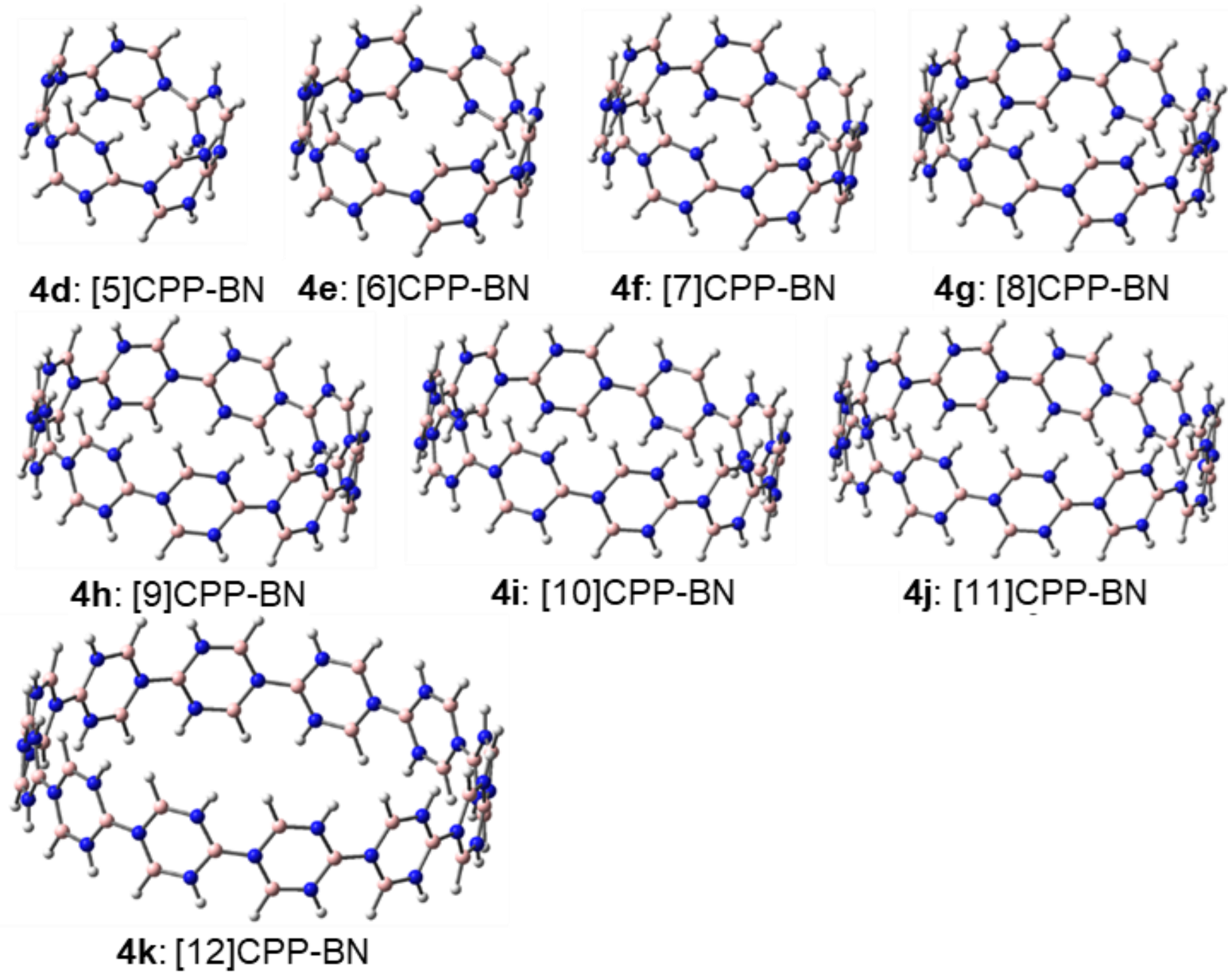

Supplementary Fig. 2 | Molecular structures of cycloparaphenylene-type borazine rings (**4d: [5]CPP-BN** - **4k: [12]CPP-BN**) optimized at the PBE0/6-31G(d) level of the theory.

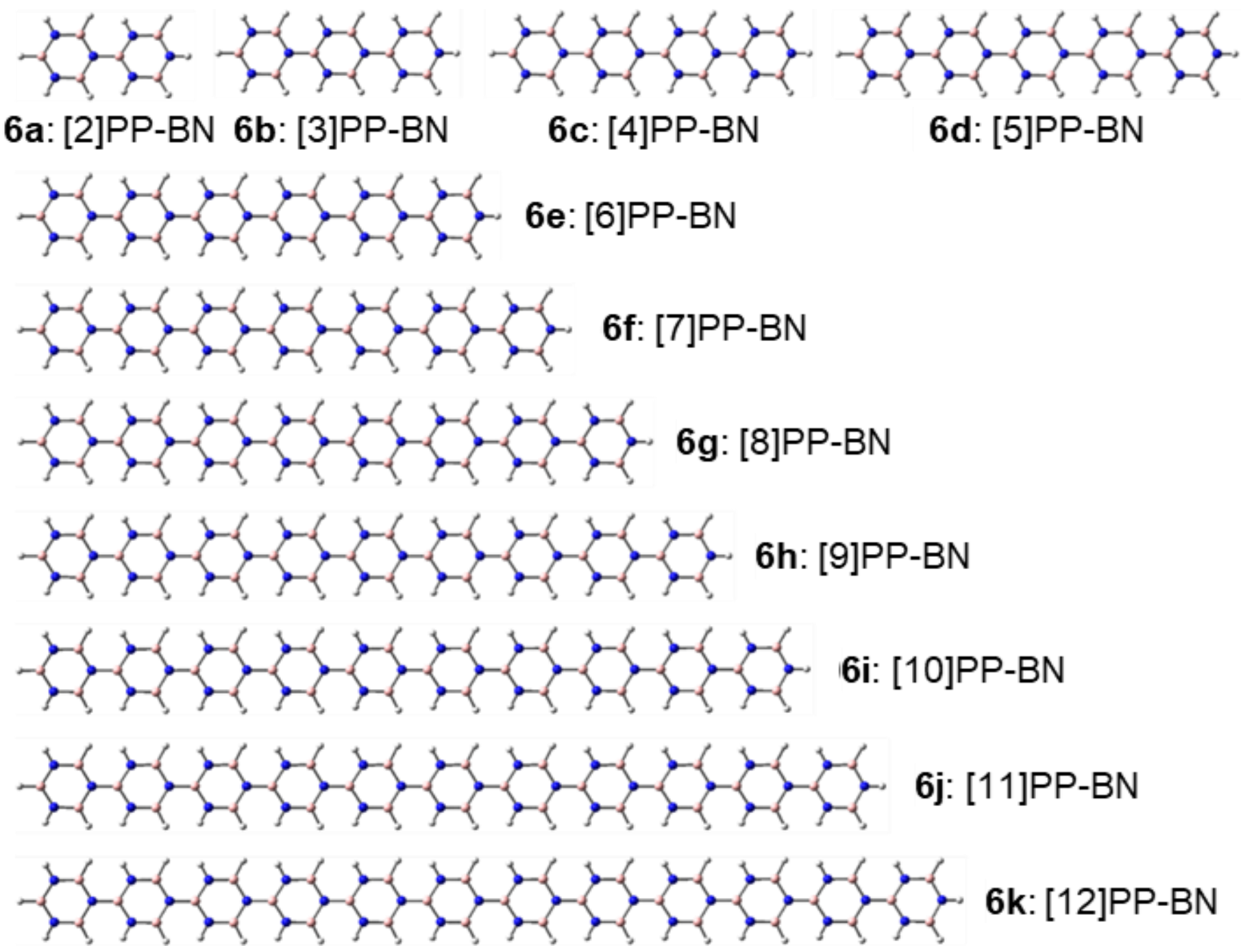

Supplementary Fig. 3 | Molecular structures of paraphenylene-type borazines (**6a: [2]PP-BN** - **6k: [12]PP-BN**) optimized at the PBE0/6-31G(d) level of the theory.

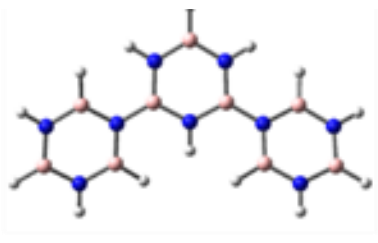
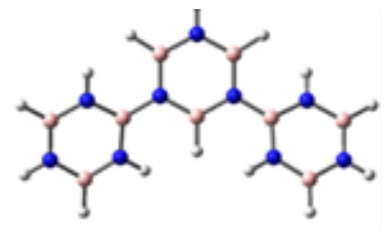
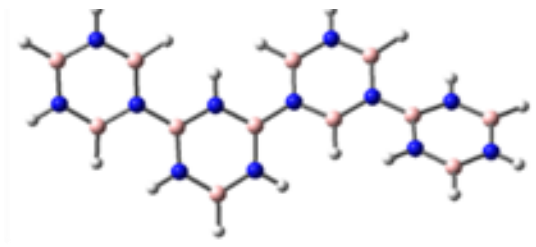

7a: [3]MP-BN **7b**: [3]MP-NB **7c**: [4]MP-BN

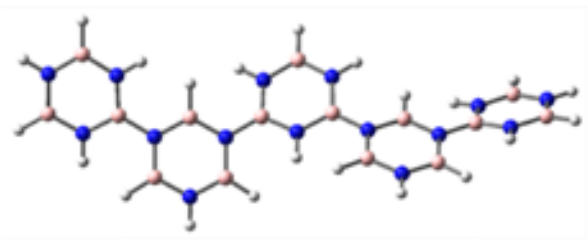
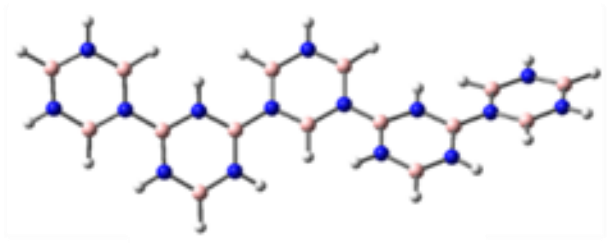

7d: [5]MP-BN **7e**: [5]MP-NB

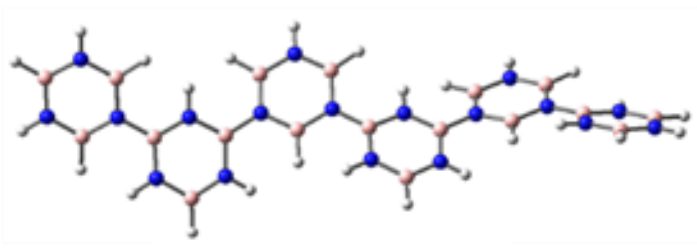

7f: [6]MP-BN

Supplementary Fig. 4 | Molecular structures of metaphenylene-type borazines (**7a: [3]MP-BN** - **7e: [6]MP-BN**) optimized at the PBE0/6-31G(d) level of the theory.

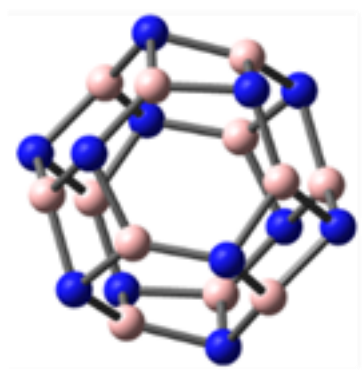
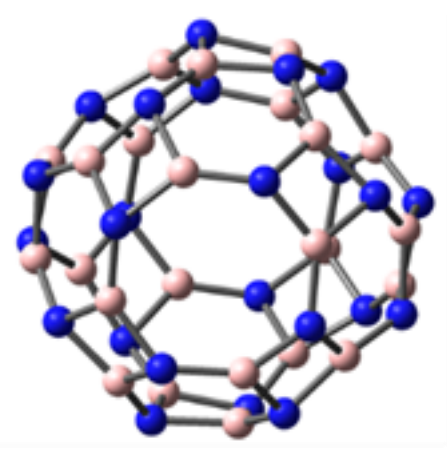
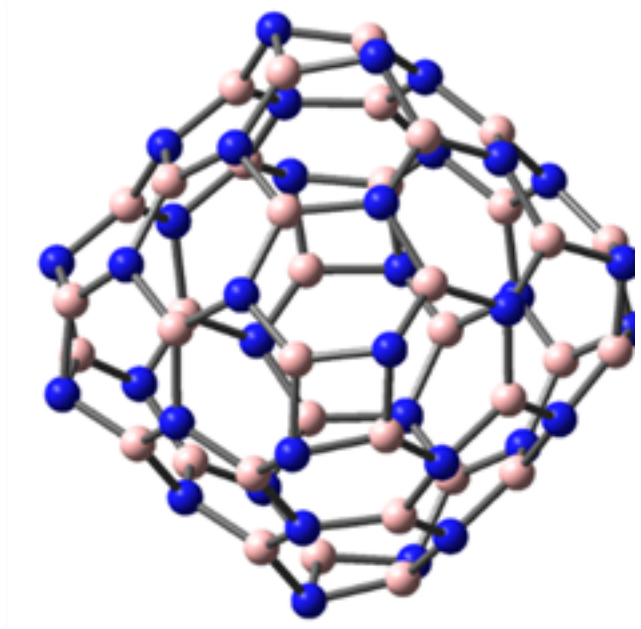

8a: $B_{12}N_{12}$-fullerene **8b**: $B_{24}N_{24}$-fullerene **8c**: $B_{36}N_{36}$-fullerene

Supplementary Fig. 5 | Molecular structures of fullerenes-type borazines (**8a: $B_{12}N_{12}$-fullerene** - **8c: $B_{36}N_{36}$-fullerene**) optimized at the PBE0/6-31G(d) level of the theory.

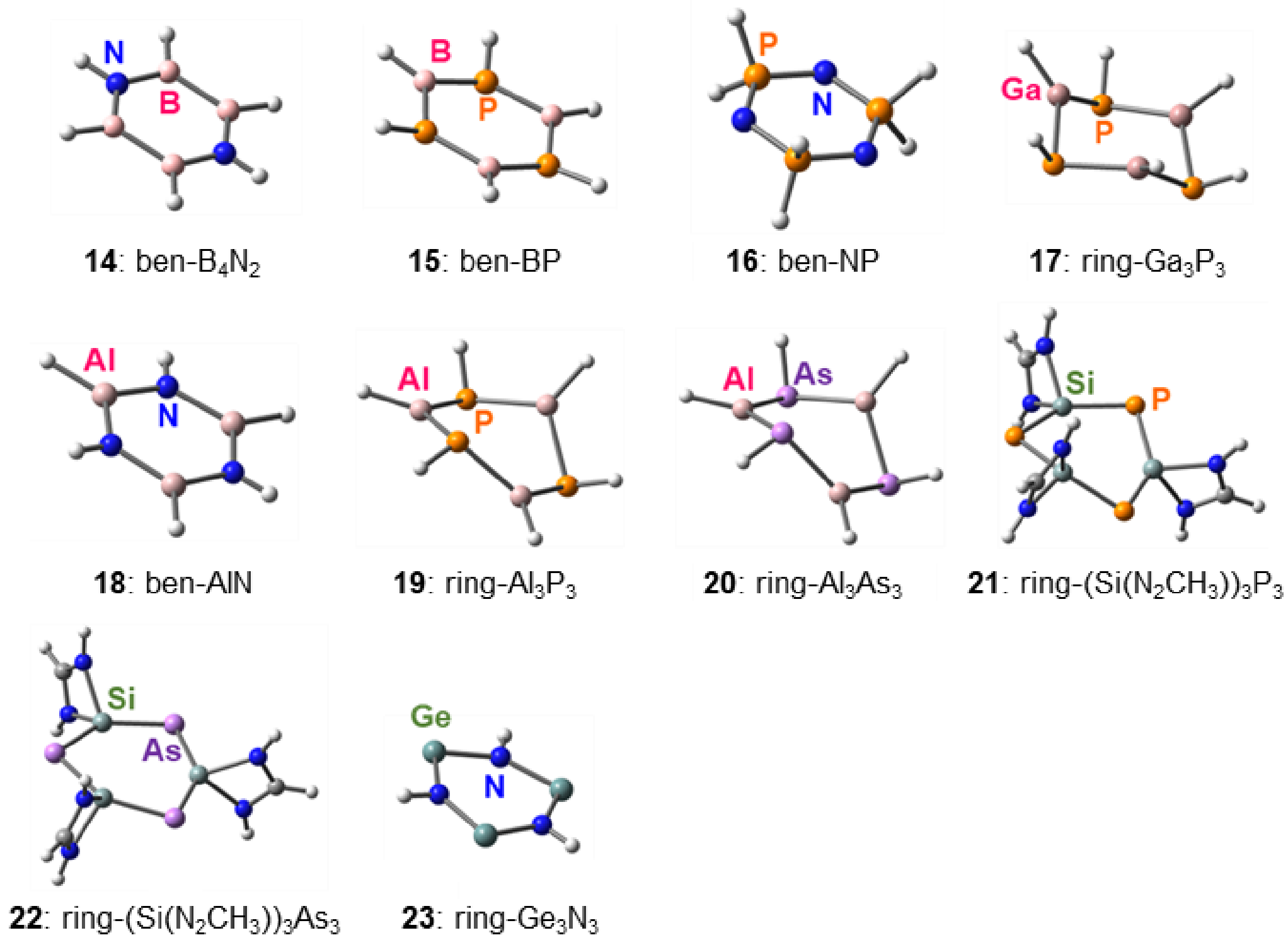

Supplementary Fig. 6 | Molecular structures of inorganic-type benzenes and cyclohexanes other than borazines (**14: ben-B_4N_2** - **23: ring-Ge_3N_3**) optimized at the PBE0/6-31G(d) level of the theory.

Supplementary Method: details of rate constant calculations

Taking into account the nuclear quantum effect and using Fermi's golden rule, k_{F}, k_{NR}, k_{ISC}, and k_{RISC} are expressed as follows.[1, 2] The mass-weighted coordinate system and atomic unit are used throughout.

$$k_{\mathrm{F}} = \frac{2E_{\mathrm{F}}^{2} f}{c^{3}} \tag{S1}$$

$$f = \frac{2}{3} E_{\mathrm{F}} \mu_{\mathrm{F}}^{2} \tag{S2}$$

$$\begin{aligned} k_{\mathrm{NR}} = \frac{2\pi}{\Delta E_{\mathrm{FC}}^{2}} \sum_{m} \omega_{m}^{2} V_{m}^{2} \sinh\left(\frac{1}{2}\beta\omega_{m}\right) \sum_{\nu_{m}} \exp\left\{-\left(\frac{1}{2}+\nu_{m}\right)\beta\omega_{m}\right\}\{\nu_{m} L(E_{\mathrm{if}}+\omega_{m}) \\ + (\nu_{m}+1) L(E_{\mathrm{if}}-\omega_{m})\} \end{aligned} \tag{S3}$$

$$k_{\mathrm{ISC}} = 2\pi |\mathrm{SOC}|^{2} L(E_{\mathrm{if}}) X(E_{\mathrm{if}}) \tag{S4}$$

$$k_{\mathrm{RISC}} = \frac{2}{3}\pi |\mathrm{SOC}|^{2} L(E_{\mathrm{if}}) X(E_{\mathrm{if}}) \tag{S5}$$

where the definition of characters is summarized below.

E_{F}	Fluorescence energy
c	Speed of light
ΔE_{FC}	Franck–Condon energy difference between S_1 and S_0
ω_m	Angular frequency of mode m
V_m	$\frac{\mathbf{d}_{\mathrm{if}}}{\Delta E_{\mathrm{FC}}} \cdot \mathbf{e}_m$ (Vibronic coupling between the initial and final states projected onto mode m)
$\mathbf{d}_{\mathrm{if}}$	Non-adiabatic coupling vector between the initial and final states
β	Inverse temperature
ν_m	Vibrational quantum number of mode ν_m
E_{if}	The energy of final state minus the energy of initial state (in the present work, approximated by the vertical energy difference)
$L(E)$	$\frac{1}{\pi}\frac{\gamma}{E^2+\gamma^2}$ (Lorentzian distribution function)
γ	Width of density-of-state distribution (set to 1000 cm^{-1})
$\lvert\mathrm{SOC}\rvert$	Spin-orbit coupling norm between S_1 and T_1
$X(E)$	Boltzmann weight ($\exp(-\beta E)$) when $E > 0$, otherwise 1

Supplementary Table 1 | Calculated data for inorganic-type benzenes, 1, 2a-k, 3, 4e, 5, 6e, 7f, 8a, and 9-11. ΔE_{ST}, $E(S_1)$ and $E(T_1)$ are calculated with the SCS-CC2/def2-TZVP//PBE0/6-31G(d) and ADC(2)/def2-TZVP//PBE0/6-31G(d) methods. μ_F, f, and k_F are calculated with the SCS-CC2/def2-TZVP//PBE0/6-31G(d) method.

	Molecular formula	Point group	SCS-CC2						ADC(2)		
			ΔE_{ST} (meV)	$E(S_1)$ (eV)	$E(T_1)$ (eV)	μ_F (au)	f	k_F (s^{-1})	ΔE_{ST} (meV)	$E(S_1)$ (eV)	$E(T_1)$ (eV)
**1**: ben-BN	$B_3N_3H_6$	D_{3h}	**−295**	6.83	7.13	0.0000	0.0	0.0	**−193**	6.76	6.96
**2a**: naph-BN	$B_5N_5H_8$	C_{2v}	**−95**	6.64	6.73	0.1502	3.7×10^{-3}	7.0×10^{6}	34	6.52	6.48
**2b**: anth-BN	$B_7N_7H_{10}$	C_{2v}	**−21**	6.63	6.65	0.4159	2.8×10^{-2}	$\mathbf{5.4\times10^{7}}$	106	6.48	6.38
**2c**: phenan-BN	$B_7N_7H_{10}$	C_s	**−127**	6.57	6.70	0.2990	1.4×10^{-2}	$\mathbf{2.7\times10^{7}}$	**−21**	6.42	6.44
**2d**: tetra-BN	$B_9N_9H_{12}$	C_{2v}	**−51**	6.62	6.67	0.7023	8.0×10^{-2}	$\mathbf{1.5\times10^{8}}$	30	6.42	6.39
**2e**: cryc-BN	$B_9N_9H_{12}$	C_s	**−59**	6.56	6.62	0.1300	2.7×10^{-3}	5.1×10^{6}	47	6.40	6.35
**2f**: pyr-BN	$B_8N_8H_{10}$	C_{2v}	**−221**	6.54	6.76	0.0562	5.1×10^{-4}	9.4×10^{5}	**−115**	6.39	6.50
**2g**: phenal-NB	$B_7N_6H_9$	D_{3h}	**−260**	6.59	6.85	0.0000	0.0	0.0	**−130**	6.46	6.59
**2h**: phenal-BN	$B_6N_7H_9$	D_{3h}	**−128**	6.54	6.67	0.0000	0.0	0.0	**−34**	6.39	6.43
**2i**: olymp-NB	$B_{10}N_9H_{11}$	C_{2v}	**−243**	6.54	6.78	0.0284	1.3×10^{-4}	2.4×10^{5}	**−124**	6.39	6.51
**2j**: olymp-BN	$B_9N_{10}H_{11}$	C_{2v}	**−97**	6.53	6.63	0.2545	1.0×10^{-2}	$\mathbf{1.9\times10^{7}}$	**−49**	6.37	6.42
**2k**: bis-BN	$B_{14}N_{14}H_{14}$	C_{2v}	**−57**	6.54	6.59	0.2110	7.1×10^{-3}	$\mathbf{1.3\times10^{7}}$	51	6.34	6.29
**3**: (6, 6)NB-BN	$B_{24}N_{24}H_{24}$	S_6	**−141**	6.26	6.40	0.0000	0.0	0.0	**−54**	6.02	6.08
**4e**: [6]CPP-BN	$B_{18}N_{18}H_{24}$	S_6	**−145**	6.26	6.40	0.0000	0.0	0.0	**−70**	6.04	6.11
**5**: [6]CMP-BN	$B_{18}N_{18}H_{24}$	C_{3v}	**−186**	6.45	6.64	0.0000	0.0	0.0	**−75**	6.28	6.35
**6e**: [6]PP-BN	$B_{18}N_{18}H_{26}$	C_2	**−77**	6.58	6.66	0.0458	3.4×10^{-4}	6.4×10^{5}	35	6.42	6.38
**7f**: [6]MP-BN	$B_{18}N_{18}H_{26}$	C_1	**−214**	6.49	6.70	0.0772	9.5×10^{-4}	1.7×10^{6}	**−78**	6.35	6.43
**8a**: $B_{12}N_{12}$-fullerene	$B_{12}N_{12}$	T_h	**−134**	6.47	6.60	0.0000	0.0	0.0	**−71**	6.16	6.23
**9**: ben-BS	$B_3S_3H_3$	D_{3h}	**−109**	5.02	5.13	0.0000	0.0	0.0	26	4.93	4.91
**10**: ben-BSe	$B_3Se_3H_3$	D_{3h}	**−160**	4.42	4.58	0.0000	0.0	0.0	**−47**	4.30	4.34
**11**: ben-BO	$B_3O_3H_3$	D_{3h}	326	8.11	7.79	0.0000	0.0	0.0	279	7.78	7.50
**12**: ben-GaN	$Ga_3N_3H_6$	D_{3h}	**−230**	5.52	5.75	0.0000	0.0	0.0	**−160**	5.37	5.53
**13**: prism-GaN	$Ga_3N_3H_6$	C_s	507	4.11	3.61	0.7097	5.1×10^{-2}	$\mathbf{3.7\times10^{7}}$	518	3.95	3.43

Supplementary Table 2 | Calculated data for inorganic-type benzenes, 4d-k, 6a-k, 7a-f, and 8a-c. ΔE_{ST}, $E(S_1)$ and $E(T_1)$ are calculated with the SCS-CC2/def2-TZVP//PBE0/6-31G(d) and ADC(2)/def2-TZVP//PBE0/6-31G(d) methods. μ_F, f, and k_F are calculated with the SCS-CC2/def2-TZVP//PBE0/6-31G(d) method. For **6i-k**, μ_F, f, and k_F were not obtained at the SCS-CC2/def2-TZVP level, because the electronic state calculations failed to converge.

	Molecular Formula	Point group	SCS-CC2 ΔE_{ST} (meV)	SCS-CC2 $E(S_1)$ (eV)	SCS-CC2 $E(T_1)$ (eV)	SCS-CC2 μ_F (au)	SCS-CC2 f	SCS-CC2 k_F (s^{-1})	ADC(2) ΔE_{ST} (meV)	ADC(2) $E(S_1)$ (eV)	ADC(2) $E(T_1)$ (eV)
**4d**: [5]CPP-BN	$B_{15}N_{15}H_{20}$	C_{5h}	**−133**	6.18	6.32	0.0000	0.0	0.0	**−61**	5.96	6.02
**4e**: [6]CPP-BN	$B_{18}N_{18}H_{24}$	S_6	**−145**	6.26	6.40	0.0000	0.0	0.0	**−70**	6.04	6.11
**4f**: [7]CPP-BN	$B_{21}N_{21}H_{28}$	C_{7h}	**−148**	6.31	6.45	0.0000	0.0	0.0	**−73**	6.10	6.17
**4g**: [8]CPP-BN	$B_{24}N_{24}H_{32}$	C_{8h}	**−148**	6.34	6.49	0.0000	0.0	0.0	**−72**	6.13	6.20
**4h**: [9]CPP-BN	$B_{27}N_{27}H_{36}$	C_{9h}	**−147**	6.37	6.51	0.0000	0.0	0.0	**−71**	6.16	6.23
**4i**: [10]CPP-BN	$B_{30}N_{30}H_{40}$	C_{10h}	**−146**	6.39	6.53	0.0000	0.0	0.0	**−70**	6.18	6.25
**4j**: [11]CPP-BN	$B_{33}N_{33}H_{44}$	C_S	**−145**	6.40	6.55	0.0007	7.0×10^{-8}	1.2×10^{2}	**−70**	6.19	6.26
**4k**: [12]CPP-BN	$B_{36}N_{36}H_{48}$	S_{12}	**−144**	6.41	6.56	0.0000	0.0	0.0	**−69**	6.21	6.28
**6a**: [2]PP-BN	$B_6N_6H_{10}$	C_2	**−156**	6.65	6.81	0.1295	2.7×10^{-3}	5.2×10^{6}	**−23**	6.54	6.56
**6b**: [3]PP-BN	$B_9N_9H_{14}$	C_2	**−109**	6.61	6.72	0.0491	3.9×10^{-4}	7.4×10^{5}	12	6.47	6.46
**6c**: [4]PP-BN	$B_{12}N_{12}H_{18}$	C_2	**−94**	6.60	6.69	0.0727	8.5×10^{-4}	1.6×10^{6}	21	6.44	6.42
**6d**: [5]PP-BN	$B_{15}N_{15}H_{22}$	C_2	**−84**	6.59	6.67	0.0156	3.9×10^{-5}	7.4×10^{4}	28	6.43	6.40
**6e**: [6]PP-BN	$B_{18}N_{18}H_{26}$	C_2	**−77**	6.58	6.66	0.0458	3.4×10^{-4}	6.4×10^{5}	35	6.42	6.38
**6f**: [7]PP-BN	$B_{21}N_{21}H_{30}$	C_2	**−71**	6.58	6.65	0.0089	1.3×10^{-5}	2.4×10^{4}	39	6.41	6.37
**6g**: [8]PP-BN	$B_{24}N_{24}H_{34}$	C_2	**−67**	6.58	6.64	0.0360	2.1×10^{-4}	3.9×10^{5}	21	6.39	6.37
**6h**: [9]PP-BN	$B_{27}N_{27}H_{38}$	C_2	**−64**	6.58	6.64	0.0198	6.3×10^{-5}	1.2×10^{5}	8	6.37	6.36
**6i**: [10]PP-BN	$B_{30}N_{30}H_{42}$	C_2	**−63**	6.57	6.64	–	–	–	**−3**	6.37	6.36
**6j**: [11]PP-BN	$B_{33}N_{33}H_{46}$	C_2	**−73**	6.56	6.63	–	–	–	**−11**	6.35	6.36
**6k**: [12]PP-BN	$B_{36}N_{36}H_{50}$	C_2	**−81**	6.56	6.63	–	–	–	**−17**	6.34	6.36
**7a**: [3]MP-BN	$B_9N_9H_{14}$	C_2	**−127**	6.64	6.77	0.2572	1.1×10^{-2}	$\mathbf{2.1\times10^{7}}$	6	6.52	6.51
**7b**: [3]MP-NB	$B_9N_9H_{14}$	C_2	**−217**	6.50	6.72	0.1042	1.7×10^{-3}	3.2×10^{6}	**−101**	6.35	6.45
**7c**: [4]MP-BN	$B_{12}N_{12}H_{18}$	C_1	**−222**	6.50	6.72	0.0812	1.0×10^{-3}	1.9×10^{6}	**−100**	6.35	6.45
**7d**: [5]MP-BN	$B_{15}N_{15}H_{22}$	C_2	**−227**	6.49	6.72	0.1110	2.0×10^{-3}	3.6×10^{6}	**−110**	6.34	6.45
**7e**: [5]MP-NB	$B_{15}N_{15}H_{22}$	C_2	**−210**	6.49	6.70	0.0398	2.5×10^{-4}	4.6×10^{5}	**−87**	6.34	6.43
**7f**: [6]MP-BN	$B_{18}N_{18}H_{26}$	C_1	**−214**	6.49	6.70	0.0772	9.5×10^{-4}	1.7×10^{6}	**−79**	6.35	6.43
**8a**: $B_{12}N_{12}$-fullerene	$B_{12}N_{12}$	T_h	**−134**	6.47	6.60	0.0000	0.0	0.0	**−71**	6.16	6.23
**8b**: $B_{24}N_{24}$-fullerene	$B_{24}N_{24}$	O	27	5.87	5.84	0.0000	0.0	0.0	32	5.35	5.31
**8c**: $B_{36}N_{36}$-fullerene	$B_{36}N_{36}$	T_d	28	6.11	6.08	0.0204	6.2×10^{-5}	1.0×10^{5}	27	5.57	5.55

Supplementary Table 3 | Calculated data for inorganic-type benzenes, 9-23. ΔE_{ST}, $E(S_1)$ and $E(T_1)$ are calculated with the SCS-CC2/def2-TZVP//PBE0/6-31G(d) and ADC(2)/def2-TZVP//PBE0/6-31G(d) methods. μ_F, f, and k_F are calculated with the SCS-CC2/def2-TZVP//PBE0/6-31G(d) method.

			SCS-CC2						ADC(2)		
	Molecular Formula	Point group	ΔE_{ST} (meV)	$E(S_1)$ (eV)	$E(T_1)$ (eV)	μ_F (au)	f	k_F (s^{-1})	ΔE_{ST} (meV)	$E(S_1)$ (eV)	$E(T_1)$ (eV)
**9**: ben-BS	$B_3S_3H_3$	D_{3h}	**−109**	5.02	5.13	0.0000	0.0	0.0	26	4.93	4.91
**10**: ben-BSe	$B_3Se_3H_3$	D_{3h}	**−160**	4.42	4.58	0.0000	0.0	0.0	**−47**	4.30	4.34
**11**: ben-BO	$B_3O_3H_3$	D_{3h}	326	8.11	7.79	0.0000	0.0	0.0	279	7.78	7.50
**12**: ben-GaN	$Ga_3N_3H_6$	D_{3h}	**−230**	5.52	5.75	0.0000	0.0	0.0	**−160**	5.37	5.53
**13**: prism-GaN	$Ga_3N_3H_6$	C_s	507	4.11	3.61	0.7097	5.1×10^{-2}	**3.7×10^{7}**	518	3.95	3.43
**14**: ben-B_4N_2	$B_4N_2H_6$	D_{2h}	613	4.23	3.62	0.0000	0.0	0.0	636	4.05	3.41
**15**: ben-BP	$B_3P_3H_6$	C_{3v}	110	3.72	3.61	0.0000	0.0	0.0	227	3.70	3.47
**16**: ben-NP	$N_3P_3H_6$	D_{3h}	174	6.84	6.67	0.0000	0.0	0.0	208	6.59	6.38
**17**: ring-Ga_3P_3	$Ga_3P_3H_6$	C_{3v}	610	4.38	3.77	0.7473	6.0×10^{-2}	**5.0×10^{7}**	603	4.25	3.65
**18**: ben-AlN	$Al_3N_3H_6$	D_{3h}	194	6.12	5.93	0.0000	0.0	0.0	251	6.01	5.76
**19**: ring-Al_3P_3	$Al_3P_3H_6$	C_s	493	4.69	4.19	0.2229	5.7×10^{-3}	5.4×10^{6}	508	4.61	4.10
**20**: ring-Al_3As_3	$Al_3As_3H_6$	C_s	452	4.42	3.96	0.4043	1.8×10^{-2}	**1.5×10^{7}**	462	4.31	3.85
**21**: ring-$(Si(N_2CH_3))_3P_3$	$Si_3P_3H_3$	C_s	102	4.01	3.90	0.2206	4.8×10^{-3}	3.3×10^{6}	139	3.76	3.62
**22**: ring-$(Si(N_2CH_3))_3As_3$	$Si_3As_3H_3$	C_s	127	4.00	3.87	0.0001	0.0	0.0	132	3.61	3.48
**23**: ring-Ge_3N_3	$Ge_3N_3H_3$	D_{3h}	1081	3.92	2.84	1.1101	1.2×10^{-1}	**7.9×10^{7}**	1128	3.81	2.69

Supplementary Table 4 | Calculated data for inorganic-type benzenes. ΔE_{ST}, $E(S_1)$, $E(T_1)$, μ_F, f, and k_F are calculated with the SCS-CC2/cc-pVTZ//MP2/6-31G(d) methods.

	Molecular formula	Point group	ΔE_{ST} (meV)	$E(S_1)$ (eV)	$E(T_1)$ (eV)	μ_F (au)	f	k_F (s^{-1})
**1**: ben-BN	$B_3N_3H_6$	D_{3h}	**−303**	6.81	7.11	0.00000	0.00000	0.0
**2a**: naph-BN	$B_5N_5H_8$	C_{2v}	**−101**	6.61	6.71	0.17485	0.00495	9.4×10^6
**2b**: anth-BN	$B_7N_7H_{10}$	C_{2v}	**−31**	6.60	6.63	0.47220	0.03604	**6.8×10^7**
**2c**: phenan-BN	$B_7N_7H_{10}$	C_s	**−140**	6.54	6.68	0.27374	0.01201	**2.2×10^7**
**2d**: tetra-BN	$B_9N_9H_{12}$	C_{2v}	**−58**	6.59	6.64	0.70886	0.08108	**1.5×10^8**
**2e**: cryc-BN	$B_9N_9H_{12}$	C_s	**−75**	6.53	6.61	0.14548	0.00339	6.3×10^6
**2f**: pyr-BN	$B_8N_8H_{10}$	C_{2v}	**−242**	6.50	6.75	0.08484	0.00115	2.1×10^6
**2g**: phenal-NB	$B_7N_6H_9$	D_{3h}	**−270**	6.54	6.81	0.00000	0.00000	0.0
**2h**: phenal-BN	$B_6N_7H_9$	D_{3h}	**−143**	6.53	6.67	0.00000	0.00000	0.0
**2i**: olymp-NB	$B_{10}N_9H_{11}$	C_{2v}	**−253**	6.50	6.75	0.00560	0.00001	9.2×10^3
**2j**: olymp-BN	$B_9N_{10}H_{11}$	C_{2v}	**−174**	6.51	6.69	0.28435	0.01290	**2.4×10^7**
**2k**: bis-BN	$B_{14}N_{14}H_{14}$	C_{2v}	**−68**	6.50	6.57	0.24428	0.00951	**1.7×10^7**
**3**: (6, 6)NB-BN	$B_{24}N_{24}H_{24}$	S_6	**−173**	6.24	6.41	0.00000	0.00000	0.0
**4e**: [6]CPP-BN	$B_{18}N_{18}H_{24}$	S_6	**−168**	6.23	6.40	0.00000	0.00000	0.0
**5**: [6]CMP-BN	$B_{18}N_{18}H_{24}$	C_{3v}	**−198**	6.43	6.63	0.00000	0.00000	0.0
**6e**: [6]PP-BN	$B_{18}N_{18}H_{26}$	C_2	**−99**	6.58	6.68	0.06030	0.00059	1.1×10^6
**7f**: [6]MP-BN	$B_{18}N_{18}H_{26}$	C_1	**−231**	6.46	6.69	0.13094	0.00271	4.9×10^6
**8a**: $B_{12}N_{12}$-fullerene	$B_{12}N_{12}$	T_h	**−134**	6.48	6.61	0.00000	0.00000	0.0
**9**: ben-BS	$B_3S_3H_3$	D_{3h}	**−126**	5.06	5.18	0.00000	0.00000	0.0
**10**: ben-BSe	$B_3Se_3H_3$	D_{3h}	**−168**	4.39	4.56	0.00000	0.00000	0.0
**11**: ben-BO	$B_3O_3H_3$	D_{3h}	265	8.08	7.82	0.00000	0.00000	0.0
**12**: ben-GaN	$Ga_3N_3H_6$	D_{3h}	**−101**	5.30	5.40	0.00000	0.00000	0.0
**13**: prism-GaN	$Ga_3N_3H_6$	C_s	497	4.05	3.56	0.72321	0.05195	**3.7×10^7**

Supplementary Table 5 | Calculated data for inorganic-type benzenes. ΔE_{ST}, $E(S_1)$, $E(T_1)$, μ_F, f, and k_F are calculated with the SCS-CC2/cc-pVTZ//TD-PBE0/6-31G(d) method at the **S_1-optimized** geometries.

	Molecular formula	Point group	ΔE_{ST} (meV)	$E(S_1)$ (eV)	$E(T_1)$ (eV)	μ_F (au)	f	k_F (s^{-1})	k_{NR} (s^{-1})	k_{ISC} (s^{-1})	k_{RISC} (s^{-1})
**1**: ben-BN	$B_3N_3H_6$	C_1	**−362**	6.42	6.78	0.00000	0.00000	0.0	4.9×10^{7}	3.2×10^{1}	**3.8×10^{7}**
**2a**: naph-BN	$B_5N_5H_8$	C_s	234	6.26	6.03	0.83135	0.10608	**1.8×10^{8}**	8.3×10^{6}	6.7×10^{1}	7.8×10^{-3}
**2b**: anth-BN	$B_7N_7H_{10}$	C_{2v}	48	6.24	6.19	0.73325	0.08222	**1.4×10^{8}**	1.1×10^{7}	0.0	0.0
**2c**: phenan-BN	$B_7N_7H_{10}$	C_1	12	2.45	2.44	0.28518	0.00488	1.3×10^{6}	3.2×10^{6}	5.9×10^{6}	**3.6×10^{6}**
**2d**: tetra-BN	$B_9N_9H_{12}$	C_{2v}	**−34**	6.32	6.35	0.69178	0.07407	**1.3×10^{8}**	8.1×10^{5}	0.0	0.0
**2e**: cryc-BN	$B_9N_9H_{12}$	C_1	168	5.42	5.25	0.93644	0.11640	**1.5×10^{8}**	4.2×10^{5}	1.0×10^{7}	1.5×10^{4}
**2f**: pyr-BN	$B_8N_8H_{10}$	C_{2v}	**−194**	6.36	6.55	0.04775	0.00036	6.2×10^{5}	4.5×10^{7}	1.9×10^{4}	**3.3×10^{7}**
**2g**: phenal-NB	$B_7N_6H_9$	C_s	**−175**	6.35	6.52	0.45736	0.03252	**5.7×10^{7}**	2.7×10^{7}	1.2×10^{0}	1.1×10^{3}
**2h**: phenal-BN	$B_6N_7H_9$	C_{2v}	**−133**	6.36	6.50	0.00000	0.00000	0.0	3.7×10^{7}	0.0	0.0
**2i**: olymp-NB	$B_{10}N_9H_{11}$	C_{2v}	**−181**	6.40	6.58	0.38457	0.02320	**4.1×10^{7}**	1.3×10^{7}	0.0	0.0
**2j**: olymp-BN	$B_9N_{10}H_{11}$	C_{2v}	**−167**	6.39	6.56	0.30963	0.01501	**2.7×10^{7}**	2.7×10^{7}	0.0	0.0
**2k**: bis-BN	$B_{14}N_{14}H_{14}$	C_s	**−14**	6.41	6.42	1.08416	0.18459	**3.3×10^{8}**	4.4×10^{6}	8.8×10^{3}	1.5×10^{4}
**3**: (6, 6)NB-BN	$B_{24}N_{24}H_{24}$	C_1	33	3.16	3.13	0.29886	0.00692	3.0×10^{6}	8.7×10^{6}	1.1×10^{8}	**3.2×10^{7}**
**4e**: [6]CPP-BN	$B_{18}N_{18}H_{24}$	C_i	**−181**	6.09	6.27	0.00000	0.00000	0.0	2.7×10^{6}	5.6×10^{2}	6.0×10^{5}
**5**: [6]CMP-BN	$B_{18}N_{18}H_{24}$	C_1	38	2.14	2.10	0.19550	0.00200	4.0×10^{5}	4.2×10^{7}	5.0×10^{7}	**1.2×10^{7}**
**6e**: [6]PP-BN	$B_{18}N_{18}H_{26}$	C_1	**−7**	6.50	6.50	4.57540	3.33121	**6.1×10^{9}**	1.1×10^{4}	1.0×10^{4}	1.4×10^{4}
**7f**: [6]MP-BN	$B_{18}N_{18}H_{26}$	C_1	32	1.90	1.87	0.15565	0.00113	1.8×10^{5}	5.9×10^{6}	4.0×10^{7}	**1.2×10^{7}**

Supplementary Table 6 | Calculated data for inorganic-type benzenes. ΔE_{ST}, $E(S_1)$, $E(T_1)$, μ_F, f, and k_F are calculated with the SCS-CC2/cc-pVDZ//PBE0/6-31G(d) methods.

	Molecular formula	Point group	ΔE_{ST} (meV)	$E(S_1)$ (eV)	$E(T_1)$ (eV)	μ_F (au)	f	k_F (s^{-1})
**1**: ben-BN	$B_3N_3H_6$	D_{3h}	**−240**	6.86	7.10	0.00000	0.00000	0.0
**2a**: naph-BN	$B_5N_5H_8$	C_{2v}	**−75**	6.63	6.70	0.19331	0.00607	**1.2×10^7**
**2b**: anth-BN	$B_7N_7H_{10}$	C_{2v}	**−93**	6.61	6.70	0.51599	0.04311	**8.2×10^7**
**2c**: phenan-BN	$B_7N_7H_{10}$	C_s	**−161**	6.56	6.72	0.24700	0.00980	**1.8×10^7**
**2d**: tetra-BN	$B_9N_9H_{12}$	C_{2v}	**−55**	6.59	6.64	0.75546	0.09210	**1.7×10^8**
**2e**: cryc-BN	$B_9N_9H_{12}$	C_s	**−86**	6.55	6.63	0.19726	0.00624	**1.2×10^7**
**2f**: pyr-BN	$B_8N_8H_{10}$	C_{2v}	**−278**	6.51	6.79	0.11343	0.00205	3.8×10^6
**2g**: phenal-NB	$B_7N_6H_9$	D_{3h}	**−294**	6.54	6.83	0.00000	0.00000	0.0
**2h**: phenal-BN	$B_6N_7H_9$	D_{3h}	**−155**	6.56	6.71	0.00000	0.00000	0.0
**2i**: olymp-NB	$B_{10}N_9H_{11}$	C_{2v}	**−277**	6.49	6.77	0.02235	0.00008	1.5×10^5
**2j**: olymp-BN	$B_9N_{10}H_{11}$	C_{2v}	**−193**	6.52	6.71	0.33222	0.01763	**3.3×10^7**
**2k**: bis-BN	$B_{14}N_{14}H_{14}$	C_{2v}	**−61**	6.51	6.58	0.27012	0.01164	**2.1×10^7**
**3**: (6, 6)NB-BN	$B_{24}N_{24}H_{24}$	S_6	**−201**	6.25	6.45	0.00000	0.00000	0.0
**4e**: [6]CPP-BN	$B_{18}N_{18}H_{24}$	S_6	**−192**	6.22	6.41	0.00000	0.00000	0.0
**5**: [6]CMP-BN	$B_{18}N_{18}H_{24}$	C_{3v}	**−216**	6.50	6.72	0.00000	0.00000	0.0
**6e**: [6]PP-BN	$B_{18}N_{18}H_{26}$	C_2	4	6.67	6.66	0.07185	0.00084	1.6×10^6
**7f**: [6]MP-BN	$B_{18}N_{18}H_{26}$	C_1	**−181**	6.53	6.71	0.06655	0.00071	1.3×10^6
**8a**: $B_{12}N_{12}$-fullerene	$B_{12}N_{12}$	T_h	**−137**	6.43	6.57	0.00000	0.00000	0.0
**9**: ben-BS	$B_3S_3H_3$	D_{3h}	**−185**	5.10	5.28	0.00000	0.00000	0.0
**10**: ben-BSe	$B_3Se_3H_3$	D_{3h}	**−187**	4.46	4.64	0.00000	0.00000	0.0
**11**: ben-BO	$B_3O_3H_3$	D_{3h}	249	8.14	7.89	0.00000	0.00000	0.0
**12**: ben-GaN	$Ga_3N_3H_6$	D_{3h}	**−230**	5.42	5.66	0.00000	0.00000	0.0
**13**: prism-GaN	$Ga_3N_3H_6$	C_s	577	4.21	3.63	0.71795	0.05314	**4.1×10^7**

Supplementary Table 7 | Calculated data for inorganic-type benzenes. ΔE_{ST}, $E(S_1)$, $E(T_1)$, μ_F, f, and k_F are calculated with the SCS-CC2/cc-pVDZ//MP2/6-31G(d) methods.

	Molecular formula	Point group	ΔE_{ST} (meV)	$E(S_1)$ (eV)	$E(T_1)$ (eV)	μ_F (au)	f	k_F (s^{-1})
**1**: ben-BN	$B_3N_3H_6$	D_{3h}	**−244**	6.82	7.06	0.00000	0.00000	0.0
**2a**: naph-BN	$B_5N_5H_8$	C_{2v}	**−80**	6.58	6.66	0.20339	0.00667	**1.3×10^{7}**
**2b**: anth-BN	$B_7N_7H_{10}$	C_{2v}	**−20**	6.57	6.59	0.54006	0.04692	**8.8×10^{7}**
**2c**: phenan-BN	$B_7N_7H_{10}$	C_s	**−164**	6.52	6.68	0.24445	0.00954	**1.8×10^{7}**
**2d**: tetra-BN	$B_9N_9H_{12}$	C_{2v}	**−59**	6.55	6.61	0.75829	0.09225	**1.7×10^{8}**
**2e**: cryc-BN	$B_9N_9H_{12}$	C_s	**−92**	6.51	6.60	0.20621	0.00678	**1.2×10^{7}**
**2f**: pyr-BN	$B_8N_8H_{10}$	C_{2v}	**−282**	6.47	6.75	0.12387	0.00243	4.4×10^{6}
**2g**: phenal-NB	$B_7N_6H_9$	D_{3h}	**−295**	6.50	6.79	0.00000	0.00000	0.0
**2h**: phenal-BN	$B_6N_7H_9$	D_{3h}	**−159**	6.52	6.68	0.00000	0.00000	0.0
**2i**: olymp-NB	$B_{10}N_9H_{11}$	C_{2v}	**−281**	6.45	6.73	0.00826	0.00001	1.9×10^{4}
**2j**: olymp-BN	$B_9N_{10}H_{11}$	C_{2v}	**−196**	6.48	6.68	0.34194	0.01858	**3.4×10^{7}**
**2k**: bis-BN	$B_{14}N_{14}H_{14}$	C_{2v}	**−68**	6.47	6.54	0.28437	0.01282	**2.3×10^{7}**
**3**: (6, 6)NB-BN	$B_{24}N_{24}H_{24}$	S_6	**−205**	6.21	6.42	0.00000	0.00000	0.0
**4e**: [6]CPP-BN	$B_{18}N_{18}H_{24}$	S_6	**−197**	6.18	6.38	0.00000	0.00000	0.0
**5**: [6]CMP-BN	$B_{18}N_{18}H_{24}$	C_{3v}	**−209**	6.46	6.67	0.00000	0.00000	0.0
**6e**: [6]PP-BN	$B_{18}N_{18}H_{26}$	C_2	**−33**	6.63	6.66	0.08100	0.00107	2.0×10^{6}
**7f**: [6]MP-BN	$B_{18}N_{18}H_{26}$	C_1	**−198**	6.48	6.68	0.06781	0.00073	1.3×10^{6}
**8a**: $B_{12}N_{12}$-fullerene	$B_{12}N_{12}$	T_h	**−138**	6.44	6.58	0.00000	0.00000	0.0
**9**: ben-BS	$B_3S_3H_3$	D_{3h}	**−181**	5.12	5.30	0.00000	0.00000	0.0
**10**: ben-BSe	$B_3Se_3H_3$	D_{3h}	**−194**	4.43	4.62	0.00000	0.00000	0.0
**11**: ben-BO	$B_3O_3H_3$	D_{3h}	212	8.09	7.88	0.00000	0.00000	0.0
**12**: ben-GaN	$Ga_3N_3H_6$	D_{3h}	**−307**	5.18	5.49	0.00000	0.00000	0.0
**13**: prism-GaN	$Ga_3N_3H_6$	C_s	569	4.17	3.60	0.72972	0.05435	**4.1×10^{7}**

Supplementary Table 8 | Calculated data for inorganic-type benzenes. ΔE_{ST}, $E(S_1)$, $E(T_1)$, μ_F, f, and k_F are calculated with the SCS-CC2/cc-pVDZ//TD-PBE0/6-31G(d) method at the **S_1-optimized** geometries.

	Molecular formula	Point group	ΔE_{ST} (meV)	$E(S_1)$ (eV)	$E(T_1)$ (eV)	μ_F (au)	f	k_F (s^{-1})
**1**: ben-BN	$B_3N_3H_6$	C_1	**−264**	6.42	6.68	0.00000	0.00000	0.0
**2a**: naph-BN	$B_5N_5H_8$	C_s	276	6.22	5.95	0.87772	0.11743	**2.0×10^8**
**2b**: anth-BN	$B_7N_7H_{10}$	C_{2v}	65	6.18	6.12	0.77833	0.09174	**1.5×10^8**
**2c**: phenan-BN	$B_7N_7H_{10}$	C_1	16	2.29	2.27	0.29745	0.00496	1.1×10^6
**2d**: tetra-BN	$B_9N_9H_{12}$	C_{2v}	**−29**	6.26	6.28	0.73151	0.08201	**1.4×10^8**
**2e**: cryc-BN	$B_9N_9H_{12}$	C_1	207	5.38	5.17	0.98618	0.12816	**1.6×10^8**
**2f**: pyr-BN	$B_8N_8H_{10}$	C_{2v}	**−232**	6.32	6.55	0.00924	0.00001	2.3×10^4
**2g**: phenal-NB	$B_7N_6H_9$	C_s	**−199**	6.29	6.49	0.46425	0.03323	**5.7×10^7**
**2h**: phenal-BN	$B_6N_7H_9$	C_{2v}	**−148**	6.35	6.50	0.00000	0.00000	0.0
**2i**: olymp-NB	$B_{10}N_9H_{11}$	C_{2v}	**−192**	6.35	6.54	0.39820	0.02465	**4.3×10^7**
**2j**: olymp-BN	$B_9N_{10}H_{11}$	C_{2v}	**−187**	6.36	6.54	0.38200	0.02273	**4.0×10^7**
**2k**: bis-BN	$B_{14}N_{14}H_{14}$	C_s	**−10**	6.37	6.38	1.20226	0.22541	**4.0×10^8**
**3**: (6, 6)NB-BN	$B_{24}N_{24}H_{24}$	C_1	37	3.03	3.00	0.31818	0.00753	3.0×10^6
**4e**: [6]CPP-BN	$B_{18}N_{18}H_{24}$	C_i	0	6.02	6.02	0.00000	0.00000	0.0
**5**: [6]CMP-BN	$B_{18}N_{18}H_{24}$	C_1	41	2.02	1.98	0.20531	0.00209	3.7×10^5
**6e**: [6]PP-BN	$B_{18}N_{18}H_{26}$	C_1	**−9**	6.45	6.46	4.66876	3.44655	**6.2×10^9**
**7f**: [6]MP-BN	$B_{18}N_{18}H_{26}$	C_1	35	1.78	1.74	0.15545	0.00105	1.4×10^5

Supplementary Table 9 | Nuclear coordinates of S_0 geometry of **1: ben-BN** at the PBE0/6-31G(d) level of theory.

Element Symbol	*x* (Å)	*y* (Å)	*z* (Å)
N	0.000000	1.407318	0.000000
H	0.000000	2.416612	0.000000
N	1.218774	-0.703659	0.000000
H	2.092847	-1.208306	0.000000
N	-1.218774	-0.703659	0.000000
H	-2.092847	-1.208306	0.000000
B	1.253408	0.723655	0.000000
H	2.292570	1.323616	0.000000
B	-1.253408	0.723655	0.000000
H	-2.292570	1.323616	0.000000
B	0.000000	-1.447311	0.000000
H	0.000000	-2.647232	0.000000

Supplementary Table 10 | Nuclear coordinates of S_0 geometry of **2a: naph-BN** at the PBE0/6-31G(d) level of theory.

Element Symbol	*x* (Å)	*y* (Å)	*z* (Å)
B	0.000000	1.251777	1.459668
N	0.000000	0.000000	0.744317
B	0.000000	-1.251777	1.459668
B	0.000000	0.000000	-0.709957
N	0.000000	-1.261720	-1.397079
B	0.000000	-2.507648	-0.710363
N	0.000000	-2.467973	0.718731
N	0.000000	2.467973	0.718731
B	0.000000	2.507648	-0.710363
N	0.000000	1.261720	-1.397079
H	0.000000	3.548248	-1.308319
H	0.000000	3.339176	1.228458
H	0.000000	1.268652	2.658560
H	0.000000	-1.268652	2.658560
H	0.000000	-3.339176	1.228458
H	0.000000	-3.548248	-1.308319
H	0.000000	-1.277313	-2.407010
H	0.000000	1.277313	-2.407010

Supplementary Table 11 | Nuclear coordinates of S_0 geometry of **2b: anth-BN** at the PBE0/6-31G(d) level of theory.

Element Symbol	*x* (Å)	*y* (Å)	*z* (Å)
B	0.000000	2.498899	-1.467212
H	0.000000	2.510761	-2.665555
B	0.000000	0.000000	-1.458884
H	0.000000	0.000000	-2.656463
B	0.000000	-2.498899	-1.467212
H	0.000000	-2.510761	-2.665555

B	0.000000	3.763164	0.697815
H	0.000000	4.806100	1.291366
B	0.000000	1.255135	0.709193
B	0.000000	-1.255135	0.709193
B	0.000000	-3.763164	0.697815
H	0.000000	-4.806100	1.291366
N	0.000000	2.520291	1.390656
H	0.000000	2.541489	2.400440
N	0.000000	0.000000	1.398152
H	0.000000	0.000000	2.409010
N	0.000000	-2.520291	1.390656
H	0.000000	-2.541489	2.400440
N	0.000000	3.718187	-0.731077
H	0.000000	4.587596	-1.243772
N	0.000000	1.249682	-0.745872
N	0.000000	-1.249682	-0.745872
N	0.000000	-3.718187	-0.731077
H	0.000000	-4.587596	-1.243772

Supplementary Table 12 | Nuclear coordinates of S_0 geometry of **2c: phenan-BN** at the PBE0/6-31G(d) level of theory.

Element Symbol	*x* (Å)	*y* (Å)	*z* (Å)
H	-3.123862	2.160758	0.000000
H	-2.210762	-2.652564	0.000000
H	2.415011	-3.946243	0.000000
H	-0.103561	-4.005461	0.000000
H	2.654607	0.229059	0.000000
H	3.969367	-1.927964	0.000000
H	-3.194964	-0.353248	0.000000
H	-1.982673	4.419324	0.000000
H	0.551285	4.146482	0.000000
H	1.926403	2.083470	0.000000
B	-1.409333	3.365520	0.000000
B	-1.452244	0.856766	0.000000
B	0.724877	2.054903	0.000000
B	-1.555012	-1.648862	0.000000
B	0.669755	-0.485148	0.000000
B	0.541731	-2.994580	0.000000
B	2.770725	-1.864818	0.000000
N	-2.114346	2.132400	0.000000
N	0.018202	3.289639	0.000000
N	0.000000	0.812460	0.000000
N	-2.185256	-0.378230	0.000000
N	-0.115109	-1.709927	0.000000
N	2.097315	-0.612681	0.000000
N	1.963002	-3.044020	0.000000

Supplementary Table 13 | Nuclear coordinates of S_0 geometry of **2d: tetra-BN** at the PBE0/6-31G(d) level of theory.

Element Symbol	*x* (Å)	*y* (Å)	*z* (Å)
B	0.000000	3.745999	1.475837
H	0.000000	3.753296	2.674107
B	0.000000	1.247026	1.457438
H	0.000000	1.242148	2.654515
B	0.000000	-1.247026	1.457438
H	0.000000	-1.242148	2.654515
B	0.000000	5.018612	-0.684256
H	0.000000	6.063663	-1.274122
B	0.000000	2.510752	-0.705234
B	0.000000	0.000000	-0.717886
B	0.000000	-2.510752	-0.705234
N	0.000000	3.778190	-1.382035
H	0.000000	3.803467	-2.391688
N	0.000000	1.258666	-1.400253
H	0.000000	1.265392	-2.411054
N	0.000000	-1.258666	-1.400253
H	0.000000	-1.265392	-2.411054
N	0.000000	4.968308	0.744193
H	0.000000	5.835775	1.260157
N	0.000000	2.499853	0.749537
N	0.000000	0.000000	0.738225
H	0.000000	-5.835775	1.260157
H	0.000000	-3.803467	-2.391688
B	0.000000	-3.745999	1.475837
H	0.000000	-3.753296	2.674107
B	0.000000	-5.018612	-0.684256
H	0.000000	-6.063663	-1.274122
N	0.000000	-2.499853	0.749537
N	0.000000	-4.968308	0.744193
N	0.000000	-3.778190	-1.382035

Supplementary Table 14 | Nuclear coordinates of S_0 geometry of **2e: cryc-BN** at the PBE0/6-31G(d) level of theory.

Element Symbol	*x* (Å)	*y* (Å)	*z* (Å)
H	4.625097	-0.881071	0.000000
H	1.270920	2.650144	0.000000
H	-1.251606	-2.415816	0.000000
H	-3.530474	-1.386384	0.000000
H	3.306481	1.270691	0.000000
H	4.894333	-3.397760	0.000000
H	2.616818	-4.544208	0.000000
H	0.345840	-3.562518	0.000000
B	3.840673	-2.825031	0.000000
B	2.512947	-0.698397	0.000000
B	1.336001	-2.882702	0.000000

B	1.226209	1.449660	0.000000
B	0.000000	-0.719761	0.000000
B	-2.513977	-0.750605	0.000000
N	3.762423	-1.406377	0.000000
N	2.600445	-3.535063	0.000000
N	1.268483	-1.444930	0.000000
N	2.449997	0.735439	0.000000
N	-0.021417	0.735222	0.000000
N	-1.257350	-1.406353	0.000000
N	-2.547585	0.689268	0.000000
H	-0.541203	3.407998	0.000000
H	-2.661037	4.774154	0.000000
H	-4.719729	3.273478	0.000000
H	-4.841360	0.756026	0.000000
B	-1.302660	1.438261	0.000000
B	-2.629566	3.574217	0.000000
B	-3.815573	1.376122	0.000000
N	-1.394736	2.869645	0.000000
N	-3.829453	2.798356	0.000000

Supplementary Table 15 | Nuclear coordinates of S_0 geometry of **2f: pyr-BN** at the PBE0/6-31G(d) level of theory.

Element Symbol	*x* (Å)	*y* (Å)	*z* (Å)
B	0.000000	1.253173	2.911643
N	0.000000	1.257196	1.472699
B	0.000000	2.505660	0.748251
B	0.000000	0.000000	0.745241
N	0.000000	0.000000	-0.698383
B	0.000000	1.252120	-1.424667
N	0.000000	2.476272	-0.670074
N	0.000000	0.000000	3.590451
B	0.000000	-1.253173	2.911643
N	0.000000	-1.257196	1.472699
B	0.000000	-2.505660	0.748251
N	0.000000	-2.476272	-0.670074
B	0.000000	-1.252120	-1.424667
N	0.000000	-1.219556	-2.859488
B	0.000000	0.000000	-3.596984
N	0.000000	1.219556	-2.859488
H	0.000000	-3.360382	-1.158556
H	0.000000	-2.086629	-3.376935
H	0.000000	0.000000	-4.797164
H	0.000000	2.086629	-3.376935
H	0.000000	0.000000	4.600117
H	0.000000	2.281533	3.527647
H	0.000000	3.550898	1.335389
H	0.000000	3.360382	-1.158556
H	0.000000	-3.550898	1.335389
H	0.000000	-2.281533	3.527647

Supplementary Table 16 | Nuclear coordinates of S_0 geometry of **2g: phenal-NB** at the PBE0/6-31G(d) level of theory.

Element Symbol	*x* (Å)	*y* (Å)	*z* (Å)
B	1.249868	2.168447	0.000000
N	0.000000	1.450864	0.000000
B	-1.249868	2.168447	0.000000
B	0.000000	0.000000	0.000000
N	-1.256485	-0.725432	0.000000
B	-2.502864	-0.001806	0.000000
N	-2.463021	1.422026	0.000000
N	2.463021	1.422026	0.000000
B	2.502864	-0.001806	0.000000
N	1.256485	-0.725432	0.000000
B	1.252996	-2.166641	0.000000
N	0.000000	-2.844052	0.000000
B	-1.252996	-2.166641	0.000000
H	0.000000	-3.854025	0.000000
H	2.281599	-2.780974	0.000000
H	3.549193	-0.585436	0.000000
H	3.337683	1.927012	0.000000
H	1.267594	3.366410	0.000000
H	-1.267594	3.366410	0.000000
H	-3.337683	1.927012	0.000000
H	-3.549193	-0.585436	0.000000
H	-2.281599	-2.780974	0.000000

Supplementary Table 17 | Nuclear coordinates of S_0 geometry of **2h: phenal-BN** at the PBE0/6-31G(d) level of theory.

Element Symbol	*x* (Å)	*y* (Å)	*z* (Å)
N	1.262129	2.136437	0.000000
B	0.000000	1.447659	0.000000
N	-1.262129	2.136437	0.000000
N	0.000000	0.000000	0.000000
B	-1.253709	-0.723829	0.000000
N	-2.481273	0.024817	0.000000
B	-2.509838	1.449055	0.000000
B	2.509838	1.449055	0.000000
N	2.481273	0.024817	0.000000
B	1.253709	-0.723829	0.000000
N	1.219144	-2.161254	0.000000
B	0.000000	-2.898111	0.000000
N	-1.219144	-2.161254	0.000000
H	0.000000	-4.098191	0.000000
H	2.086509	-2.678221	0.000000
H	3.362662	-0.467860	0.000000
H	3.549138	2.049096	0.000000
H	1.276153	3.146081	0.000000
H	-1.276153	3.146081	0.000000

H	-3.549138	2.049096	0.000000
H	-3.362662	-0.467860	0.000000
H	-2.086509	-2.678221	0.000000

Supplementary Table 18 | Nuclear coordinates of S_0 geometry of **2i: olymp-NB** at the PBE0/6-31G(d) level of theory.

Element Symbol	*x* (Å)	*y* (Å)	*z* (Å)
N	0.000000	1.262579	2.728033
B	0.000000	0.000000	2.044653
N	0.000000	-1.262579	2.728033
N	0.000000	0.000000	0.597326
B	0.000000	-1.251883	-0.121769
N	0.000000	-2.509179	0.601171
B	0.000000	-2.507132	2.044687
B	0.000000	2.507132	2.044687
N	0.000000	2.509179	0.601171
B	0.000000	1.251883	-0.121769
N	0.000000	1.247230	-1.573020
B	0.000000	0.000000	-2.289264
N	0.000000	-1.247230	-1.573020
B	0.000000	3.752639	-0.125096
N	0.000000	3.710169	-1.549720
B	0.000000	2.496275	-2.294418
B	0.000000	-2.496275	-2.294418
N	0.000000	-3.710169	-1.549720
B	0.000000	-3.752639	-0.125096
H	0.000000	0.000000	-3.486273
H	0.000000	4.800847	0.455473
H	0.000000	4.583877	-2.055982
H	0.000000	2.511621	-3.491951
H	0.000000	-2.511621	-3.491951
H	0.000000	-4.583877	-2.055982
H	0.000000	-4.800847	0.455473
H	0.000000	3.537196	2.656202
H	0.000000	1.280599	3.738011
H	0.000000	-1.280599	3.738011
H	0.000000	-3.537196	2.656202

Supplementary Table 19 | Nuclear coordinates of S_0 geometry of **2j: olymp-BN** at the PBE0/6-31G(d) level of theory.

Element Symbol	*x* (Å)	*y* (Å)	*z* (Å)
B	0.000000	1.249695	-2.796936
N	0.000000	0.000000	-2.079855
B	0.000000	-1.249695	-2.796936
B	0.000000	0.000000	-0.625197
N	0.000000	-1.252873	0.095692
B	0.000000	-2.505782	-0.626111
N	0.000000	-2.464891	-2.063321

N	0.000000	2.464891	-2.063321
B	0.000000	2.505782	-0.626111
N	0.000000	1.252873	0.095692
B	0.000000	1.255956	1.544830
N	0.000000	0.000000	2.233563
B	0.000000	-1.255956	1.544830
N	0.000000	3.735114	0.117389
B	0.000000	3.766094	1.541645
N	0.000000	2.519794	2.230990
N	0.000000	-2.519794	2.230990
B	0.000000	-3.766094	1.541645
N	0.000000	-3.735114	0.117389
H	0.000000	0.000000	3.244089
H	0.000000	4.614809	-0.378140
H	0.000000	4.806937	2.140497
H	0.000000	2.536585	3.240492
H	0.000000	-2.536585	3.240492
H	0.000000	-4.806937	2.140497
H	0.000000	-4.614809	-0.378140
H	0.000000	3.329412	-2.585639
H	0.000000	1.265887	-3.996636
H	0.000000	-1.265887	-3.996636
H	0.000000	-3.329412	-2.585639

Supplementary Table 20 | Nuclear coordinates of S_0 geometry of **2k: bis-BN** at the PBE0/6-31G(d) level of theory.

Element Symbol	*x* (Å)	*y* (Å)	*z* (Å)
N	0.000000	1.244816	-2.924689
B	0.000000	1.253414	-1.470245
N	0.000000	0.000000	-0.750618
B	0.000000	-1.253414	-1.470245
N	0.000000	-1.244816	-2.924689
B	0.000000	0.000000	-3.639557
B	0.000000	0.000000	0.700812
N	0.000000	-1.255642	1.416380
B	0.000000	-2.513839	0.702514
N	0.000000	-2.516616	-0.758478
N	0.000000	2.516616	-0.758478
B	0.000000	2.513839	0.702514
N	0.000000	1.255642	1.416380
B	0.000000	1.254970	2.869491
N	0.000000	0.000000	3.554601
B	0.000000	-1.254970	2.869491
B	0.000000	-3.740532	-1.510243
N	0.000000	-3.698312	-2.932618
B	0.000000	-2.479202	-3.664983
B	0.000000	2.479202	-3.664983
N	0.000000	3.698312	-2.932618
B	0.000000	3.740532	-1.510243

N	0.000000	3.726612	1.464587
B	0.000000	3.756525	2.887375
N	0.000000	2.508140	3.568996
N	0.000000	-2.508140	3.568996
B	0.000000	-3.756525	2.887375
N	0.000000	-3.726612	1.464587
H	0.000000	0.000000	-4.836617
H	0.000000	0.000000	4.565035
H	0.000000	-4.814928	-0.974003
H	0.000000	-4.570780	-3.440527
H	0.000000	-2.480564	-4.862474
H	0.000000	2.480564	-4.862474
H	0.000000	4.570780	-3.440527
H	0.000000	4.814928	-0.974003
H	0.000000	4.605860	0.969754
H	0.000000	4.795608	3.488609
H	0.000000	2.513493	4.578555
H	0.000000	-2.513493	4.578555
H	0.000000	-4.795608	3.488609
H	0.000000	-4.605860	0.969754

Supplementary Table 21 | Nuclear coordinates of S_0 geometry of **3: (6, 6)NB-BN** at the PBE0/6-31G(d) level of theory.

Element Symbol	*x* (Å)	*y* (Å)	*z* (Å)
B	0.000000	4.159835	0.020985
B	-2.066101	3.647817	-1.260611
H	-2.630134	3.535081	-2.316144
B	-2.092197	3.614681	1.222106
B	-3.612333	2.115018	2.491302
H	-4.004971	1.684627	3.539095
B	-3.602523	2.079917	-0.020985
B	-4.192152	0.034613	1.260611
H	-4.376537	-0.510223	2.316144
B	-4.176504	-0.004556	-1.222106
B	-3.602523	-2.079917	0.020985
B	-3.637826	-2.070863	-2.491302
H	-3.461415	-2.626094	-3.539095
B	-2.084307	-3.619236	1.222106
B	-2.126052	-3.613204	-1.260611
H	-1.746402	-4.045303	-2.316144
B	-0.025493	-4.185881	2.491302
H	0.543556	-4.310720	3.539095
B	0.000000	-4.159835	-0.020985
B	2.066101	-3.647817	1.260611
H	2.630134	-3.535081	2.316144
B	2.092197	-3.614681	-1.222106
B	3.602523	-2.079917	0.020985
B	3.612333	-2.115018	-2.491302
H	4.004971	-1.684627	-3.539095

B	4.176504	0.004556	1.222106
B	4.192152	-0.034613	-1.260611
H	4.376537	0.510223	-2.316144
B	3.637826	2.070863	2.491302
H	3.461415	2.626094	3.539095
B	3.602523	2.079917	-0.020985
B	2.126052	3.613204	1.260611
H	1.746402	4.045303	2.316144
B	2.084307	3.619236	-1.222106
B	0.025493	4.185881	-2.491302
H	-0.543556	4.310720	-3.539095
N	-0.802481	4.234757	1.208069
H	-0.318811	4.326200	2.091501
N	-0.714991	4.122390	-1.249745
N	-2.698471	3.214707	-0.039327
N	-2.767815	3.252935	2.444546
H	-2.454721	3.642856	3.322439
N	-3.927590	1.441995	1.249745
N	-4.068648	1.422410	-1.208069
H	-3.906005	1.887001	-2.091501
N	-4.133253	-0.729591	0.039327
N	-4.201032	-0.770530	-2.444546
H	-4.382167	-0.304423	-3.322439
N	-3.212599	-2.680396	-1.249745
N	-3.266167	-2.812347	1.208069
H	-3.587193	-2.439199	2.091501
N	-1.434782	-3.944298	-0.039327
N	-1.433217	-4.023466	2.444546
H	-1.927445	-3.947279	3.322439
N	0.714991	-4.122390	1.249745
N	2.698471	-3.214707	0.039327
N	0.802481	-4.234757	-1.208069
H	0.318811	-4.326200	-2.091501
N	2.767815	-3.252935	-2.444546
H	2.454721	-3.642856	-3.322439
N	3.927590	-1.441995	-1.249745
N	4.068648	-1.422410	1.208069
H	3.906005	-1.887001	2.091501
N	4.201032	0.770530	2.444546
H	4.382167	0.304423	3.322439
N	4.133253	0.729591	-0.039327
N	3.212599	2.680396	1.249745
N	3.266167	2.812347	-1.208069
H	3.587193	2.439199	-2.091501
N	1.434782	3.944298	0.039327
N	1.433217	4.023466	-2.444546
H	1.927445	3.947279	-3.322439

Supplementary Table 22 | Nuclear coordinates of S_0 geometry of **4d: [5]CPP-BN** at the PBE0/6-31G(d) level of theory.

Element Symbol	*x* (Å)	*y* (Å)	*z* (Å)
H	-3.612394	1.059571	2.293103
H	-2.077122	2.988948	-2.096937
H	-3.612394	1.059571	-2.293103
H	3.545288	1.266101	2.293103
H	3.437286	-1.197208	-2.096937
H	-2.124003	-3.108166	-2.293103
H	-2.077122	2.988948	2.096937
H	2.200792	2.899096	-2.096937
H	2.299688	-2.980523	-2.293103
H	-3.484524	-1.051825	2.096937
H	3.437286	-1.197208	2.096937
H	-0.108580	3.763016	-2.293103
H	3.545288	1.266101	-2.293103
H	-0.076432	-3.639011	-2.096937
H	2.200792	2.899096	2.096937
H	-2.124003	-3.108166	2.293103
H	-3.484524	-1.051825	-2.096937
H	-0.076432	-3.639011	2.096937
H	-0.108580	3.763016	2.293103
H	2.299688	-2.980523	2.293103
B	3.157630	-1.440970	0.000000
B	3.459446	0.701409	1.235025
B	3.459446	0.701409	-1.235025
B	2.346206	2.557800	0.000000
B	0.401948	3.506876	1.235025
B	0.401948	3.506876	-1.235025
B	-1.707595	3.021778	0.000000
B	-3.211029	1.465960	1.235025
B	-3.211029	1.465960	-1.235025
B	-3.401557	-0.690239	0.000000
B	-2.386473	-2.600863	1.235025
B	-2.386473	-2.600863	-1.235025
B	-0.394683	-3.448369	0.000000
B	1.736107	-3.073382	1.235025
B	1.736107	-3.073382	-1.235025
N	2.382558	-2.668644	0.000000
N	3.471393	-0.715116	1.209697
N	3.471393	-0.715116	-1.209697
N	3.274282	1.441291	0.000000
N	1.752835	3.080508	1.209697
N	1.752835	3.080508	-1.209697
N	-0.358941	3.559411	0.000000
N	-2.388082	2.618975	1.209697
N	-2.388082	2.618975	-1.209697
N	-3.496119	0.758546	0.000000
N	-3.228751	-1.461893	1.209697

N	-3.228751	-1.461893	-1.209697
N	-1.801780	-3.090604	0.000000
N	0.392604	-3.522474	1.209697
N	0.392604	-3.522474	-1.209697

Supplementary Table 23 | Nuclear coordinates of S_0 geometry of **4e: [6]CPP-BN** at the PBE0/6-31G(d) level of theory.

Element Symbol	*x* (Å)	*y* (Å)	*z* (Å)
H	1.016336	-4.308008	2.295261
H	-1.016497	4.308284	2.295210
H	-4.155470	-1.185559	2.096134
H	-3.104637	3.006249	2.096087
H	3.104459	-3.005963	2.096134
H	-3.222676	-3.034177	-2.295261
H	3.222676	3.034177	2.295261
H	-4.239012	1.273831	2.295261
H	1.051011	4.191522	2.096134
H	-1.051011	-4.191522	-2.096134
H	4.239332	-1.273829	2.295210
H	4.155806	1.185570	2.096087
H	1.016497	-4.308284	-2.295210
H	-1.016336	4.308008	-2.295261
H	-4.155806	-1.185570	-2.096087
H	-3.104459	3.005963	-2.096134
H	3.104637	-3.006249	-2.096087
H	-3.222835	-3.034455	2.295210
H	3.222835	3.034455	-2.295210
H	-4.239332	1.273829	-2.295210
H	1.051169	4.191818	-2.096087
H	-1.051169	-4.191818	2.096087
H	4.239012	-1.273831	-2.295261
H	4.155470	1.185559	-2.096134
B	-4.167930	0.713122	1.233973
B	-4.168094	0.713114	-1.233933
B	-3.923502	-1.445813	0.000010
B	-2.701622	-3.253118	1.233933
B	-2.701546	-3.252972	-1.233973
B	-0.709640	-4.120759	-0.000010
B	1.466383	-3.966094	1.233973
B	1.466472	-3.966232	-1.233933
B	3.213862	-2.674946	0.000010
B	4.168094	-0.713114	1.233933
B	4.167930	-0.713122	-1.233973
B	3.923502	1.445813	-0.000010
B	2.701546	3.252972	1.233973
B	2.701622	3.253118	-1.233933
B	0.709640	4.120759	0.000010
B	-1.466472	3.966232	1.233933
B	-1.466383	3.966094	-1.233973

B	-3.213862	2.674946	-0.000010
N	-0.742735	4.197997	-0.000003
N	-2.701622	3.272585	1.208964
N	-2.701546	3.272461	-1.209012
N	-4.006940	1.455771	0.000003
N	-4.184807	-0.703378	1.209012
N	-4.184952	-0.703381	-1.208964
N	-3.264204	-2.742226	-0.000003
N	-1.483330	-3.975965	1.208964
N	-1.483261	-3.975838	-1.209012
N	0.742735	-4.197997	0.000003
N	2.701546	-3.272461	1.209012
N	2.701622	-3.272585	-1.208964
N	4.006940	-1.455771	-0.000003
N	4.184952	0.703381	1.208964
N	4.184807	0.703378	-1.209012
N	3.264204	2.742226	0.000003
N	1.483261	3.975838	1.209012
N	1.483330	3.975965	-1.208964

Supplementary Table 24 | Nuclear coordinates of S_0 geometry of **4f: [7]CPP-BN** at the PBE0/6-31G(d) level of theory.

Element Symbol	*x* (Å)	*y* (Å)	*z* (Å)
H	-2.975886	4.029906	2.095244
H	-4.606958	2.190880	2.296590
H	-4.606958	2.190880	-2.296590
H	-2.975886	4.029906	-2.095244
H	-5.006142	0.185964	2.095244
H	-5.006142	0.185964	-2.095244
H	-4.585290	-2.235873	2.296590
H	-4.585290	-2.235873	-2.296590
H	-3.266671	-3.798013	-2.095244
H	-3.266671	-3.798013	2.095244
H	-1.110805	-4.978968	-2.296590
H	-1.110805	-4.978968	2.296590
H	0.932670	-4.922008	2.095244
H	0.932670	-4.922008	-2.095244
H	3.200138	-3.972798	2.296590
H	3.200138	-3.972798	-2.296590
H	4.429691	-2.339631	-2.095244
H	4.429691	-2.339631	2.095244
H	5.101312	0.024970	-2.296590
H	5.101312	0.024970	2.296590
H	4.591065	2.004536	2.095244
H	4.591065	2.004536	-2.095244
H	3.161094	4.003935	2.296590
H	3.161094	4.003935	-2.296590
H	1.295273	4.839247	-2.095244
H	1.295273	4.839247	2.095244

H	-1.159492	4.967856	-2.296590
H	-1.159492	4.967856	2.296590
B	1.559844	4.633100	0.000000
B	-0.603247	4.891398	1.233358
B	-0.603247	4.891398	-1.233358
B	-2.649756	4.108226	0.000000
B	-4.200367	2.578100	1.233358
B	-4.200367	2.578100	-1.233358
B	-4.864036	0.489774	0.000000
B	-4.634526	-1.676561	1.233358
B	-4.634526	-1.676561	-1.233358
B	-3.415598	-3.497488	0.000000
B	-1.578792	-4.668737	1.233358
B	-1.578792	-4.668737	-1.233358
B	0.604856	-4.851070	0.000000
B	2.665805	-4.145259	1.233358
B	2.665805	-4.145259	-1.233358
B	4.169840	-2.551697	0.000000
B	4.902996	-0.500316	1.233358
B	4.902996	-0.500316	-1.233358
B	4.594850	1.669155	0.000000
B	3.448131	3.521374	1.233358
B	3.448131	3.521374	-1.233358
N	4.318402	2.403839	1.208515
N	4.318402	2.403839	-1.208515
N	2.884675	4.029281	0.000000
N	0.813083	4.875032	1.208515
N	0.813083	4.875032	-1.208515
N	-1.351653	4.767545	0.000000
N	-3.304505	3.675226	1.208515
N	-3.304505	3.675226	-1.208515
N	-4.570159	1.915751	0.000000
N	-4.933733	-0.292100	1.208515
N	-4.933733	-0.292100	-1.208515
N	-4.347242	-2.378643	0.000000
N	-2.847759	-4.039469	1.208515
N	-2.847759	-4.039469	-1.208515
N	-0.850763	-4.881870	0.000000
N	1.382635	-4.745036	1.208515
N	1.382635	-4.745036	-1.208515
N	3.286358	-3.708950	0.000000
N	4.571877	-1.877494	1.208515
N	4.571877	-1.877494	-1.208515
N	4.948784	0.256886	0.000000

Supplementary Table 25 | Nuclear coordinates of S_0 geometry of **4g: [8]CPP-BN** at the PBE0/6-31G(d) level of theory.

Element Symbol	*x* (Å)	*y* (Å)	*z* (Å)
H	3.984412	4.075623	2.095128
H	5.397961	2.067752	2.297733
H	5.397961	2.067752	-2.297733
H	3.984412	4.075623	-2.095128
H	5.699305	0.064496	2.095128
H	5.699305	0.064496	-2.095128
H	5.279056	-2.354813	2.297733
H	5.279056	-2.354813	-2.297733
H	2.354813	5.279056	-2.297733
H	2.354813	5.279056	2.297733
H	-0.064496	5.699305	-2.095128
H	-0.064496	5.699305	2.095128
H	4.075623	-3.984412	-2.095128
H	4.075623	-3.984412	2.095128
H	2.067752	-5.397961	-2.297733
H	2.067752	-5.397961	2.297733
H	0.064496	-5.699305	2.095128
H	0.064496	-5.699305	-2.095128
H	-2.354813	-5.279056	2.297733
H	-2.354813	-5.279056	-2.297733
H	-3.984412	-4.075623	-2.095128
H	-3.984412	-4.075623	2.095128
H	-5.397961	-2.067752	-2.297733
H	-5.397961	-2.067752	2.297733
H	-5.699305	-0.064496	2.095128
H	-5.699305	-0.064496	-2.095128
H	-5.279056	2.354813	2.297733
H	-5.279056	2.354813	-2.297733
H	-4.075623	3.984412	-2.095128
H	-4.075623	3.984412	2.095128
H	-2.067752	5.397961	-2.297733
H	-2.067752	5.397961	2.297733
B	-0.368775	5.581731	0.000000
B	1.799343	5.333891	-1.232985
B	1.799343	5.333891	1.232985
B	3.686117	4.207643	0.000000
B	5.043958	2.499303	-1.232985
B	5.043958	2.499303	1.232985
B	5.581731	0.368775	0.000000
B	5.333891	-1.799343	-1.232985
B	5.333891	-1.799343	1.232985
B	4.207643	-3.686117	0.000000
B	2.499303	-5.043958	-1.232985
B	2.499303	-5.043958	1.232985
B	0.368775	-5.581731	0.000000
B	-1.799343	-5.333891	-1.232985

B	-1.799343	-5.333891	1.232985
B	-3.686117	-4.207643	0.000000
B	-5.043958	-2.499303	-1.232985
B	-5.043958	-2.499303	1.232985
B	-5.581731	-0.368775	0.000000
B	-5.333891	1.799343	-1.232985
B	-5.333891	1.799343	1.232985
B	-4.207643	3.686117	0.000000
B	-2.499303	5.043958	-1.232985
B	-2.499303	5.043958	1.232985
N	-3.686559	4.270902	-1.208298
N	-3.686559	4.270902	1.208298
N	-1.808015	5.353124	0.000000
N	0.413193	5.626775	-1.208298
N	0.413193	5.626775	1.208298
N	2.506771	5.063690	0.000000
N	4.270902	3.686559	-1.208298
N	4.270902	3.686559	1.208298
N	5.353124	1.808015	0.000000
N	5.626775	-0.413193	-1.208298
N	5.626775	-0.413193	1.208298
N	5.063690	-2.506771	0.000000
N	3.686559	-4.270902	-1.208298
N	3.686559	-4.270902	1.208298
N	1.808015	-5.353124	0.000000
N	-0.413193	-5.626775	-1.208298
N	-0.413193	-5.626775	1.208298
N	-2.506771	-5.063690	0.000000
N	-4.270902	-3.686559	-1.208298
N	-4.270902	-3.686559	1.208298
N	-5.353124	-1.808015	0.000000
N	-5.626775	0.413193	-1.208298
N	-5.626775	0.413193	1.208298
N	-5.063690	2.506771	0.000000

Supplementary Table 26 | Nuclear coordinates of S_0 geometry of **4h: [9]CPP-BN** at the PBE0/6-31G(d) level of theory.

Element Symbol	*x* (Å)	*y* (Å)	*z* (Å)
H	6.108665	-2.118352	2.298672
H	4.815232	-4.203614	2.094922
H	4.815232	-4.203614	-2.094922
H	6.108665	-2.118352	-2.298672
H	3.317858	-5.549326	2.298672
H	0.986650	-6.315327	2.094922
H	0.986650	-6.315327	-2.094922
H	3.317858	-5.549326	-2.298672
H	-1.025411	-6.383709	2.298672
H	-3.303596	-5.472027	2.094922
H	-3.303596	-5.472027	-2.094922

H	-1.025411	-6.383709	-2.298672
H	-4.888879	-4.231083	2.298672
H	-6.048053	-2.068306	2.094922
H	-6.048053	-2.068306	-2.094922
H	-4.888879	-4.231083	-2.298672
H	-6.464786	-0.098687	2.298672
H	-5.962558	2.303199	2.094922
H	-5.962558	2.303199	-2.094922
H	-6.464786	-0.098687	-2.298672
H	-5.015748	4.079886	2.298672
H	-3.087117	5.597012	2.094922
H	-3.087117	5.597012	-2.094922
H	-5.015748	4.079886	-2.298672
H	-1.219786	6.349435	2.298672
H	1.232821	6.271920	2.094922
H	1.232821	6.271920	-2.094922
H	-1.219786	6.349435	-2.298672
H	3.146928	5.648013	2.298672
H	4.975908	4.012127	2.094922
H	4.975908	4.012127	-2.094922
H	3.146928	5.648013	-2.298672
H	6.041159	2.303822	2.298672
H	6.390713	-0.124984	2.094922
H	6.390713	-0.124984	-2.094922
H	6.041159	2.303822	-2.298672
B	-6.051076	-1.748791	0.000000
B	-5.118307	-3.724308	1.232725
B	-5.118307	-3.724308	-1.232725
B	-3.511292	-5.229208	0.000000
B	-1.526912	-6.142970	1.232725
B	-1.526912	-6.142970	-1.232725
B	0.671465	-6.262821	0.000000
B	2.778942	-5.687268	1.232725
B	2.778942	-5.687268	-1.232725
B	4.540036	-4.365990	0.000000
B	5.784499	-2.570430	1.232725
B	5.784499	-2.570430	-1.232725
B	6.284273	-0.426263	0.000000
B	6.083424	1.749140	1.232725
B	6.083424	1.749140	-1.232725
B	5.088029	3.712916	0.000000
B	3.535847	5.250269	1.232725
B	3.535847	5.250269	-1.232725
B	1.511040	6.114781	0.000000
B	-0.666191	6.294738	1.232725
B	-0.666191	6.294738	-1.232725
B	-2.772982	5.655472	0.000000
B	-4.556512	4.393829	1.232725
B	-4.556512	4.393829	-1.232725
B	-5.759494	2.549905	0.000000

B	-6.314790	0.436999	1.232725
B	-6.314790	0.436999	-1.232725
N	-6.070625	1.832264	1.208189
N	-6.070625	1.832264	-1.208189
N	-6.339753	-0.319199	0.000000
N	-5.828125	-2.498526	1.208189
N	-5.828125	-2.498526	-1.208189
N	-4.651356	-4.319636	0.000000
N	-2.858581	-5.660229	1.208189
N	-2.858581	-5.660229	-1.208189
N	-0.786537	-6.298867	0.000000
N	1.448525	-6.173447	1.208189
N	1.448525	-6.173447	-1.208189
N	3.446311	-5.330788	0.000000
N	5.077850	-3.798041	1.208189
N	5.077850	-3.798041	-1.208189
N	6.066592	-1.868374	0.000000
N	6.331192	0.354511	1.208189
N	6.331192	0.354511	-1.208189
N	5.848247	2.468272	0.000000
N	4.622100	4.341183	1.208189
N	4.622100	4.341183	-1.208189
N	2.893442	5.649987	0.000000
N	0.750275	6.296568	1.208189
N	0.750275	6.296568	-1.208189
N	-1.415236	6.188010	0.000000
N	-3.472611	5.305718	1.208189
N	-3.472611	5.305718	-1.208189
N	-5.061710	3.830594	0.000000

Supplementary Table 27 | Nuclear coordinates of S_0 geometry of **4i: [10]CPP-BN** at the PBE0/6-31G(d) level of theory.

Element Symbol	*x* (Å)	*y* (Å)	*z* (Å)
H	6.575181	-2.643570	2.094669
H	7.147899	-0.258765	2.299073
H	7.147899	-0.258765	-2.299073
H	6.575181	-2.643570	-2.094669
H	6.873285	1.726102	2.094669
H	6.873285	1.726102	-2.094669
H	5.934870	3.992084	2.299073
H	5.934870	3.992084	-2.299073
H	5.630673	-4.410775	-2.299073
H	5.630673	-4.410775	2.299073
H	3.765582	-6.003488	-2.094669
H	3.765582	-6.003488	2.094669
H	4.546027	5.436461	-2.094669
H	4.546027	5.436461	2.094669
H	2.454922	6.718093	-2.299073
H	2.454922	6.718093	2.299073

H	0.482342	7.070277	2.094669
H	0.482342	7.070277	-2.094669
H	-1.962722	6.878018	2.299073
H	-1.962722	6.878018	-2.299073
H	-3.765582	6.003488	-2.094669
H	-3.765582	6.003488	2.094669
H	-5.630673	4.410775	-2.299073
H	-5.630673	4.410775	2.299073
H	-6.575181	2.643570	2.094669
H	-6.575181	2.643570	-2.094669
H	-7.147899	0.258765	2.299073
H	-7.147899	0.258765	-2.299073
H	-6.873285	-1.726102	-2.094669
H	-6.873285	-1.726102	2.094669
H	-5.934870	-3.992084	-2.299073
H	-5.934870	-3.992084	2.299073
H	-4.546027	-5.436461	2.094669
H	-4.546027	-5.436461	-2.094669
H	-2.454922	-6.718093	2.299073
H	-2.454922	-6.718093	-2.299073
H	-0.482342	-7.070277	-2.094669
H	-0.482342	-7.070277	2.094669
H	1.962722	-6.878018	-2.299073
H	1.962722	-6.878018	2.299073
B	0.777624	6.960260	0.000000
B	-1.407968	6.887819	1.232555
B	-1.407968	6.887819	-1.232555
B	-3.462027	6.088045	0.000000
B	-5.187629	4.744780	1.232555
B	-5.187629	4.744780	-1.232555
B	-6.379302	2.890403	0.000000
B	-6.985791	0.789396	1.232555
B	-6.985791	0.789396	-1.232555
B	-6.859900	-1.411274	0.000000
B	-6.115619	-3.467511	1.232555
B	-6.115619	-3.467511	-1.232555
B	-4.720250	-5.173893	0.000000
B	-2.909488	-6.399946	1.232555
B	-2.909488	-6.399946	-1.232555
B	-0.777624	-6.960260	0.000000
B	1.407968	-6.887819	1.232555
B	1.407968	-6.887819	-1.232555
B	3.462027	-6.088045	0.000000
B	5.187629	-4.744780	1.232555
B	5.187629	-4.744780	-1.232555
B	6.379302	-2.890403	0.000000
B	6.985791	-0.789396	1.232555
B	6.985791	-0.789396	-1.232555
B	6.859900	1.411274	0.000000
B	6.115619	3.467511	1.232555

B	6.115619	3.467511	-1.232555
B	4.720250	5.173893	0.000000
B	2.909488	6.399946	1.232555
B	2.909488	6.399946	-1.232555
N	4.139167	5.697074	1.207930
N	4.139167	5.697074	-1.207930
N	2.210929	6.690474	0.000000
N	0.000000	7.041971	1.207930
N	0.000000	7.041971	-1.207930
N	-2.143883	6.712259	0.000000
N	-4.139167	5.697074	1.207930
N	-4.139167	5.697074	-1.207930
N	-5.679804	4.170189	0.000000
N	-6.697313	2.176089	1.207930
N	-6.697313	2.176089	-1.207930
N	-7.046234	0.035249	0.000000
N	-6.697313	-2.176089	1.207930
N	-6.697313	-2.176089	-1.207930
N	-5.721241	-4.113156	0.000000
N	-4.139167	-5.697074	1.207930
N	-4.139167	-5.697074	-1.207930
N	-2.210929	-6.690474	0.000000
N	0.000000	-7.041971	1.207930
N	0.000000	-7.041971	-1.207930
N	2.143883	-6.712259	0.000000
N	4.139167	-5.697074	1.207930
N	4.139167	-5.697074	-1.207930
N	5.679804	-4.170189	0.000000
N	6.697313	-2.176089	1.207930
N	6.697313	-2.176089	-1.207930
N	7.046234	-0.035249	0.000000
N	6.697313	2.176089	1.207930
N	6.697313	2.176089	-1.207930
N	5.721241	4.113156	0.000000

Supplementary Table 28 | Nuclear coordinates of S_0 geometry of **4j: [11]CPP-BN** at the PBE0/6-31G(d) level of theory.

Element Symbol	*x* (Å)	*y* (Å)	*z* (Å)
H	4.192075	-6.559129	2.094461
H	1.970040	-7.595255	2.299681
H	1.970040	-7.595255	-2.299681
H	4.192075	-6.559129	-2.094461
H	-0.018441	-7.785790	2.094493
H	-2.447619	-7.455103	2.299713
H	-2.447619	-7.455103	-2.299713
H	-0.018441	-7.785790	-2.094493
H	-4.223015	-6.539279	2.094462
H	-6.087023	-4.946709	2.299660
H	-6.087023	-4.946709	-2.299660
H	-4.223015	-6.539279	-2.094462

H	-7.085508	-3.216574	2.094475
H	-7.793925	-0.869484	2.299663
H	-7.793925	-0.869484	-2.299663
H	-7.085508	-3.216574	-2.094475
H	-7.698630	1.125868	2.094489
H	-7.026152	3.483481	2.299712
H	-7.026152	3.483481	-2.299712
H	-7.698630	1.125868	-2.094489
H	-5.869253	5.111871	2.094463
H	-4.029409	6.732443	2.299652
H	-4.029409	6.732443	-2.299652
H	-5.869253	5.111871	-2.094463
H	-2.174949	7.475026	2.094506
H	0.248942	7.842698	2.299714
H	0.248942	7.842698	-2.299714
H	-2.174949	7.475026	-2.094506
H	2.210558	7.465335	2.094460
H	4.448036	6.462937	2.299650
H	4.448036	6.462937	-2.299650
H	2.210558	7.465335	-2.094460
H	5.893168	5.083795	2.094507
H	7.233096	3.030704	2.299669
H	7.233096	3.030704	-2.299669
H	5.893168	5.083795	-2.094507
H	7.704130	1.089454	2.094477
H	7.722683	-1.362199	2.299661
H	7.722683	-1.362199	-2.299661
H	7.704130	1.089454	-2.094477
H	7.069953	-3.250075	2.094507
H	5.761367	-5.323398	2.299690
H	5.761367	-5.323398	-2.299690
H	7.069953	-3.250075	-2.094507
B	5.634497	5.257816	0.000000
B	6.904600	3.476050	-1.232463
B	6.904600	3.476050	1.232463
B	7.580254	1.375643	0.000000
B	7.686648	-0.809937	-1.232453
B	7.686648	-0.809937	1.232453
B	7.120491	-2.942520	0.000000
B	6.029647	-4.839306	-1.232484
B	6.029647	-4.839306	1.232484
B	4.400965	-6.327760	0.000000
B	2.457462	-7.333192	-1.232466
B	2.457462	-7.333192	1.232466
B	0.282459	-7.703876	0.000000
B	-1.895981	-7.498339	-1.232471
B	-1.895981	-7.498339	1.232471
B	-3.925774	-6.633499	0.000000
B	-5.646359	-5.281521	-1.232424
B	-5.646359	-5.281521	1.232424

B	-6.886286	-3.456384	0.000000
B	-7.603841	-1.389224	-1.232447
B	-7.603841	-1.389224	1.232447
B	-7.660802	0.816476	0.000000
B	-7.147307	2.943529	-1.232483
B	-7.147307	2.943529	1.232483
B	-6.004108	4.830581	0.000000
B	-4.423018	6.343273	-1.232451
B	-4.423018	6.343273	1.232451
B	-2.440684	7.311962	0.000000
B	-0.292528	7.728596	-1.232467
B	-0.292528	7.728596	1.232467
B	1.898768	7.471603	0.000000
B	3.930811	6.659889	-1.232445
B	3.930811	6.659889	1.232445
N	2.654618	7.274506	-1.207862
N	4.569390	6.255155	0.000000
N	2.654618	7.274506	1.207862
N	6.163577	4.683248	-1.207899
N	7.222851	2.790329	0.000000
N	6.163577	4.683248	1.207899
N	7.715138	0.606255	-1.207875
N	7.584028	-1.558973	0.000000
N	7.715138	0.606255	1.207875
N	6.818459	-3.662794	-1.207917
N	5.538853	-5.414335	0.000000
N	6.818459	-3.662794	1.207917
N	3.757311	-6.770336	-1.207868
N	1.733592	-7.551274	0.000000
N	3.757311	-6.770336	1.207868
N	-0.498252	-7.728121	-1.207878
N	-0.498252	-7.728121	1.207878
N	-2.622736	-7.290169	0.000000
N	-4.595355	-6.231136	-1.207852
N	-4.595355	-6.231136	1.207852
N	-6.144989	-4.713200	0.000000
N	-7.232231	-2.756101	-1.207870
N	-7.232231	-2.756101	1.207870
N	-7.716360	-0.641613	0.000000
N	-7.573319	1.592643	-1.207892
N	-7.573319	1.592643	1.207892
N	-6.838333	3.633452	0.000000
N	-5.511494	5.436840	-1.207859
N	-5.511494	5.436840	1.207859
N	-3.790007	6.756616	0.000000
N	-1.698339	7.554987	-1.207878
N	-1.698339	7.554987	1.207878
N	0.463418	7.734081	0.000000

Supplementary Table 29 | Nuclear coordinates of S_0 geometry of **4k: [12]CPP-BN** at the PBE0/6-31G(d) level of theory.

Element Symbol	*x* (Å)	*y* (Å)	*z* (Å)
H	8.288152	1.995978	2.301855
H	8.458600	-0.448544	2.095496
H	8.480039	-0.446038	-2.093339
H	8.312196	1.999191	-2.298164
H	8.198169	-2.424748	2.298164
H	7.120910	-4.626300	2.093339
H	7.101090	-4.617751	-2.095496
H	8.175739	-2.415509	-2.301855
H	5.872643	-6.179761	2.301855
H	3.840849	-7.549634	2.095496
H	3.853739	-7.566948	-2.093339
H	5.887449	-6.198978	-2.298164
H	1.999191	-8.312196	2.298164
H	-0.446038	-8.480039	2.093339
H	-0.448544	-8.458600	-2.095496
H	1.995978	-8.288152	-2.301855
H	-2.415509	-8.175739	2.301855
H	-4.617751	-7.101090	2.095496
H	-4.626300	-7.120910	-2.093339
H	-2.424748	-8.198169	-2.298164
H	-6.198978	-5.887449	2.298164
H	-7.566948	-3.853739	2.093339
H	-7.549634	-3.840849	-2.095496
H	-6.179761	-5.872643	-2.301855
H	-8.288152	-1.995978	2.301855
H	-8.458600	0.448544	2.095496
H	-8.480039	0.446038	-2.093339
H	-8.312196	-1.999191	-2.298164
H	-8.198169	2.424748	2.298164
H	-7.120910	4.626300	2.093339
H	-7.101090	4.617751	-2.095496
H	-8.175739	2.415509	-2.301855
H	-5.872643	6.179761	2.301855
H	-3.840849	7.549634	2.095496
H	-3.853739	7.566948	-2.093339
H	-5.887449	6.198978	-2.298164
H	-1.999191	8.312196	2.298164
H	0.446038	8.480039	2.093339
H	0.448544	8.458600	-2.095496
H	-1.995978	8.288152	-2.301855
H	2.415509	8.175739	2.301855
H	4.617751	7.101090	2.095496
H	4.626300	7.120910	-2.093339
H	2.424748	8.198169	-2.298164
H	6.198978	5.887449	2.298164
H	7.566948	3.853739	2.093339
H	7.549634	3.840849	-2.095496

H	6.179761	5.872643	-2.301855
B	-8.377445	0.742988	0.000450
B	-8.302623	-1.444500	1.233777
B	-8.314764	-1.445914	-1.230873
B	-7.626574	-3.545276	-0.000450
B	-6.477840	-5.409581	1.230873
B	-6.468032	-5.402285	-1.233777
B	-4.832169	-6.883587	0.000450
B	-2.900338	-7.912532	1.233777
B	-2.905183	-7.923754	-1.230873
B	-0.742988	-8.377445	-0.000450
B	1.445914	-8.314764	1.230873
B	1.444500	-8.302623	-1.233777
B	3.545276	-7.626574	0.000450
B	5.402285	-6.468032	1.233777
B	5.409581	-6.477840	-1.230873
B	6.883587	-4.832169	-0.000450
B	7.923754	-2.905183	1.230873
B	7.912532	-2.900338	-1.233777
B	8.377445	-0.742988	0.000450
B	8.302623	1.444500	1.233777
B	8.314764	1.445914	-1.230873
B	7.626574	3.545276	-0.000450
B	6.477840	5.409581	1.230873
B	6.468032	5.402285	-1.233777
B	4.832169	6.883587	0.000450
B	2.900338	7.912532	1.233777
B	2.905183	7.923754	-1.230873
B	0.742988	8.377445	-0.000450
B	-1.445914	8.314764	1.230873
B	-1.444500	8.302623	-1.233777
B	-3.545276	7.626574	0.000450
B	-5.402285	6.468032	1.233777
B	-5.409581	6.477840	-1.230873
B	-6.883587	4.832169	-0.000450
B	-7.923754	2.905183	1.230873
B	-7.912532	2.900338	-1.233777
N	8.436745	0.034378	1.208974
N	8.157138	2.185699	0.001135
N	8.448559	0.035802	-1.206528
N	8.157138	-2.185699	-0.001135
N	7.334568	-4.193274	1.206528
N	7.323624	-4.188600	-1.208974
N	5.971440	-5.971440	0.001135
N	4.248145	-7.289246	1.208974
N	4.255285	-7.298765	-1.206528
N	7.298765	4.255285	1.206528
N	7.289246	4.248145	-1.208974
N	5.971440	5.971440	-0.001135
N	4.188600	7.323624	1.208974

N	4.193274	7.334568	-1.206528
N	2.185699	8.157138	0.001135
N	-0.035802	8.448559	1.206528
N	-0.034378	8.436745	-1.208974
N	-2.185699	8.157138	-0.001135
N	-4.248145	7.289246	1.208974
N	-4.255285	7.298765	-1.206528
N	-5.971440	5.971440	0.001135
N	-7.334568	4.193274	1.206528
N	-7.323624	4.188600	-1.208974
N	-8.157138	2.185699	-0.001135
N	-8.436745	-0.034378	1.208974
N	-8.448559	-0.035802	-1.206528
N	-8.157138	-2.185699	0.001135
N	-7.298765	-4.255285	1.206528
N	-5.971440	-5.971440	-0.001135
N	-7.289246	-4.248145	-1.208974
N	-4.188600	-7.323624	1.208974
N	-4.193274	-7.334568	-1.206528
N	-2.185699	-8.157138	0.001135
N	0.035802	-8.448559	1.206528
N	0.034378	-8.436745	-1.208974
N	2.185699	-8.157138	-0.001135

Supplementary Table 30 | Nuclear coordinates of S_0 geometry of **5: [6]CMP-BN** at the PBE0/6-31G(d) level of theory.

Element Symbol	*x* (Å)	*y* (Å)	*z* (Å)
H	-2.089838	-5.569200	-0.432099
H	2.089838	-5.569200	-0.432099
H	5.867987	0.974747	-0.432099
H	3.778150	4.594452	-0.432099
H	-3.778150	4.594452	-0.432099
H	-5.867987	0.974747	-0.432099
H	0.000000	6.731134	0.640929
H	-5.829333	-3.365567	0.640929
H	5.829333	-3.365567	0.640929
B	-3.798250	0.734211	-0.064990
B	-2.534971	2.922275	-0.064990
B	-5.009796	2.892407	-0.460167
H	-6.030815	3.481892	-0.686623
B	0.000000	2.981767	-0.057425
H	0.000000	1.806039	-0.300451
B	-1.251519	5.073265	0.327273
H	-2.264117	5.705184	0.453199
B	1.251519	5.073265	0.327273
H	2.264117	5.705184	0.453199
B	2.534971	2.922275	-0.064990
B	3.798250	0.734211	-0.064990
B	5.009796	2.892407	-0.460167
H	6.030815	3.481892	-0.686623

B	5.019336	-1.452785	0.327273
H	6.072893	-0.891809	0.453199
B	3.767817	-3.620480	0.327273
H	3.808776	-4.813375	0.453199
B	2.582286	-1.490883	-0.057425
H	1.564075	-0.903019	-0.300451
B	1.263279	-3.656486	-0.064990
B	0.000000	-5.784814	-0.460167
H	0.000000	-6.963785	-0.686623
B	-1.263279	-3.656486	-0.064990
B	-2.582286	-1.490883	-0.057425
H	-1.564075	-0.903019	-0.300451
B	-3.767817	-3.620480	0.327273
H	-3.808776	-4.813375	0.453199
B	-5.019336	-1.452785	0.327273
H	-6.072893	-0.891809	0.453199
N	-4.997126	1.473455	-0.326739
N	-3.774613	3.590910	-0.326739
N	-1.262186	3.659025	0.069378
N	0.000000	5.739907	0.448639
N	1.262186	3.659025	0.069378
N	3.774613	3.590910	-0.326739
N	4.997126	1.473455	-0.326739
N	3.799902	-0.736427	0.069378
N	4.970905	-2.869954	0.448639
N	2.537715	-2.922598	0.069378
N	1.222513	-5.064366	-0.326739
N	-1.222513	-5.064366	-0.326739
N	-2.537715	-2.922598	0.069378
N	-3.799902	-0.736427	0.069378
N	-4.970905	-2.869954	0.448639
N	2.596902	1.499322	0.066923
H	1.739977	1.004576	0.263187
N	0.000000	-2.998644	0.066923
H	0.000000	-2.009152	0.263187
N	-2.596902	1.499322	0.066923
H	-1.739977	1.004576	0.263187

Supplementary Table 31 | Nuclear coordinates of S_0 geometry of **6a: [2]PP-BN** at the PBE0/6-31G(d) level of theory.

Element Symbol	*x* (Å)	*y* (Å)	*z* (Å)
H	-0.005474	2.091886	-3.399624
H	-0.007067	2.300831	-0.929648
H	0.007067	-2.300831	-0.929648
H	0.005474	-2.091886	-3.399624
H	0.000000	0.000000	-4.861010
H	-0.598852	-2.008249	0.996729
H	0.598852	2.008249	0.996729
H	-0.651047	-2.197371	3.510188

H	0.651047	2.197371	3.510188
H	0.000000	0.000000	4.616350
B	0.000000	0.000000	-3.662169
B	0.001366	1.235429	-1.483622
B	-0.001366	-1.235429	-1.483622
B	0.000000	0.000000	0.730858
B	0.353127	1.197659	2.915890
B	-0.353127	-1.197659	2.915890
N	0.000000	-1.210269	-2.907868
N	0.000000	1.210269	-2.907868
N	0.000000	0.000000	-0.742640
N	0.343947	1.166180	1.491402
N	-0.343947	-1.166180	1.491402
N	0.000000	0.000000	3.607463

Supplementary Table 32 | Nuclear coordinates of S_0 geometry of **6b: [3]PP-BN** at the PBE0/6-31G(d) level of theory.

Element Symbol	*x* (Å)	*y* (Å)	*z* (Å)
H	2.283640	-0.192017	5.713755
H	2.087769	-0.178641	3.200107
H	-2.087769	0.178641	3.200107
H	-2.283640	0.192017	5.713755
H	0.000000	0.000000	6.820172
H	-2.272404	-0.355533	1.259943
H	2.272404	0.355533	1.259943
H	-2.069523	-0.322553	-1.189618
H	2.069523	0.322553	-1.189618
H	-2.296759	0.154902	-3.138972
H	2.296759	-0.154902	-3.138972
H	-2.087306	0.138922	-5.607371
H	2.087306	-0.138922	-5.607371
H	0.000000	0.000000	-7.068127
B	0.000000	0.000000	-5.869669
B	1.233826	-0.078699	-3.691973
B	-1.233826	0.078699	-3.691973
B	0.000000	0.000000	-1.480666
B	1.216747	0.183605	0.712379
B	-1.216747	-0.183605	0.712379
B	0.000000	0.000000	2.932592
B	1.244182	-0.103162	5.118929
B	-1.244182	0.103162	5.118929
N	1.207979	-0.077777	-5.115147
N	-1.207979	0.077777	-5.115147
N	0.000000	0.000000	-2.949954
N	1.193777	0.179729	-0.708301
N	-1.193777	-0.179729	-0.708301
N	0.000000	0.000000	1.462008
N	1.211690	-0.100474	3.695147
N	-1.211690	0.100474	3.695147

N	0.000000	0.000000	5.811352

Supplementary Table 33 | Nuclear coordinates of S_0 geometry of **6c: [4]PP-BN** at the PBE0/6-31G(d) level of theory.

Element Symbol	*x* (Å)	*y* (Å)	*z* (Å)
H	2.293659	-0.174147	3.463673
H	-2.293659	0.174147	3.463673
H	2.088373	-0.157029	1.014863
H	-2.088373	0.157029	1.014863
H	-2.072073	-0.310323	5.404972
H	2.072073	0.310323	5.404972
H	-2.266811	-0.336549	7.918851
H	2.266811	0.336549	7.918851
H	0.000000	0.000000	9.025366
H	-2.296360	-0.150873	-0.949087
H	2.296360	0.150873	-0.949087
H	-2.090129	-0.136140	-3.396314
H	2.090129	0.136140	-3.396314
H	-2.279775	0.321484	-5.346145
H	2.279775	-0.321484	-5.346145
H	-2.071640	0.290542	-7.813932
H	2.071640	-0.290542	-7.813932
H	0.000000	0.000000	-9.274552
B	0.000000	0.000000	-8.076287
B	1.225059	-0.168478	-5.898845
B	-1.225059	0.168478	-5.898845
B	0.000000	0.000000	-3.688131
B	1.229040	0.075842	-1.495363
B	-1.229040	-0.075842	-1.495363
B	0.000000	0.000000	0.721725
B	1.227237	-0.087245	2.916200
B	-1.227237	0.087245	2.916200
B	0.000000	0.000000	5.136893
B	1.235042	0.182011	7.323791
B	-1.235042	-0.182011	7.323791
N	1.199103	-0.165742	-7.321583
N	-1.199103	0.165742	-7.321583
N	0.000000	0.000000	-5.156270
N	1.205076	0.073666	-2.914766
N	-1.205076	-0.073666	-2.914766
N	0.000000	0.000000	-0.744374
N	1.204108	-0.085498	1.496414
N	-1.204108	0.085498	1.496414
N	0.000000	0.000000	3.667122
N	1.202795	0.177325	5.900308
N	-1.202795	-0.177325	5.900308
N	0.000000	0.000000	8.016575

Supplementary Table 34 | Nuclear coordinates of S_0 geometry of **6d: [5]PP-BN** at the PBE0/6-31G(d) level of theory.

Element Symbol	*x* (Å)	*y* (Å)	*z* (Å)
H	2.288497	-0.233110	5.669050
H	-2.288497	0.233110	5.669050
H	2.083467	-0.210210	3.220465
H	-2.083467	0.210210	3.220465
H	-2.080844	-0.243657	7.610754
H	2.080844	0.243657	7.610754
H	-2.276408	-0.263783	10.124803
H	2.276408	0.263783	10.124803
H	0.000000	0.000000	11.231334
H	-2.301672	0.010648	1.254428
H	2.301672	-0.010648	1.254428
H	-2.094277	0.011416	-1.191476
H	2.094277	-0.011416	-1.191476
H	2.292061	0.211279	-3.156780
H	-2.292061	-0.211279	-3.156780
H	2.085770	0.191452	-5.602819
H	-2.085770	-0.191452	-5.602819
H	-2.290184	0.237883	-7.553100
H	2.290184	-0.237883	-7.553100
H	-2.080882	0.214487	-10.020568
H	2.080882	-0.214487	-10.020568
H	0.000000	0.000000	-11.481119
B	0.000000	0.000000	-10.282950
B	1.230490	-0.123677	-8.105628
B	-1.230490	0.123677	-8.105628
B	0.000000	0.000000	-5.895137
B	1.226692	0.109052	-3.702371
B	-1.226692	-0.109052	-3.702371
B	0.000000	0.000000	-1.485633
B	1.231103	-0.007146	0.708671
B	-1.231103	0.007146	0.708671
B	0.000000	0.000000	2.926458
B	1.224451	-0.119181	5.121583
B	-1.224451	0.119181	5.121583
B	0.000000	0.000000	7.342468
B	1.240209	0.142391	9.529604
B	-1.240209	-0.142391	9.529604
N	1.204376	-0.121801	-9.528144
N	-1.204376	0.121801	-9.528144
N	0.000000	0.000000	-7.362756
N	1.202608	0.106195	-5.121237
N	-1.202608	-0.106195	-5.121237
N	0.000000	0.000000	-2.950536
N	1.207156	-0.006442	-0.709772
N	-1.207156	0.006442	-0.709772
N	0.000000	0.000000	1.461211

N	1.201347	-0.116884	3.702197
N	-1.201347	0.116884	3.702197
N	0.000000	0.000000	5.873101
N	1.207842	0.138719	8.106284
N	-1.207842	-0.138719	8.106284
N	0.000000	0.000000	10.222560

Supplementary Table 35 | Nuclear coordinates of S_0 geometry of **6e: [6]PP-BN** at the PBE0/6-31G(d) level of theory.

Element Symbol	*x* (Å)	*y* (Å)	*z* (Å)
H	2.286641	-0.251220	7.874720
H	-2.286641	0.251220	7.874720
H	2.081603	-0.226782	5.425939
H	-2.081603	0.226782	5.425939
H	-2.083122	-0.222826	9.816408
H	2.083122	0.222826	9.816408
H	-2.278919	-0.241057	12.330560
H	2.278919	0.241057	12.330560
H	0.000000	0.000000	13.437080
H	-2.301537	0.026495	3.460254
H	2.301537	-0.026495	3.460254
H	-2.093932	0.025416	1.014136
H	2.093932	-0.025416	1.014136
H	2.301912	0.030551	-0.952763
H	-2.301912	-0.030551	-0.952763
H	2.094115	0.027274	-3.397694
H	-2.094115	-0.027274	-3.397694
H	-2.291647	-0.217338	-5.363323
H	2.291647	0.217338	-5.363323
H	-2.085245	-0.197134	-7.808992
H	2.085245	0.197134	-7.808992
H	-2.292299	0.217700	-9.759621
H	2.292299	-0.217700	-9.759621
H	-2.082689	0.196085	-12.226856
H	2.082689	-0.196085	-12.226856
H	0.000000	0.000000	-13.687385
B	0.000000	0.000000	-12.489268
B	1.231562	-0.112906	-10.311998
B	-1.231562	0.112906	-10.311998
B	0.000000	0.000000	-8.101584
B	1.226444	0.112731	-5.908793
B	-1.226444	-0.112731	-5.908793
B	0.000000	0.000000	-3.692312
B	1.231205	0.016377	-1.498024
B	-1.231205	-0.016377	-1.498024
B	0.000000	0.000000	0.719290
B	1.231006	-0.016228	2.914169
B	-1.231006	0.016228	2.914169
B	0.000000	0.000000	5.131890

B	1.223504	-0.128917	7.327162
B	-1.223504	0.128917	7.327162
B	0.000000	0.000000	9.548035
B	1.241560	0.130021	11.735281
B	-1.241560	-0.130021	11.735281
N	1.205402	-0.111201	-11.734399
N	-1.205402	0.111201	-11.734399
N	0.000000	0.000000	-9.568946
N	1.202278	0.109798	-7.327403
N	-1.202278	-0.109798	-7.327403
N	0.000000	0.000000	-5.156621
N	1.207052	0.016499	-2.915916
N	-1.207052	-0.016499	-2.915916
N	0.000000	0.000000	-0.744697
N	1.207017	-0.015595	1.496135
N	-1.207017	0.015595	1.496135
N	0.000000	0.000000	3.667161
N	1.200391	-0.126515	5.907967
N	-1.200391	0.126515	5.907967
N	0.000000	0.000000	8.078875
N	1.209166	0.126680	10.312041
N	-1.209166	-0.126680	10.312041
N	0.000000	0.000000	12.428314

Supplementary Table 36 | Nuclear coordinates of S_0 geometry of **6f: [7]PP-BN** at the PBE0/6-31G(d) level of theory.

Element Symbol	*x* (Å)	*y* (Å)	*z* (Å)
H	2.300478	0.017869	10.080106
H	-2.300478	-0.017869	10.080106
H	2.093778	0.018773	7.632306
H	-2.093778	-0.018773	7.632306
H	-2.048509	-0.438871	12.023292
H	2.048509	0.438871	12.023292
H	-2.241277	-0.477614	14.537251
H	2.241277	0.477614	14.537251
H	0.000000	0.000000	15.643909
H	-2.301530	0.046961	5.664642
H	2.301530	-0.046961	5.664642
H	-2.093486	0.042488	3.219318
H	2.093486	-0.042488	3.219318
H	2.302129	-0.010952	1.252624
H	-2.302129	0.010952	1.252624
H	2.094013	-0.010215	-1.192725
H	-2.094013	0.010215	-1.192725
H	-2.299812	0.102980	-3.158849
H	2.299812	-0.102980	-3.158849
H	-2.092194	0.093480	-5.604039
H	2.092194	-0.093480	-5.604039
H	2.301574	0.047218	-7.569862

H	-2.301574	-0.047218	-7.569862
H	2.094103	0.042347	-10.015282
H	-2.094103	-0.042347	-10.015282
H	-2.269491	0.389153	-11.965780
H	2.269491	-0.389153	-11.965780
H	-2.062062	0.352008	-14.433091
H	2.062062	-0.352008	-14.433091
H	0.000000	0.000000	-15.893563
B	0.000000	0.000000	-14.695473
B	1.219661	-0.205046	-12.518246
B	-1.219661	0.205046	-12.518246
B	0.000000	0.000000	-10.307966
B	1.231458	0.022065	-8.115176
B	-1.231458	-0.022065	-8.115176
B	0.000000	0.000000	-5.898740
B	1.230334	-0.053557	-3.704490
B	-1.230334	0.053557	-3.704490
B	0.000000	0.000000	-1.487731
B	1.231357	-0.006594	0.707015
B	-1.231357	0.006594	0.707015
B	0.000000	0.000000	2.924196
B	1.230766	-0.024193	5.119312
B	-1.230766	0.024193	5.119312
B	0.000000	0.000000	7.337518
B	1.230002	0.013843	9.533159
B	-1.230002	-0.013843	9.533159
B	0.000000	0.000000	11.754493
B	1.221119	0.258973	13.941976
B	-1.221119	-0.258973	13.941976
N	1.193649	-0.201461	-13.940590
N	-1.193649	0.201461	-13.940590
N	0.000000	0.000000	-11.775160
N	1.207102	0.020794	-9.533647
N	-1.207102	-0.020794	-9.533647
N	0.000000	0.000000	-7.362798
N	1.206082	-0.052341	-5.122123
N	-1.206082	0.052341	-5.122123
N	0.000000	0.000000	-2.951028
N	1.207147	-0.006652	-0.710453
N	-1.207147	0.006652	-0.710453
N	0.000000	0.000000	1.460749
N	1.206805	-0.023765	3.701512
N	-1.206805	0.023765	3.701512
N	0.000000	0.000000	5.872887
N	1.206797	0.013163	8.114115
N	-1.206797	-0.013163	8.114115
N	0.000000	0.000000	10.285392
N	1.189244	0.252296	12.518795
N	-1.189244	-0.252296	12.518795
N	0.000000	0.000000	14.635151

Supplementary Table 37 | Nuclear coordinates of S_0 geometry of **6g: [8]PP-BN** at the PBE0/6-31G(d) level of theory.

Element Symbol	*x* (Å)	*y* (Å)	*z* (Å)
H	-2.297734	0.113511	12.285475
H	-2.091378	0.101284	9.837203
H	2.091378	-0.101284	9.837203
H	2.297734	-0.113511	12.285475
H	-2.301059	-0.060634	7.870917
H	2.301059	0.060634	7.870917
H	-2.093179	-0.054081	5.424747
H	2.093179	0.054081	5.424747
H	2.069220	0.327497	14.228093
H	-2.069220	-0.327497	14.228093
H	2.263834	0.355743	16.742163
H	-2.263834	-0.355743	16.742163
H	0.000000	0.000000	17.848784
H	2.302040	-0.021382	3.458646
H	-2.302040	0.021382	3.458646
H	2.093839	-0.019091	1.012926
H	-2.093839	0.019091	1.012926
H	-2.302058	-0.026091	-0.953022
H	2.302058	0.026091	-0.953022
H	-2.093901	-0.023519	-3.398549
H	2.093901	0.023519	-3.398549
H	2.301473	-0.056174	-5.364421
H	-2.301473	0.056174	-5.364421
H	2.093659	-0.051012	-7.809635
H	-2.093659	0.051012	-7.809635
H	-2.300312	-0.090232	-9.775326
H	2.300312	0.090232	-9.775326
H	-2.092928	-0.081563	-12.220835
H	2.092928	0.081563	-12.220835
H	2.275790	-0.350321	-14.171002
H	-2.275790	0.350321	-14.171002
H	2.067763	-0.316795	-16.638471
H	-2.067763	0.316795	-16.638471
H	0.000000	0.000000	-18.098871
B	0.000000	0.000000	-16.900795
B	-1.223035	0.184175	-14.723606
B	1.223035	-0.184175	-14.723606
B	0.000000	0.000000	-12.513451
B	-1.230884	-0.045123	-10.320702
B	1.230884	0.045123	-10.320702
B	0.000000	0.000000	-8.104379
B	-1.231240	0.028663	-5.910145
B	1.231240	-0.028663	-5.910145
B	0.000000	0.000000	-3.693576
B	-1.231478	-0.013154	-1.498893
B	1.231478	0.013154	-1.498893

B	0.000000	0.000000	0.717831
B	-1.231385	0.010430	2.912747
B	1.231385	-0.010430	2.912747
B	0.000000	0.000000	5.129814
B	-1.230771	-0.030338	7.324921
B	1.230771	0.030338	7.324921
B	0.000000	0.000000	9.542740
B	-1.228922	0.055537	11.738278
B	1.228922	-0.055537	11.738278
B	0.000000	0.000000	13.959427
B	-1.233359	-0.192561	16.146855
B	1.233359	0.192561	16.146855
N	-1.196938	0.181064	-16.145919
N	1.196938	-0.181064	-16.145919
N	0.000000	0.000000	-13.980549
N	-1.206525	-0.043422	-11.739108
N	1.206525	0.043422	-11.739108
N	0.000000	0.000000	-9.568288
N	-1.206916	0.027984	-7.327655
N	1.206916	-0.027984	-7.327655
N	0.000000	0.000000	-5.156584
N	-1.207158	-0.012778	-2.916122
N	1.207158	0.012778	-2.916122
N	0.000000	0.000000	-0.745012
N	-1.207145	0.010253	1.495463
N	1.207145	-0.010253	1.495463
N	0.000000	0.000000	3.666604
N	-1.206765	-0.029675	5.907199
N	1.206765	0.029675	5.907199
N	0.000000	0.000000	8.078317
N	-1.205729	0.054556	10.319260
N	1.205729	-0.054556	10.319260
N	0.000000	0.000000	12.490385
N	-1.201176	-0.187606	14.723693
N	1.201176	0.187606	14.723693
N	0.000000	0.000000	16.840028

Supplementary Table 38 | Nuclear coordinates of S_0 geometry of **6h: [9]PP-BN** at the PBE0/6-31G(d) level of theory.

Element Symbol	*x* (Å)	*y* (Å)	*z* (Å)
H	2.297661	0.114236	14.491387
H	2.091330	0.101879	12.042887
H	-2.091330	-0.101879	12.042887
H	-2.297661	-0.114236	14.491387
H	2.300939	-0.064712	10.076780
H	-2.300939	0.064712	10.076780
H	2.093052	-0.057690	7.630518
H	-2.093052	0.057690	7.630518
H	-2.068490	0.331944	16.433661

H	2.068490	-0.331944	16.433661
H	-2.263066	0.360607	18.947826
H	2.263066	-0.360607	18.947826
H	0.000000	0.000000	20.054405
H	-2.302119	0.017243	5.664354
H	2.302119	-0.017243	5.664354
H	-2.093796	0.015988	3.218597
H	2.093796	-0.015988	3.218597
H	2.302157	-0.019650	1.252798
H	-2.302157	0.019650	1.252798
H	2.093832	-0.017833	-1.192950
H	-2.093832	0.017833	-1.192950
H	-2.302216	0.010995	-3.158663
H	2.302216	-0.010995	-3.158663
H	-2.093996	0.010029	-5.604207
H	2.093996	-0.010029	-5.604207
H	2.302182	-0.008646	-7.570285
H	-2.302182	0.008646	-7.570285
H	2.094263	-0.007679	-10.015198
H	-2.094263	0.007679	-10.015198
H	-2.302102	0.024370	-11.981888
H	2.302102	-0.024370	-11.981888
H	-2.094385	0.022279	-14.426772
H	2.094385	-0.022279	-14.426772
B	0.000000	0.000000	-14.719655
B	1.231575	-0.011293	-12.526808
B	-1.231575	0.011293	-12.526808
B	0.000000	0.000000	-10.310241
B	1.231533	-0.004684	-8.115898
B	-1.231533	0.004684	-8.115898
B	0.000000	0.000000	-5.899307
B	1.231603	-0.005959	-3.704622
B	-1.231603	0.005959	-3.704622
B	0.000000	0.000000	-1.488087
B	1.231542	-0.010455	0.706776
B	-1.231542	0.010455	0.706776
B	0.000000	0.000000	2.923445
B	1.231401	-0.009709	5.118451
B	-1.231401	0.009709	5.118451
B	0.000000	0.000000	7.335531
B	1.230725	-0.032583	9.530696
B	-1.230725	0.032583	9.530696
B	0.000000	0.000000	11.748477
B	1.228946	0.055855	13.944015
B	-1.228946	-0.055855	13.944015
B	0.000000	0.000000	16.165065
B	1.232959	-0.195199	18.352482
B	-1.232959	0.195199	18.352482
N	1.207190	-0.010103	-13.945148
N	-1.207190	0.010103	-13.945148

N	0.000000	0.000000	-11.774116
N	1.207181	-0.004541	-9.533316
N	-1.207181	0.004541	-9.533316
N	0.000000	0.000000	-7.362151
N	1.207227	-0.005811	-5.121708
N	-1.207227	0.005811	-5.121708
N	0.000000	0.000000	-2.950614
N	1.207194	-0.010210	-0.710247
N	-1.207194	0.010210	-0.710247
N	0.000000	0.000000	1.460853
N	1.207153	-0.009486	3.701278
N	-1.207153	0.009486	3.701278
N	0.000000	0.000000	5.872441
N	1.206710	-0.031915	8.113035
N	-1.206710	0.031915	8.113035
N	0.000000	0.000000	10.284140
N	1.205741	0.054829	12.525031
N	-1.205741	-0.054829	12.525031
N	0.000000	0.000000	14.696093
N	1.200788	-0.190181	16.929336
N	-1.200788	0.190181	16.929336
N	0.000000	0.000000	19.045650
H	2.267124	0.403009	-16.377476
H	-2.267124	-0.403009	-16.377476
H	2.059860	0.364623	-18.844690
H	-2.059860	-0.364623	-18.844690
H	0.000000	0.000000	-20.305139
B	1.218403	0.212537	-16.929885
B	-1.218403	-0.212537	-16.929885
B	0.000000	0.000000	-19.107080
N	0.000000	0.000000	-16.186703
N	1.192391	0.208785	-18.352162
N	-1.192391	-0.208785	-18.352162

Supplementary Table 39 | Nuclear coordinates of S_0 geometry of **6i: [10]PP-BN** at the PBE0/6-31G(d) level of theory.

Element Symbol	*x* (Å)	*y* (Å)	*z* (Å)
H	2.297539	0.117403	16.696607
H	2.091172	0.104742	14.248175
H	-2.091172	-0.104742	14.248175
H	-2.297539	-0.117403	16.696607
H	2.300849	-0.067517	12.282217
H	-2.300849	0.067517	12.282217
H	2.092962	-0.060206	9.835825
H	-2.092962	0.060206	9.835825
H	-2.069599	0.324939	18.639064
H	2.069599	-0.324939	18.639064
H	-2.264268	0.352961	21.153199
H	2.264268	-0.352961	21.153199

H	0.000000	0.000000	22.259801
H	-2.302176	0.004622	7.869843
H	2.302176	-0.004622	7.869843
H	-2.093822	0.004585	5.423956
H	2.093822	-0.004585	5.423956
H	2.302253	-0.004557	3.458313
H	-2.302253	0.004557	3.458313
H	2.093847	-0.004085	1.012413
H	-2.093847	0.004085	1.012413
H	-2.302261	-0.007007	-0.953028
H	2.302261	0.007007	-0.953028
H	-2.093897	-0.006378	-3.398868
H	2.093897	0.006378	-3.398868
H	2.302155	0.022135	-5.364398
H	-2.302155	-0.022135	-5.364398
H	2.093934	0.020083	-7.809971
H	-2.093934	-0.020083	-7.809971
H	-2.301735	-0.048173	-9.775862
H	2.301735	0.048173	-9.775862
H	-2.093822	-0.043967	-12.220970
H	2.093822	0.043967	-12.220970
B	0.000000	0.000000	-12.515791
B	1.231370	0.024975	-10.321565
B	-1.231370	-0.024975	-10.321565
B	0.000000	0.000000	-8.105103
B	1.231610	0.011780	-5.910432
B	-1.231610	-0.011780	-5.910432
B	0.000000	0.000000	-3.694023
B	1.231672	0.003692	-1.499190
B	-1.231672	-0.003692	-1.499190
B	0.000000	0.000000	0.717247
B	1.231617	-0.002397	2.912182
B	-1.231617	0.002397	2.912182
B	0.000000	0.000000	5.128797
B	1.231462	-0.003114	7.323838
B	-1.231462	0.003114	7.323838
B	0.000000	0.000000	9.540863
B	1.230715	-0.033964	11.736036
B	-1.230715	0.033964	11.736036
B	0.000000	0.000000	13.953756
B	1.228861	0.057551	16.149309
B	-1.228861	-0.057551	16.149309
B	0.000000	0.000000	18.370403
B	1.233599	-0.191041	20.557854
B	-1.233599	0.191041	20.557854
N	1.207013	0.024288	-11.738948
N	-1.207013	-0.024288	-11.738948
N	0.000000	0.000000	-9.567855
N	1.207203	0.011635	-7.327448
N	-1.207203	-0.011635	-7.327448

N	0.000000	0.000000	-5.156376
N	1.207265	0.003693	-2.916081
N	-1.207265	-0.003693	-2.916081
N	0.000000	0.000000	-0.745027
N	1.207251	-0.002294	1.495265
N	-1.207251	0.002294	1.495265
N	0.000000	0.000000	3.666345
N	1.207199	-0.003001	5.906726
N	-1.207199	0.003001	5.906726
N	0.000000	0.000000	8.077863
N	1.206690	-0.033240	10.318412
N	-1.206690	0.033240	10.318412
N	0.000000	0.000000	12.489484
N	1.205661	0.056471	14.730350
N	-1.205661	-0.056471	14.730350
N	0.000000	0.000000	16.901444
N	1.201414	-0.186124	19.134722
N	-1.201414	0.186124	19.134722
N	0.000000	0.000000	21.251048
H	2.300266	-0.092533	-14.186657
H	-2.300266	0.092533	-14.186657
H	2.092880	-0.084080	-16.631994
H	-2.092880	0.084080	-16.631994
H	2.277966	0.336275	-18.582532
H	-2.277966	-0.336275	-18.582532
H	2.069706	0.303808	-21.049831
H	-2.069706	-0.303808	-21.049831
H	0.000000	0.000000	-22.510239
B	-1.230842	0.046505	-14.732043
B	1.230842	-0.046505	-14.732043
B	0.000000	0.000000	-16.924857
B	1.224118	0.176652	-19.134986
B	-1.224118	-0.176652	-19.134986
B	0.000000	0.000000	-21.312181
N	0.000000	0.000000	-13.979530
N	-1.206482	0.045042	-16.150360
N	1.206482	-0.045042	-16.150360
N	0.000000	0.000000	-18.391855
N	1.198041	0.173569	-20.557276
N	-1.198041	-0.173569	-20.557276

Supplementary Table 40 | Nuclear coordinates of S_0 geometry of **6j: [11]PP-BN** at the PBE0/6-31G(d) level of theory.

Element Symbol	*x* (Å)	*y* (Å)	*z* (Å)
H	2.297765	0.112160	18.902236
H	2.091402	0.099989	16.453666
H	-2.091402	-0.099989	16.453666
H	-2.297765	-0.112160	18.902236
H	2.300846	-0.068122	14.487679

H	-2.300846	0.068122	14.487679
H	2.092933	-0.060791	12.041311
H	-2.092933	0.060791	12.041311
H	-2.068149	0.333977	20.844438
H	2.068149	-0.333977	20.844438
H	-2.262707	0.362842	23.358642
H	2.262707	-0.362842	23.358642
H	0.000000	0.000000	24.465208
H	-2.302152	0.013728	10.075331
H	2.302152	-0.013728	10.075331
H	-2.093767	0.012837	7.629411
H	2.093767	-0.012837	7.629411
H	2.302194	-0.018133	5.663863
H	-2.302194	0.018133	5.663863
H	2.093751	-0.016441	3.217865
H	-2.093751	0.016441	3.217865
H	-2.302268	0.009387	1.252586
H	2.302268	-0.009387	1.252586
H	-2.093824	0.008560	-1.193427
H	2.093824	-0.008560	-1.193427
H	2.302287	0.001806	-3.158664
H	-2.302287	-0.001806	-3.158664
H	2.093897	0.001633	-5.604575
H	-2.093897	-0.001633	-5.604575
H	-2.302188	-0.019296	-7.569970
H	2.302188	0.019296	-7.569970
H	-2.093944	-0.017460	-10.015616
H	2.093944	0.017460	-10.015616
B	0.000000	0.000000	-10.310703
B	1.231654	0.010311	-8.116053
B	-1.231654	-0.010311	-8.116053
B	0.000000	0.000000	-5.899710
B	1.231725	0.000917	-3.704899
B	-1.231725	-0.000917	-3.704899
B	0.000000	0.000000	-1.488577
B	1.231700	-0.005068	0.706319
B	-1.231700	0.005068	0.706319
B	0.000000	0.000000	2.922705
B	1.231602	-0.009624	5.117671
B	-1.231602	0.009624	5.117671
B	0.000000	0.000000	7.334253
B	1.231449	-0.007914	9.529312
B	-1.231449	0.007914	9.529312
B	0.000000	0.000000	11.746328
B	1.230700	-0.034369	13.941521
B	-1.230700	0.034369	13.941521
B	0.000000	0.000000	16.159260
B	1.229010	0.054740	18.354816
B	-1.229010	-0.054740	18.354816
B	0.000000	0.000000	20.575841

B	1.232768	-0.196419	22.763273
B	-1.232768	0.196419	22.763273
N	1.207232	0.010228	-9.533029
N	-1.207232	-0.010228	-9.533029
N	0.000000	0.000000	-7.361979
N	1.207290	0.000969	-5.121720
N	-1.207290	-0.000969	-5.121720
N	0.000000	0.000000	-2.950693
N	1.207279	-0.004921	-0.710470
N	-1.207279	0.004921	-0.710470
N	0.000000	0.000000	1.460560
N	1.207228	-0.009401	3.700811
N	-1.207228	0.009401	3.700811
N	0.000000	0.000000	5.871876
N	1.207183	-0.007728	8.112235
N	-1.207183	0.007728	8.112235
N	0.000000	0.000000	10.283371
N	1.206675	-0.033657	12.523918
N	-1.206675	0.033657	12.523918
N	0.000000	0.000000	14.695005
N	1.205798	0.053735	16.935868
N	-1.205798	-0.053735	16.935868
N	0.000000	0.000000	19.106919
N	1.200602	-0.191374	21.340147
N	-1.200602	0.191374	21.340147
N	0.000000	0.000000	23.456456
H	2.301256	0.066708	-11.981337
H	-2.301256	-0.066708	-11.981337
H	2.093384	0.060719	-14.426551
H	-2.093384	-0.060719	-14.426551
H	-2.300898	0.074377	-16.392126
H	2.300898	-0.074377	-16.392126
H	-2.093455	0.067089	-18.837569
H	2.093455	-0.067089	-18.837569
H	2.275056	0.355513	-20.788079
H	-2.275056	-0.355513	-20.788079
H	2.067044	0.321427	-23.255330
H	-2.067044	-0.321427	-23.255330
H	0.000000	0.000000	-24.715758
B	-1.231158	-0.034661	-12.527109
B	1.231158	0.034661	-12.527109
B	0.000000	0.000000	-14.721325
B	-1.231188	0.036690	-16.937550
B	1.231188	-0.036690	-16.937550
B	0.000000	0.000000	-19.130332
B	1.222589	0.187019	-21.340521
B	-1.222589	-0.187019	-21.340521
B	0.000000	0.000000	-23.517709
N	0.000000	0.000000	-11.773404
N	-1.206792	-0.033805	-13.944470

N	1.206792	0.033805	-13.944470
N	0.000000	0.000000	-16.185038
N	-1.206799	0.035135	-18.355865
N	1.206799	-0.035135	-18.355865
N	0.000000	0.000000	-20.597343
N	1.196509	0.183792	-22.762787
N	-1.196509	-0.183792	-22.762787

Supplementary Table 41 | Nuclear coordinates of S_0 geometry of **6k: [12]PP-BN** at the PBE0/6-31G(d) level of theory.

Element Symbol	*x* (Å)	*y* (Å)	*z* (Å)
H	-2.297367	-0.120455	21.107669
H	-2.091021	-0.107537	18.659171
H	2.091021	0.107537	18.659171
H	2.297367	0.120455	21.107669
H	-2.301207	0.055148	16.693114
H	2.301207	-0.055148	16.693114
H	-2.093236	0.049000	14.246780
H	2.093236	-0.049000	14.246780
H	2.069759	-0.323837	23.049993
H	-2.069759	0.323837	23.049993
H	2.264454	-0.351767	25.564182
H	-2.264454	0.351767	25.564182
H	0.000000	0.000000	26.670761
H	2.302175	-0.011178	12.280787
H	-2.302175	0.011178	12.280787
H	2.093768	-0.010455	9.834852
H	-2.093768	0.010455	9.834852
H	-2.302229	0.013785	7.869368
H	2.302229	-0.013785	7.869368
H	-2.093759	0.012496	5.423307
H	2.093759	-0.012496	5.423307
H	2.302287	-0.005935	3.458134
H	-2.302287	0.005935	3.458134
H	2.093801	-0.005420	1.012022
H	-2.093801	0.005420	1.012022
H	-2.302294	0.006135	-0.953052
H	2.302294	-0.006135	-0.953052
H	-2.093829	0.005535	-3.399128
H	2.093829	-0.005535	-3.399128
H	2.302295	0.000339	-5.364249
H	-2.302295	-0.000339	-5.364249
H	2.093892	0.000369	-7.810196
H	-2.093892	-0.000369	-7.810196
B	0.000000	0.000000	-8.105338
B	-1.231752	-0.000150	-5.910531
B	1.231752	0.000150	-5.910531
B	0.000000	0.000000	-3.694276
B	-1.231755	0.003247	-1.499397

B	1.231755	-0.003247	-1.499397
B	0.000000	0.000000	0.716870
B	-1.231727	0.003256	2.911799
B	1.231727	-0.003256	2.911799
B	0.000000	0.000000	5.128150
B	-1.231632	0.007318	7.323132
B	1.231632	-0.007318	7.323132
B	0.000000	0.000000	9.539688
B	-1.231464	0.006431	11.734760
B	1.231464	-0.006431	11.734760
B	0.000000	0.000000	13.951770
B	-1.230869	0.027515	16.146979
B	1.230869	-0.027515	16.146979
B	0.000000	0.000000	18.364731
B	-1.228790	-0.059214	20.560304
B	1.228790	0.059214	20.560304
B	0.000000	0.000000	22.781364
B	-1.233707	0.190388	24.968813
B	1.233707	-0.190388	24.968813
N	-1.207300	-0.000212	-7.327308
N	1.207300	0.000212	-7.327308
N	0.000000	0.000000	-5.156296
N	-1.207304	0.003144	-2.916113
N	1.207304	-0.003144	-2.916113
N	0.000000	0.000000	-0.745109
N	-1.207296	0.003170	1.495070
N	1.207296	-0.003170	1.495070
N	0.000000	0.000000	3.666084
N	-1.207251	0.007140	5.906310
N	1.207251	-0.007140	5.906310
N	0.000000	0.000000	8.077363
N	-1.207194	0.006282	10.317707
N	1.207194	-0.006282	10.317707
N	0.000000	0.000000	12.488844
N	-1.206840	0.026951	14.729394
N	1.206840	-0.026951	14.729394
N	0.000000	0.000000	16.900495
N	-1.205585	-0.058132	19.141369
N	1.205585	0.058132	19.141369
N	0.000000	0.000000	21.312446
N	-1.201520	0.185493	23.545695
N	1.201520	-0.185493	23.545695
N	0.000000	0.000000	25.662009
H	-2.302270	-0.005314	-9.775516
H	2.302270	0.005314	-9.775516
H	-2.093999	-0.004803	-12.221221
H	2.093999	0.004803	-12.221221
H	2.301398	0.061428	-14.186812
H	-2.301398	-0.061428	-14.186812
H	2.093509	0.055829	-16.632097

H	-2.093509	-0.055829	-16.632097
H	-2.300622	0.082292	-18.597596
H	2.300622	-0.082292	-18.597596
H	-2.093203	0.074279	-21.043064
H	2.093203	-0.074279	-21.043064
H	-2.276316	-0.347398	-22.993576
H	2.276316	0.347398	-22.993576
H	-2.068165	-0.314080	-25.460842
H	2.068165	0.314080	-25.460842
H	0.000000	0.000000	-26.921248
B	1.231716	0.003002	-10.321633
B	-1.231716	-0.003002	-10.321633
B	0.000000	0.000000	-12.516269
B	1.231256	0.031735	-14.732633
B	-1.231256	-0.031735	-14.732633
B	0.000000	0.000000	-16.926839
B	1.231061	-0.040919	-19.143046
B	-1.231061	0.040919	-19.143046
B	0.000000	0.000000	-21.335839
B	-1.223261	-0.182661	-23.546028
B	1.223261	0.182661	-23.546028
B	0.000000	0.000000	-25.723202
N	0.000000	0.000000	-9.567549
N	1.207284	0.003068	-11.738581
N	-1.207284	-0.003068	-11.738581
N	0.000000	0.000000	-13.978927
N	1.206878	0.030942	-16.149975
N	-1.206878	-0.030942	-16.149975
N	0.000000	0.000000	-18.390522
N	1.206661	-0.039289	-20.561348
N	-1.206661	0.039289	-20.561348
N	0.000000	0.000000	-22.802837
N	-1.197156	-0.179543	-24.968279
N	1.197156	0.179543	-24.968279

Supplementary Table 42 | Nuclear coordinates of S_0 geometry of **7a: [3]MP-BN** at the PBE0/6-31G(d) level of theory.

Element Symbol	*x* (Å)	*y* (Å)	*z* (Å)
H	0.708887	1.600647	-1.979572
H	-0.707833	3.796889	1.807010
H	-0.643116	5.834512	0.396027
H	0.000179	6.099953	-2.059310
H	0.643391	3.836773	-3.047482
H	0.001601	2.089543	2.696568
H	0.000000	0.000000	-0.933008
H	0.000000	0.000000	4.116440
H	-0.001601	-2.089543	2.696568
H	-0.708887	-1.600647	-1.979572
H	0.707833	-3.796889	1.807010

H	-0.643391	-3.836773	-3.047482
H	0.643116	-5.834512	0.396027
H	-0.000179	-6.099953	-2.059310
B	0.000043	5.062788	-1.457580
B	-0.375040	3.768456	0.653482
B	0.375221	2.587390	-1.382102
B	-0.002019	1.262770	0.749802
B	0.002019	-1.262770	0.749802
B	0.000000	0.000000	2.915471
B	-0.375221	-2.587390	-1.382102
B	0.375040	-3.768456	0.653482
B	-0.000043	-5.062788	-1.457580
N	-0.368954	4.988871	-0.082258
N	0.000000	2.537496	0.007296
N	0.369021	3.832100	-2.076025
N	-0.003345	1.221416	2.182461
N	0.000000	0.000000	0.076575
N	0.003345	-1.221416	2.182461
N	0.000000	-2.537496	0.007296
N	-0.369021	-3.832100	-2.076025
N	0.368954	-4.988871	-0.082258

Supplementary Table 43 | Nuclear coordinates of S_0 geometry of **7b: [3]MP-NB** at the PBE0/6-31G(d) level of theory.

Element Symbol	*x* (Å)	*y* (Å)	*z* (Å)
H	0.805294	1.786503	-1.788589
H	-0.796007	3.735719	1.558572
H	-0.860269	6.002547	0.453778
H	0.009757	5.890463	-1.934743
H	0.878704	3.868378	-3.211389
H	-0.020013	2.265382	2.838758
H	0.000000	0.000000	-1.133594
H	0.000000	0.000000	3.885923
H	0.020013	-2.265382	2.838758
H	-0.805294	-1.786503	-1.788589
H	0.796007	-3.735719	1.558572
H	-0.878704	-3.868378	-3.211389
H	0.860269	-6.002547	0.453778
H	-0.009757	-5.890463	-1.934743
N	0.007040	5.018029	-1.427943
N	-0.455981	3.755749	0.608303
N	0.461814	2.622148	-1.338382
N	-0.004824	1.260125	0.759927
N	0.004824	-1.260125	0.759927
N	0.000000	0.000000	2.876187
N	-0.461814	-2.622148	-1.338382
N	0.455981	-3.755749	0.608303
N	-0.007040	-5.018029	-1.427943
B	-0.465228	5.003389	-0.081201

B	0.000000	2.533222	0.015232
B	0.478396	3.839360	-2.080292
B	-0.013743	1.250719	2.197874
B	0.000000	0.000000	0.067757
B	0.013743	-1.250719	2.197874
B	0.000000	-2.533222	0.015232
B	-0.478396	-3.839360	-2.080292
B	0.465228	-5.003389	-0.081201

Supplementary Table 44 | Nuclear coordinates of S_0 geometry of **7c: [4]MP-BN** at the PBE0/6-31G(d) level of theory.

Element Symbol	*x* (Å)	*y* (Å)	*z* (Å)
H	3.615210	-1.780694	1.050092
H	5.563232	1.982933	-0.742979
H	7.712265	0.862584	-0.223382
H	8.150524	-1.411401	0.848892
H	5.940614	-2.560341	1.403909
H	3.697848	2.748496	-0.412611
H	2.009455	-1.057252	0.042011
H	1.489346	3.880001	-0.882263
H	-0.427934	2.233515	-0.835929
H	0.618806	-2.371986	-0.812900
H	-2.027819	1.253699	0.055294
H	-1.512701	-3.669371	-0.775030
H	-3.859563	-2.913444	-0.390144
B	7.064471	-0.950073	0.636570
B	5.613739	0.900631	-0.225258
B	4.567602	-1.124262	0.727317
B	3.084948	0.742604	-0.143206
B	0.590949	0.435441	-0.387923
B	1.612987	2.706416	-0.661123
B	-0.449346	-1.864547	-0.608842
B	-1.904608	0.075275	-0.142973
B	-2.922923	-2.162823	-0.381481
N	6.893625	0.331597	0.035394
N	4.419368	0.173243	0.119814
N	5.868615	-1.651354	0.970753
N	2.894353	2.138171	-0.408769
N	1.906082	-0.070180	-0.143631
N	0.481300	1.840138	-0.644292
N	-0.589232	-0.452333	-0.380132
N	-1.616772	-2.679107	-0.607638
N	-3.079312	-0.754967	-0.134178
H	-5.536907	-1.722494	-0.778693
H	-3.763287	1.564117	1.123033
H	-7.896819	-0.903743	-0.426327
H	-5.956755	2.697272	1.642710
H	-7.926970	1.326367	0.798628
B	-4.411883	-0.179056	0.123231

B	-6.917429	-0.315481	-0.058036
B	-5.859854	1.649179	1.065793
N	-5.613599	-0.842093	-0.290812
N	-7.013705	0.935124	0.622972
N	-4.583841	1.071078	0.802711

Supplementary Table 45 | Nuclear coordinates of S_0 geometry of **7d: [5]MP-BN** at the PBE0/6-31G(d) level of theory.

Element Symbol	*x* (Å)	*y* (Å)	*z* (Å)
H	1.122476	-1.461915	2.434701
H	-0.290337	-3.796525	-1.234227
H	0.985021	-5.988390	2.571663
H	1.408582	-3.706160	3.487788
H	0.332965	-2.063113	-2.184984
H	0.000000	0.000000	1.444737
H	0.000000	0.000000	-3.605952
H	-0.332965	2.063113	-2.184984
H	-1.122476	1.461915	2.434701
H	0.290337	3.796525	-1.234227
H	-1.408582	3.706160	3.487788
H	-0.985021	5.988390	2.571663
B	0.801284	-4.986524	1.936856
B	0.115701	-3.775072	-0.103884
B	0.871576	-2.486883	1.863018
B	0.192773	-1.247074	-0.239723
B	-0.192773	1.247074	-0.239723
B	0.000000	0.000000	-2.405674
B	-0.871576	2.486883	1.863018
B	-0.115701	3.775072	-0.103884
B	-0.801284	4.986524	1.936856
N	0.305828	-5.021689	0.587449
N	0.393474	-2.504075	0.507298
N	1.066338	-3.724519	2.538026
N	0.186531	-1.206927	-1.671343
N	0.000000	0.000000	0.434923
N	-0.186531	1.206927	-1.671343
N	-0.393474	2.504075	0.507298
N	-1.066338	3.724519	2.538026
N	-0.305828	5.021689	0.587449
H	-1.216563	7.518854	1.159916
H	1.330745	5.553427	-1.526124
H	-0.814099	9.805799	0.174881
H	1.961790	7.654687	-2.771187
H	0.799869	9.694172	-1.789190
B	0.000000	6.306157	-0.069348
B	-0.304169	8.798067	-0.231468
B	1.205318	7.625063	-1.839846
N	-0.577551	7.539160	0.378716
N	0.591872	8.813845	-1.342242

N	0.891470	6.396928	-1.187718
H	1.216563	-7.518854	1.159916
H	-1.330745	-5.553427	-1.526124
H	0.814099	-9.805799	0.174881
H	-1.961790	-7.654687	-2.771187
H	-0.799869	-9.694172	-1.789190
B	0.000000	-6.306157	-0.069348
B	0.304169	-8.798067	-0.231468
B	-1.205318	-7.625063	-1.839846
N	0.577551	-7.539160	0.378716
N	-0.891470	-6.396928	-1.187718
N	-0.591872	-8.813845	-1.342242

Supplementary Table 46 | Nuclear coordinates of S_0 geometry of **7e: [5]MP-NB** at the PBE0/6-31G(d) level of theory.

Element Symbol	*x* (Å)	*y* (Å)	*z* (Å)
H	-1.092574	-1.636228	-2.290151
H	0.225463	-3.794719	1.047795
H	-0.927759	-5.809953	-2.437943
H	-1.491714	-3.696115	-3.700257
H	-0.332049	-2.240922	2.333813
H	0.000000	0.000000	-1.638420
H	0.000000	0.000000	3.380324
H	0.332049	2.240922	2.333813
H	1.092574	1.636228	-2.290151
H	-0.225463	3.794719	1.047795
H	1.491714	3.696115	-3.700257
H	0.927759	5.809953	-2.437943
N	-0.789789	-4.953357	-1.922902
N	-0.118291	-3.775625	0.098616
N	-0.875037	-2.513507	-1.841515
N	-0.190279	-1.245850	0.254820
N	0.190279	1.245850	0.254820
N	0.000000	0.000000	2.370679
N	0.875037	2.513507	-1.841515
N	0.118291	3.775625	0.098616
N	0.789789	4.953357	-1.922902
B	-0.301369	-5.024267	-0.577264
B	-0.391815	-2.502474	-0.493193
B	-1.082204	-3.719524	-2.571811
B	-0.180251	-1.238005	1.692150
B	0.000000	0.000000	-0.437045
B	0.180251	1.238005	1.692150
B	0.391815	2.502474	-0.493193
B	1.082204	3.719524	-2.571811
B	0.301369	5.024267	-0.577264
H	1.218164	7.568585	-1.427817
H	-1.294930	5.380676	1.745254
H	0.603542	9.627941	-0.192000

H	-1.680015	7.637873	2.694050
H	-0.834164	9.909925	1.897604
N	0.000000	6.311421	0.076274
N	0.219294	8.779401	0.197138
N	-1.096358	7.627402	1.870450
B	0.520036	7.546630	-0.450974
B	-0.591272	8.862385	1.367311
B	-0.820299	6.370605	1.258533
H	-1.218164	-7.568585	-1.427817
H	1.294930	-5.380676	1.745254
H	-0.603542	-9.627941	-0.192000
H	1.680015	-7.637873	2.694050
H	0.834164	-9.909925	1.897604
N	0.000000	-6.311421	0.076274
N	-0.219294	-8.779401	0.197138
N	1.096358	-7.627402	1.870450
B	-0.520036	-7.546630	-0.450974
B	0.820299	-6.370605	1.258533
B	0.591272	-8.862385	1.367311

Supplementary Table 47 | Nuclear coordinates of S_0 geometry of **7f: [6]MP-BN** at the PBE0/6-31G(d) level of theory.

Element Symbol	*x* (Å)	*y* (Å)	*z* (Å)
H	0.294821	2.435603	0.437463
H	-1.845412	-1.512352	-0.427335
H	-4.217545	2.368862	0.037169
H	-1.991974	3.433340	0.412682
H	-0.125557	-2.274279	0.427945
H	1.850629	1.324599	-0.397635
H	1.993965	-3.650725	0.458968
H	4.032775	-2.227509	0.006900
H	3.351347	2.166349	-1.590062
H	5.696074	-1.118009	0.559962
H	5.577886	3.243437	-1.921185
H	7.854105	2.501921	-1.216281
B	-3.188854	1.750888	0.022528
B	-1.880470	-0.328541	-0.226374
B	-0.696341	1.788735	0.237347
B	0.641828	-0.350114	0.006844
B	3.154411	-0.319153	-0.231204
B	1.959753	-2.468247	0.255430
B	4.374236	1.685644	-1.186503
B	5.667748	-0.073842	-0.033333
B	6.861196	1.868322	-0.985403
N	-3.154991	0.335851	-0.227420
N	-0.645407	0.370774	0.006046
N	-1.962200	2.438381	0.243548
N	0.723490	-1.760039	0.246161
N	1.879475	0.329802	-0.226636

N	3.154949	-1.729882	0.015538
N	4.400108	0.431616	-0.484090
N	5.601895	2.366330	-1.421581
N	6.903657	0.623975	-0.266091
H	9.435895	1.131370	-1.115918
H	7.390683	-1.159843	1.735902
H	11.720239	0.319309	-0.419088
H	9.482740	-2.196286	2.691788
H	11.562810	-1.345759	1.498637
B	8.184279	0.079170	0.221189
B	10.694185	-0.034052	0.093844
B	9.474367	-1.408398	1.786395
N	9.438196	0.471018	-0.352395
N	10.684985	-0.975456	1.166542
N	8.250434	-0.867782	1.294850
H	-5.694784	0.945848	0.463283
H	-3.526551	-2.036927	-1.522268
H	-5.564350	-3.443707	-2.030224
H	-7.674168	-2.379314	-1.138648
B	-4.399510	-0.418029	-0.478995
B	-6.906459	-0.617641	-0.256254
B	-5.595299	-2.400735	-1.436265
N	-5.669264	0.068613	-0.036000
N	-4.400831	-1.666462	-1.180841
N	-6.826388	-1.859880	-0.966732
H	-9.478243	-1.416400	-1.142685
H	-7.228846	1.401675	1.715936
H	-11.529812	-0.383795	-0.210050
H	-9.484104	2.179675	2.387452
H	-11.781185	1.433865	1.563544
B	-8.232266	0.955902	1.229511
B	-10.735958	0.994400	1.173861
B	-9.440494	-0.564352	-0.297321
N	-8.190445	-0.075757	0.225377
N	-9.486478	1.461754	1.677778
N	-10.670214	-0.025819	0.179839

Supplementary Table 48 | Nuclear coordinates of S_0 geometry of **8a: $B_{12}N_{12}$-fullerene** at the PBE0/6-31G(d) level of theory.

Element Symbol	*x* (Å)	*y* (Å)	*z* (Å)
N	0.000000	-2.122559	1.122921
N	2.122559	-1.122921	0.000000
N	0.000000	-2.122559	-1.122921
N	2.122559	1.122921	0.000000
N	1.122921	0.000000	-2.122559
N	0.000000	2.122559	-1.122921
N	0.000000	2.122559	1.122921
N	1.122921	0.000000	2.122559
N	-2.122559	-1.122921	0.000000

N	-2.122559	1.122921	0.000000
N	-1.122921	0.000000	2.122559
N	-1.122921	0.000000	-2.122559
B	0.000000	-0.951953	1.955961
B	1.955961	0.000000	0.951953
B	0.000000	0.951953	1.955961
B	-1.955961	0.000000	0.951953
B	0.951953	1.955961	0.000000
B	-0.951953	1.955961	0.000000
B	0.000000	0.951953	-1.955961
B	-1.955961	0.000000	-0.951953
B	1.955961	0.000000	-0.951953
B	0.000000	-0.951953	-1.955961
B	-0.951953	-1.955961	0.000000
B	0.951953	-1.955961	0.000000

Supplementary Table 49 | Nuclear coordinates of S_0 geometry of **8b: $B_{24}N_{24}$-fullerene** at the PBE0/6-31G(d) level of theory.

Element Symbol	*x* (Å)	*y* (Å)	*z* (Å)
B	-1.716159	0.685108	2.682756
B	0.685108	1.716159	2.682756
B	1.716159	-0.685108	2.682756
B	-0.685108	-1.716159	2.682756
B	-0.685108	2.682756	1.716159
B	1.716159	2.682756	0.685108
B	2.682756	0.685108	1.716159
B	2.682756	-1.716159	0.685108
B	0.685108	-2.682756	1.716159
B	-1.716159	-2.682756	0.685108
B	-2.682756	-0.685108	1.716159
B	-2.682756	1.716159	0.685108
B	2.682756	1.716159	-0.685108
B	2.682756	-0.685108	-1.716159
B	1.716159	-2.682756	-0.685108
B	-0.685108	-2.682756	-1.716159
B	0.685108	-1.716159	-2.682756
B	1.716159	0.685108	-2.682756
B	0.685108	2.682756	-1.716159
B	-0.685108	1.716159	-2.682756
B	-1.716159	-0.685108	-2.682756
B	-2.682756	0.685108	-1.716159
B	-2.682756	-1.716159	-0.685108
B	-1.716159	2.682756	-0.685108
N	-2.853303	1.725917	-0.769227
N	-1.725917	2.853303	0.769227
N	-0.769227	2.853303	-1.725917
N	0.769227	-2.853303	-1.725917
N	1.725917	2.853303	-0.769227
N	0.769227	2.853303	1.725917

N	1.725917	-2.853303	0.769227
N	1.725917	0.769227	2.853303
N	2.853303	-0.769227	1.725917
N	2.853303	1.725917	0.769227
N	2.853303	0.769227	-1.725917
N	1.725917	-0.769227	-2.853303
N	0.769227	1.725917	-2.853303
N	-1.725917	0.769227	-2.853303
N	-2.853303	-0.769227	-1.725917
N	-2.853303	-1.725917	0.769227
N	-2.853303	0.769227	1.725917
N	-1.725917	-2.853303	-0.769227
N	-1.725917	-0.769227	2.853303
N	-0.769227	1.725917	2.853303
N	-0.769227	-2.853303	1.725917
N	0.769227	-1.725917	2.853303
N	2.853303	-1.725917	-0.769227
N	-0.769227	-1.725917	-2.853303

Supplementary Table 50 | Nuclear coordinates of S_0 geometry of **8c: $B_{36}N_{36}$-fullerene** at the PBE0/6-31G(d) level of theory.

Element Symbol	*x* (Å)	*y* (Å)	*z* (Å)
B	-0.645776	-0.645776	4.091662
B	0.645776	0.645776	4.091662
B	-1.432900	1.432900	3.202172
B	-1.432900	3.202172	1.432900
B	0.645776	4.091662	0.645776
B	0.712192	2.483859	2.483859
B	2.483859	0.712192	2.483859
B	1.432900	-1.432900	3.202172
B	3.202172	-1.432900	1.432900
B	4.091662	0.645776	0.645776
B	2.483859	2.483859	0.712192
B	1.432900	-3.202172	1.432900
B	-0.645776	-4.091662	0.645776
B	-0.712192	-2.483859	2.483859
B	-2.483859	-2.483859	0.712192
B	-2.483859	-0.712192	2.483859
B	-4.091662	-0.645776	0.645776
B	-3.202172	1.432900	1.432900
B	-4.091662	0.645776	-0.645776
B	-2.483859	2.483859	-0.712192
B	-0.645776	4.091662	-0.645776
B	1.432900	3.202172	-1.432900
B	-0.712192	2.483859	-2.483859
B	-2.483859	0.712192	-2.483859
B	-3.202172	-1.432900	-1.432900
B	-1.432900	-1.432900	-3.202172
B	-1.432900	-3.202172	-1.432900

B	0.645776	-4.091662	-0.645776
B	0.712192	-2.483859	-2.483859
B	0.645776	-0.645776	-4.091662
B	-0.645776	0.645776	-4.091662
B	1.432900	1.432900	-3.202172
B	3.202172	1.432900	-1.432900
B	2.483859	-0.712192	-2.483859
B	2.483859	-2.483859	-0.712192
B	4.091662	-0.645776	-0.645776
N	-1.431092	3.199064	-1.431092
N	0.759343	2.558516	-2.558516
N	-1.431092	1.431092	-3.199064
N	-3.199064	1.431092	-1.431092
N	-2.558516	2.558516	0.759343
N	-0.789970	4.310153	0.789970
N	-0.759343	2.558516	2.558516
N	-4.310153	0.789970	0.789970
N	-1.431092	-3.199064	1.431092
N	-3.199064	-1.431092	1.431092
N	-4.310153	-0.789970	-0.789970
N	-2.558516	-0.759343	-2.558516
N	0.789970	-4.310153	0.789970
N	-0.789970	-0.789970	-4.310153
N	0.789970	0.789970	-4.310153
N	1.431092	-1.431092	-3.199064
N	1.431092	-3.199064	-1.431092
N	-0.759343	-2.558516	-2.558516
N	-0.789970	-4.310153	-0.789970
N	-2.558516	-2.558516	-0.759343
N	2.558516	-2.558516	0.759343
N	0.759343	-2.558516	2.558516
N	-2.558516	0.759343	2.558516
N	-0.789970	0.789970	4.310153
N	-1.431092	-1.431092	3.199064
N	0.789970	-0.789970	4.310153
N	1.431092	1.431092	3.199064
N	2.558516	-0.759343	2.558516
N	3.199064	1.431092	1.431092
N	4.310153	-0.789970	0.789970
N	4.310153	0.789970	-0.789970
N	3.199064	-1.431092	-1.431092
N	2.558516	2.558516	-0.759343
N	2.558516	0.759343	-2.558516
N	0.789970	4.310153	-0.789970
N	1.431092	3.199064	1.431092

Supplementary Table 51 | Nuclear coordinates of S_0 geometry of **9: ben-BS** at the PBE0/6-31G(d) level of theory.

Element Symbol	*x* (Å)	*y* (Å)	*z* (Å)
S	-1.634315	0.943572	0.000000
S	1.634315	0.943572	0.000000
S	0.000000	-1.887144	0.000000
B	0.000000	1.700963	0.000000
H	0.000000	2.893645	0.000000
B	-1.473077	-0.850482	0.000000
H	-2.505970	-1.446822	0.000000
B	1.473077	-0.850482	0.000000
H	2.505970	-1.446822	0.000000

Supplementary Table 52 | Nuclear coordinates of S_0 geometry of **10: ben-BSe** at the PBE0/6-31G(d) level of theory.

Element Symbol	*x* (Å)	*y* (Å)	*z* (Å)
Se	-1.744646	1.007272	0.000000
Se	1.744646	1.007272	0.000000
Se	0.000000	-2.014543	0.000000
B	0.000000	1.797228	0.000000
H	0.000000	2.990142	0.000000
B	-1.556445	-0.898614	0.000000
H	-2.589539	-1.495071	0.000000
B	1.556445	-0.898614	0.000000
H	2.589539	-1.495071	0.000000

Supplementary Table 53 | Nuclear coordinates of S_0 geometry of **11: ben-BO** at the PBE0/6-31G(d) level of theory.

Element Symbol	*x* (Å)	*y* (Å)	*z* (Å)
O	0.000000	1.372903	0.000000
O	1.188969	-0.686451	0.000000
O	-1.188969	-0.686451	0.000000
B	1.189344	0.686668	0.000000
H	2.221725	1.282713	0.000000
B	-1.189344	0.686668	0.000000
H	-2.221725	1.282713	0.000000
B	0.000000	-1.373336	0.000000
H	0.000000	-2.565427	0.000000

Supplementary Table 54 | Nuclear coordinates of S_0 geometry of **12: ben-GaN** at the PBE0/6-31G(d) level of theory.

Element Symbol	*x* (Å)	*y* (Å)	*z* (Å)
N	0.000000	1.772453	0.000000
H	0.000000	2.786042	0.000000
N	1.534989	-0.886227	0.000000
H	2.412783	-1.393021	0.000000
N	-1.534989	-0.886227	0.000000

H	-2.412783	-1.393021	0.000000
H	2.984445	1.723070	0.000000
H	-2.984445	1.723070	0.000000
H	0.000000	-3.446141	0.000000
Ga	-1.640907	0.947378	0.000000
Ga	0.000000	-1.894756	0.000000
Ga	1.640907	0.947378	0.000000

Supplementary Table 55 | Nuclear coordinates of S_0 geometry of **13: prism-GaN** at the PBE0/6-31G(d) level of theory.

Element Symbol	*x* (Å)	*y* (Å)	*z* (Å)
H	-1.210354	1.895099	1.120418
H	-0.517124	-1.163737	-2.684268
H	1.881855	-1.310704	0.000000
H	-0.517124	-1.163737	2.684268
H	2.232208	2.378104	0.000000
H	-1.210354	1.895099	-1.120418
Ga	-0.607977	-0.834382	1.155810
Ga	-0.607977	-0.834382	-1.155810
Ga	1.219467	1.210072	0.000000
N	1.106241	-0.658325	0.000000
N	-0.607977	1.164114	-0.756670
N	-0.607977	1.164114	0.756670

Supplementary Table 56 | Nuclear coordinates of S_0 geometry of **14: ben-B_4N_2** at the PBE0/6-31G(d) level of theory.

Element Symbol	*x* (Å)	*y* (Å)	*z* (Å)
H	0.000000	2.247250	1.545980
H	0.000000	-2.247250	1.545980
N	0.000000	0.000000	-1.546204
H	0.000000	0.000000	-2.561292
H	0.000000	0.000000	2.561292
H	0.000000	2.247250	-1.545980
B	0.000000	-1.254354	-0.864431
H	0.000000	-2.247250	-1.545980
B	0.000000	1.254354	-0.864431
B	0.000000	1.254354	0.864431
B	0.000000	-1.254354	0.864431
N	0.000000	0.000000	1.546204

Supplementary Table 57 | Nuclear coordinates of S_0 geometry of **15: ben-BP** at the PBE0/6-31G(d) level of theory.

Element Symbol	*x* (Å)	*y* (Å)	*z* (Å)
B	1.615714	0.932833	0.094697
H	2.645063	1.527128	0.186565
B	0.000000	-1.865666	0.094697
H	0.000000	-3.054256	0.186565

B	-1.615714	0.932833	0.094697
H	-2.645063	1.527128	0.186565
H	2.746172	-1.585503	0.238368
H	0.000000	3.171006	0.238368
H	-2.746172	-1.585503	0.238368
P	0.000000	1.799659	-0.059894
P	-1.558550	-0.899829	-0.059894
P	1.558550	-0.899829	-0.059894

Supplementary Table 58 | Nuclear coordinates of S_0 geometry of **16: ben-NP** at the PBE0/6-31G(d) level of theory.

Element Symbol	*x* (Å)	*y* (Å)	*z* (Å)
H	2.181815	1.259672	1.086871
H	2.181815	1.259672	-1.086871
H	-2.181815	1.259672	1.086871
H	-2.181815	1.259672	-1.086871
H	0.000000	-2.519343	1.086871
H	0.000000	-2.519343	-1.086871
P	1.401566	0.809195	0.000000
P	-1.401566	0.809195	0.000000
P	0.000000	-1.618389	0.000000
N	0.000000	1.601164	0.000000
N	-1.386649	-0.800582	0.000000
N	1.386649	-0.800582	0.000000

Supplementary Table 59 | Nuclear coordinates of S_0 geometry of **17: ring-Ga_3P_3** at the PBE0/6-31G(d) level of theory.

Element Symbol	*x* (Å)	*y* (Å)	*z* (Å)
H	-2.802357	-1.617941	-1.144840
H	-2.846120	1.643208	-0.359599
H	0.000000	3.235883	-1.144840
H	0.000000	-3.286417	-0.359599
H	2.802357	-1.617941	-1.144840
H	2.846120	1.643208	-0.359599
Ga	0.000000	1.964182	-0.242233
Ga	1.701031	-0.982091	-0.242233
Ga	-1.701031	-0.982091	-0.242233
P	1.938163	1.118999	0.600910
P	0.000000	-2.237998	0.600910
P	-1.938163	1.118999	0.600910

Supplementary Table 60 | Nuclear coordinates of S_0 geometry of **18: ben-AlN** at the PBE0/6-31G(d) level of theory.

Element Symbol	*x* (Å)	*y* (Å)	*z* (Å)
N	0.000000	1.749891	0.000000
H	0.000000	2.764834	0.000000
N	1.515450	-0.874946	0.000000

H	2.394417	-1.382417	0.000000
N	-1.515450	-0.874946	0.000000
H	-2.394417	-1.382417	0.000000
H	2.972508	1.716178	0.000000
H	-2.972508	1.716178	0.000000
H	0.000000	-3.432356	0.000000
Al	-1.594717	0.920710	0.000000
Al	0.000000	-1.841420	0.000000
Al	1.594717	0.920710	0.000000

Supplementary Table 61 | Nuclear coordinates of S_0 geometry of **19: ring-Al_3P_3** at the PBE0/6-31G(d) level of theory.

Element Symbol	*x* (Å)	*y* (Å)	*z* (Å)
P	0.257968	2.167389	0.000000
Al	-0.314228	1.026741	1.900085
P	-0.314228	-1.281983	-1.907960
Al	-0.314228	1.026741	-1.900085
P	-0.314228	-1.281983	1.907960
Al	0.906056	-1.720434	0.000000
H	-0.596443	1.836284	3.238726
H	-0.452338	3.392746	0.000000
H	-0.596443	1.836284	-3.238726
H	0.652609	-1.469144	-2.935004
H	2.288543	-2.508017	0.000000
H	0.652609	-1.469144	2.935004

Supplementary Table 62 | Nuclear coordinates of S_0 geometry of **20: ring-Al_3As_3** at the PBE0/6-31G(d) level of theory.

Element Symbol	*x* (Å)	*y* (Å)	*z* (Å)
As	0.481823	2.261790	0.000000
Al	-0.335643	1.094129	1.887562
As	-0.335643	-1.296176	-1.972679
Al	-0.335643	1.094129	-1.887562
As	-0.335643	-1.296176	1.972679
Al	0.976372	-1.553078	0.000000
H	-0.744828	1.933618	3.177084
H	-0.320765	3.561474	0.000000
H	-0.744828	1.933618	-3.177084
H	0.810281	-1.343115	-2.992892
H	2.476016	-2.091242	0.000000
H	0.810281	-1.343115	2.992892

Supplementary Table 63 | Nuclear coordinates of S_0 geometry of **21: ring-$(Si(N_2CH_3))_3P_3$** at the PBE0/6-31G(d) level of theory.

Element Symbol	*x* (Å)	*y* (Å)	*z* (Å)
P	2.089301	0.747089	0.000000
P	-1.156951	-0.481899	1.996859

P	-1.156951	-0.481899	-1.996859
Si	0.915371	0.015028	-1.661801
Si	0.915371	0.015028	1.661801
Si	-1.896474	-0.110784	0.000000
C	2.207828	-0.120826	-3.623701
C	2.207828	-0.120826	3.623701
C	-4.164405	0.498178	0.000000
H	1.759988	1.893348	3.497605
H	2.814112	-0.171751	4.529539
H	2.165890	-2.085413	2.992787
H	2.814112	-0.171751	-4.529539
H	2.165890	-2.085413	-2.992787
H	1.759988	1.893348	-3.497605
H	-3.251715	2.345411	0.000000
H	-5.225730	0.751748	0.000000
H	-4.209246	-1.574781	0.000000
N	1.931693	-1.121841	2.818367
N	1.625472	0.959960	3.145359
N	-3.668414	-0.726518	0.000000
N	-3.161849	1.342700	0.000000
N	1.931693	-1.121841	-2.818367
N	1.625472	0.959960	-3.145359

Supplementary Table 64 | Nuclear coordinates of S_0 geometry of **22: ring-(Si(N_2CH_3))$_3$$As_3$** at the PBE0/6-31G(d) level of theory.

Element Symbol	*x* (Å)	*y* (Å)	*z* (Å)
As	0.000124	-2.439831	0.000000
As	0.000104	1.220059	2.113118
As	0.000104	1.220059	-2.113118
Si	-0.000065	-0.983376	-1.703481
Si	-0.000065	-0.983376	1.703481
Si	0.000109	1.966832	0.000000
C	-0.000168	-2.165965	-3.750706
C	-0.000168	-2.165965	3.750706
C	-0.000179	4.331122	0.000000
H	2.016741	-1.940422	3.360149
H	-0.000230	-2.712065	4.695534
H	-2.017008	-1.940579	3.360070
H	-0.000230	-2.712065	-4.695534
H	-2.017008	-1.940579	-3.360070
H	2.016741	-1.940422	-3.360149
H	2.016713	3.880252	0.000000
H	-0.000179	5.422418	0.000000
H	-2.016994	3.880225	0.000000
N	-1.063617	-1.778560	3.080702
N	1.063321	-1.778859	3.080629
N	-1.063622	3.557217	0.000000
N	1.063336	3.557264	0.000000
N	-1.063617	-1.778560	-3.080702

N	1.063321	-1.778859	-3.080629

Supplementary Table 65 | Nuclear coordinates of S_0 geometry of **23: ring-Ge_3N_3** at the PBE0/6-31G(d) level of theory.

Element Symbol	*x* (Å)	*y* (Å)	*z* (Å)
N	0.000000	1.630021	0.000000
Ge	-1.739785	1.004465	0.000000
N	-1.411640	-0.815011	0.000000
Ge	0.000000	-2.008931	0.000000
N	1.411640	-0.815011	0.000000
Ge	1.739785	1.004465	0.000000
H	0.000000	2.647731	0.000000
H	-2.293002	-1.323865	0.000000
H	2.293002	-1.323865	0.000000

Supplementary References

1. Shizu K, Kaji H. Theoretical Determination of Rate Constants from Excited States: Application to Benzophenone. *J. Phys. Chem. A* **125**, 9000–9010 (2021).
2. Shizu K, Kaji H. Quantitative prediction of rate constants and its application to organic emitters. *Nat. Commun.* **15**, 4723 (2024).
